# Molecular signature of different lesion types in the brain white matter of patients with progressive multiple sclerosis

**DOI:** 10.1186/s40478-019-0855-7

**Published:** 2019-12-11

**Authors:** Maria L. Elkjaer, Tobias Frisch, Richard Reynolds, Tim Kacprowski, Mark Burton, Torben A. Kruse, Mads Thomassen, Jan Baumbach, Zsolt Illes

**Affiliations:** 1 Department of Neurology, Odense University Hospital, J.B. Winslowsvej 4, DK-5000 Odense C, Denmark; 2 Institute of Clinical Research, BRIDGE, University of Southern Denmark, Odense, Denmark; 3 Institute of Molecular Medicine, University of Southern Denmark, Odense, Denmark; 4 Department of Mathematics and Computer Science, University of Southern Denmark, Odense, Denmark; 5 Department of Brain Sciences, Imperial College, London, UK; 6 Research Group Computational Systems Medicine, Chair of Experimental Bioinformatics, TUM School of Life Sciences Weihenstephan, Technical University of Munich, Freising-Weihenstephan, Germany; 7 Department of Clinical Genetics, Odense University Hospital, Odense, Denmark; 8 Chair of Experimental Bioinformatics, TUM School of Life Sciences Weihenstephan, Technical University of Munich, Freising-Weihenstephan, Germany

**Keywords:** Multiple sclerosis, Secondary progressive, Human brain lesions, Next-generation RNA sequencing, TGF-beta, CD26/DPP4

## Abstract

To identify pathogenetic markers and potential drivers of different lesion types in the white matter (WM) of patients with progressive multiple sclerosis (PMS), we sequenced RNA from 73 different WM areas. Compared to 25 WM controls, 6713 out of 18,609 genes were significantly differentially expressed in MS tissues (FDR < 0.05). A computational systems medicine analysis was performed to describe the MS lesion endophenotypes. The cellular source of specific molecules was examined by RNAscope, immunohistochemistry, and immunofluorescence. To examine common lesion specific mechanisms, we performed de novo network enrichment based on shared differentially expressed genes (DEGs), and found TGFβ-R2 as a central hub. RNAscope revealed astrocytes as the cellular source of TGFβ-R2 in remyelinating lesions. Since lesion-specific unique DEGs were more common than shared signatures, we examined lesion-specific pathways and de novo networks enriched with unique DEGs. Such network analysis indicated classic inflammatory responses in active lesions; catabolic and heat shock protein responses in inactive lesions; neuronal/axonal specific processes in chronic active lesions. In remyelinating lesions, de novo analyses identified axonal transport responses and adaptive immune markers, which was also supported by the most heterogeneous immunoglobulin gene expression. The signature of the normal-appearing white matter (NAWM) was more similar to control WM than to lesions: only 465 DEGs differentiated NAWM from controls, and 16 were unique. The upregulated marker CD26/DPP4 was expressed by microglia in the NAWM but by mononuclear cells in active lesions, which may indicate a special subset of microglia before the lesion develops, but also emphasizes that omics related to MS lesions should be interpreted in the context of different lesions types. While chronic active lesions were the most distinct from control WM based on the highest number of unique DEGs (*n* = 2213), remyelinating lesions had the highest gene expression levels, and the most different molecular map from chronic active lesions. This may suggest that these two lesion types represent two ends of the spectrum of lesion evolution in PMS. The profound changes in chronic active lesions, the predominance of synaptic/neural/axonal signatures coupled with minor inflammation may indicate end-stage irreversible molecular events responsible for this less treatable phase.

## Introduction

Multiple sclerosis (MS) is a chronic inflammatory, demyelinating and neurodegenerative disease of the CNS. Without treatment, a secondary progressive course (SPMS) develops in about half of the patients [65]. Neuroimaging, treatment responses and pathology all show differences between the early and late phase of MS, indicating that disease mechanisms change during the natural course [31]. Therefore, modern systems medicine approaches may help to increase our understanding of MS progression and to find novel, mechanistic treatment targets.

Inflammatory demyelination affects osmotic homeostasis, energy coupling with oligodendrocytes, and contributes to glutamate excitotoxicity, axonal damage and fibrillary gliosis that may inhibit remyelination [23, 49]. Key elements of the degenerative process are chronic oxidative injury [29], accumulation of mitochondrial damage resulting in chronic cell stress and imbalance of ionic homeostasis [9, 60], microglia activation, and age-related iron accumulation in the brain [61]. As the disease progresses, diffuse changes can be observed in the normal appearing white and grey matter (NAWM, NAGM), and B cell follicle-like cellular aggregates in the meninges contribute to subpial cortical lesions [48, 59, 72].

WM lesions are inherent characteristics of MS from the early phase, and both quantitative and qualitative changes in the WM can be observed as the disease progresses: microglia activation in the NAWM [22], increasing number of chronic active lesions, and decreasing number of remyelinating lesions [16, 69]. B cells are also present in active WM lesions in progressive MS, and the number of plasma cells is higher in lesions from progressive MS compared to acute MS [24, 58, 54, 75].

The lesion evolution and fate in the WM can be classified into distinct groups based on the distribution and density of inflammatory cells and myelin loss [72]. During lesion evolution, active lesions develop from the NAWM and are characterized by myelin breakdown and massive infiltration by macrophages and activated microglia. Lesions may remyelinate [56], and partially remyelinated axons and activated microglia are seen [72]. Lesions can develop into inactive lesions with sharply demarcated hypocellular areas of demyelination and axonal degeneration with little to no inflammatory activity [25, 72]. As the disease progresses, the number of chronic active (smoldering, slowly expanding, mixed active/inactive) lesions with a hypocellular demyelinated core and a rim of activated glia increases [25, 46, 56]. The number of chronic active lesions inversely correlates with the proportion of remyelinating lesions, and patients with more severe disease have a higher proportion of such lesions [56].

**Fig. 1:**
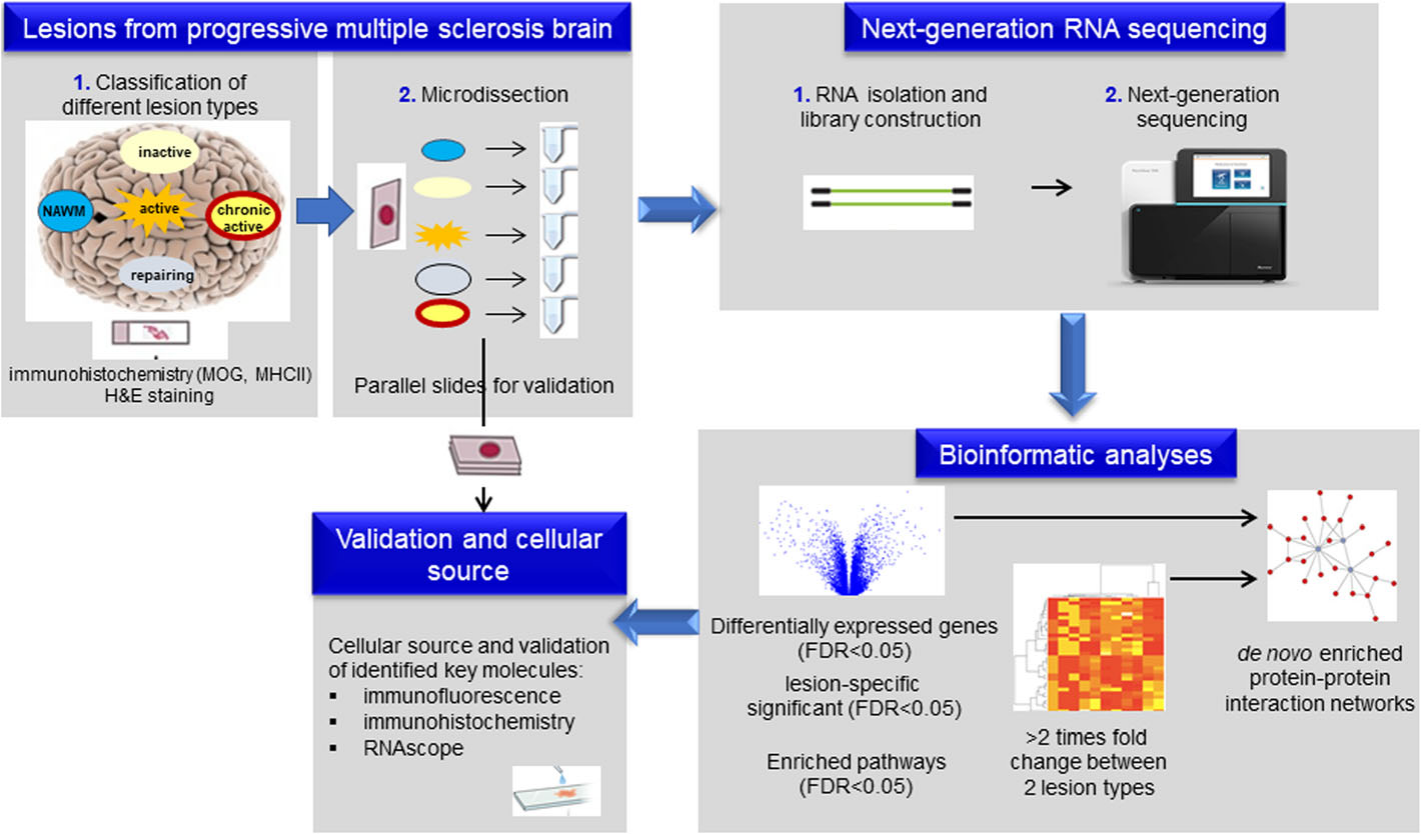
Outline of the systems medicine approach to identify mechanistic drivers of different MS lesion types. Using RNAseq we analysed the transcriptome of normal-appearing white matter (NAWM), and lesion evolution/fate (active, inactive, chronic active, remyelinating) in the white matter (WM) of patients with progressive MS. We performed a comprehensive computational data analysis – from differential expression to de novo network enrichment – and examined selected molecules of interest by a combination of RNAscope, immunohistochemistry and immunofluorescence to confirm their cellular source and protein expression levels

The molecular mechanisms driving the development and evolution of the different cellular MS endophenotypes are largely unknown. To identify dominant pathways of lesion genesis, unbiased omics investigation of precisely defined and microdissected lesions at these different stages of lesion formation and their comparison to controls is required. We addressed this need by generating and analyzing the first tissue map of the transcriptional landscape of lesion evolution and fate in progressive MS brain by deep next-generation RNA sequencing to identify key pathways, molecules and their cellular source (Fig. 1). Two recent studies have performed nuclei-RNA sequencing on MS WM tissue; however, their focus have been on specific cell types, i.e. neurons and oligodendrocytes [39, 74] We performed bulk RNA sequencing that neglects the cell type but major strengths are high coverage from a high number of samples, and analysis of nuclear, cytoplasmic and extracellular RNA both coding and non-coding [19]. Here, we have re-analyzed the data from our original study, since we discovered that a bug in the analysis scripts resulted in incorrect label annotation files for 10 out of the 100 samples [20]. With our comprehensive transcriptomics data, we have been able to extract mechanistic signatures that differentiate between lesions. We identified lesion-specific protein complex networks by using de novo network enrichment. We further validated the differential expression of key molecules and examined their cellular source by RNAscope, immunohistochemistry, and by immunofluorescence. This specific selection and validation of mechanistic signatures in different lesion types emphasize the value of precision in the characterization of the diverse phenotype of lesions, when understanding the complex and heterogeneous pathogenesis of MS.

## Materials and methods

### Human postmortem brain tissue

MS and control tissue samples were supplied by the UK Multiple Sclerosis Society Tissue Bank (UK Multicentre Research Ethics Committee, MREC/02/2/39), funded by the Multiple Sclerosis Society of Great Britain and Northern Ireland (registered charity 207,495). A total of 73 snap-frozen tissue blocks from ten progressive MS patients and 25 blocks from five donors without neurological disease were chosen. The death-tissue preservation interval was between 8 and 30 h. Clinical data are summarized in Additional file 4: Table S1.

### Lesion classification and immunohistochemistry/immunofluorescence

Snap frozen tissue was sectioned (10-μm), fixed (4% PFA), blocked in PBS with 10% normal horse serum (NHS) and stained for myelin oligodendrocyte glycoprotein (MOG) (R. Reynolds, Imperial College, UK) and HLA-DR (Dako UK Ltd) followed by biotinylated secondary antibody (Jackson Immunoresearch Laboratories, Cambridgeshire, UK), avidin/biotin staining (Vector Laboratories, Burlingame, CA) and DAB staining (Vector Laboratories, Burlingame, CA). All sections were also stained with haematoxylin-eosin (H&E) (R. Reynolds, Imperial College, UK). The five pathological areas (NAWM, active, chronic active, inactive and repairing/early remyelinating lesions) were characterized based on MOG+ staining showing myelin integrity and HLA-DR+ staining showing the inflammatory state, and each lesion type was defined as described (Reynolds et al., 2011). All antibodies and concentration are listed in Additional file 1. Quantification of CD20+ cells were performed in four active and three remyelinating lesions from seven different patients. Ten pictures depending on size per lesion type were taken at an objective lens magnification of 20x. The CD20+ cells were manually counted, and the average number of cells per lesion from each patient was compared by using Mann-Whitney Test performed in Graphpad Prism.

#### RNAscope

The RNAscope 2.5 Duplex Assay (ACD Biosystems) was performed according to the ACD protocol for fresh-frozen tissue. Brains sections from one patient with both chronic active and remyelinating lesions and from two patients with either chronic active or remyelinating lesions were hybridized with two mRNA probes per experiment. Hs-GFAP (Cat No. 311801) was used as the astrocyte marker and Hs-AIF1/IBA1 (Cat No. 433121) was used as the microglial marker together with Hs-TGFBR2 (Cat No. 407941). Additionally, the Duplex Negative Control Probes (Cat. No. 320751) was used on one section per slide to confirm signal specificity, and the Duplex Positive Control Probes to confirm sensitivity (Additional file 3: Figure S1). The probes were amplified according to manufacturer’s instructions and labeled with the following red or green color for each experiment. The target probes were also combined with immunohistochemistry (anti-GFAP and anti-MHCII) as described above.

### RNA extraction from specific histological brain areas

The brain fields of interest were manually microdissected under a magnifying glass in a cryostat. The amount of collected tissue ranged between 10 and 100 mg/sample depending on the lesion size and thickness. A total of 25 WM control areas, 19 NAWM, 6 remyelinating, 18 active, 13 inactive and 17 chronic active lesions were harvested. Total RNA was isolated from the frozen brain samples according to the manufacturer’s instruction (miRNeasy Mini Kit, Qiagen) including DNAse I treatment. RNA concentration was measured using NanoDrop spectrophotometer ND-1000 (Thermo Scientific), and the integrity of RNA (RIN) was measured by using the Bioanalyzer 2100 (Agilent Technologies). RNA integrity was good quality (RIN 6 ± 1.7) among the samples, therefore the fragmentation time and cleanup steps during library preparation have been adapted for each sample based on the RIN value.

### Next-generation sequencing

One μg of RNA per sample was processed to remove ribosomal RNA followed by library preparation for RNA sequencing using TruSeq Stranded Total RNA Library Prep Kit with Ribo-Zero Human/Mouse/Rat Set (Illumina). Pooled indexed libraries were loaded into the flow cell followed by 2 × 80 bp paired-end sequencing on an Illumina NextSeq550.

### Raw data analysis and quality control

Demultiplexing was carried out with Casava software (Illumina) configured to allow one mismatch during the identification of the indexes. Data were filtered with Trimmomatic [6] (TRIM:2:30:10 LEADING:20 TRAILING:20 SLIDING:4:20 TRAILING:20 MIN:17). Filtered transcripts were aligned against the human reference genome from UCSC [42] (GRCh38/hg38) with STAR 2.5.3a [14] using default mode/parameters and counted using HTSeq-count [4] using strict mode.

### Statistical analysis

Differentially Expressed Genes (DEGs) between different lesion types vs. control WM were identified with the edgeR package (3.8) [73]. The generalized linear model used for our analysis adjusted for library size and biological replicates (same lesion type//same sample//from same patient). Furthermore, we corrected for age and sex of the patients. Genes that had very low expression were excluded following the edgeR userGuide. Therefore, genes were expected to be presented with more than two counts per million (CPM) in at least as many samples as present in the smallest lesion group. Adjusted *P* value filtering using the procedure of Benjamini and Hochberg was used to identify genes significantly differently expressed between MS brain areas and control brain areas.

### Volcano plots, heatmaps and pathways

Volcano plots and heatmaps were created in R studio, and Venn diagrams were produced using an online tool at http://bioinformatics.psb.ugent.be/webtools/Venn/. Predefined pathways were identified by importing the DEGs of selected gene sets to different enrichment tools using Gene Ontology enRIchment anaLysis and visuaLizAtion tool (GOrilla) [17] WebGestalt [86] and FunRich [67]. Charts were produced using meta-chart.com. KeyPathwayMiner [1, 2] was used to conduct *de-novo* network enrichment analyses. The biological network was selected and downloaded from the Integrated Interactions Database (IID) [44] restricted to only brain specific interactions based on evidence type: experimental detection, orthology or prediction. The network and the gene lists were uploaded to the web-interface of KeyPathwayMiner and further processed and analysed in the cytoscape app. Hubs were selected based on the highest betweenness centrality value.

### Data availability

All data is deposited and can be post-analyzed online at “msatlas.dk”. Raw data are available upon special request and will be also publicly available in GEO (ID GSE138614). The analysis script is in Additional file 2.

## Results

### Comparison of the WM transcriptome between MS and control

**Fig. 2:**
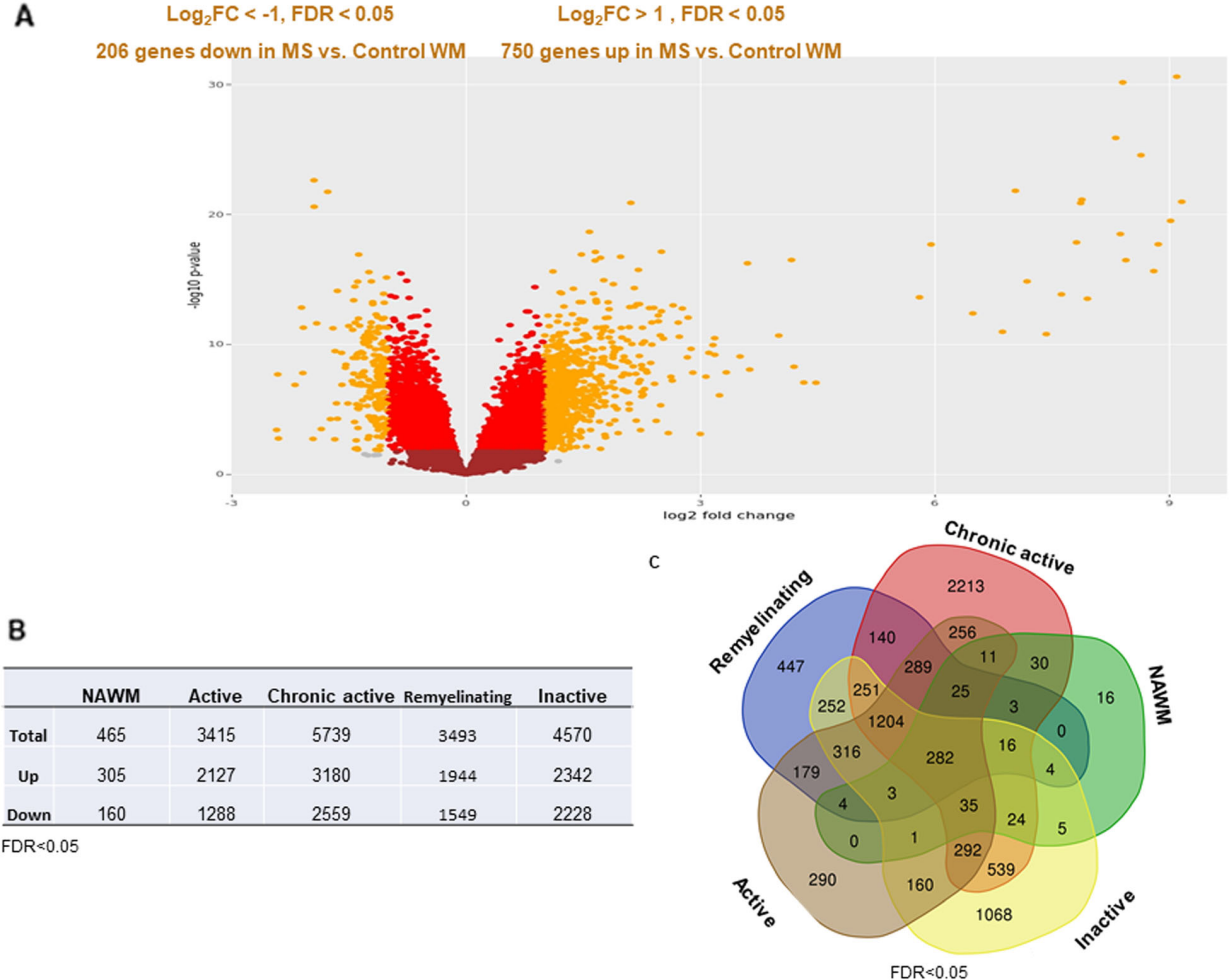
Change in gene expression profile during the evolution and fate of WM lesions in progressive MS. **a.** Visualization of the transcriptional landscape of genes (*n* = 18,722) detected between MS and non-MS (dots in graph); DEGs are indicated in bright red and orange, where orange indicates log_2_FC > 1 or < − 1. **b.** Total number of up- and downregulated DEGs when comparing each lesion type or NAWM to control WM. **c.** The Venn diagram represents the number of overlapping and lesion-specific DEGs between WM lesion types (active, inactive, remyelinating, chronic active) and NAWM compared to control WM tissue. FDR: false discovery rate; FC: fold change; WM: white matter; NAWM: normal-appearing WM; DEGs: differentially expressed genes (FDR < 0.05)

First, we compared the transcriptome of the global MS tissue (NAWM and lesions) to control WM tissue: out of 18,609 detected genes, 6713 were DEGs (FDR < 0.05 compared to control WM) (Additional file 5: Table S2 and Fig.2A). More than 3000 DEGs were detected for each lesion type, respectively. In the NAWM, only 465 DEGs were present, and the highest number of DEGs was found in chronic active lesions (Fig. 2b). More DEGs with fold change in expression level (log_2_FC > 1/<− 1, FDR < 0.05) were upregulated (*n* = 750) than downregulated (*n* = 206) in the global MS WM transcriptome landscape (Fig. 2a).

To identify common and uniquely expressed genes, we compared DEGs between each lesion type (Fig. 2c). We identified 282 common DEGs: among them genes encoding for proinflammatory cytokines, chemokines and complement factors (e.g. *IL16, TNFDF14, TNFAIP8, TNFRSF10A, CXCL12, C7, CFH, CFI*). In contrast, the number of unique lesion specific DEGs was much higher, 4034 (Fig. 2c**,** Additional file 6: Table S3).

**Fig. 3:**
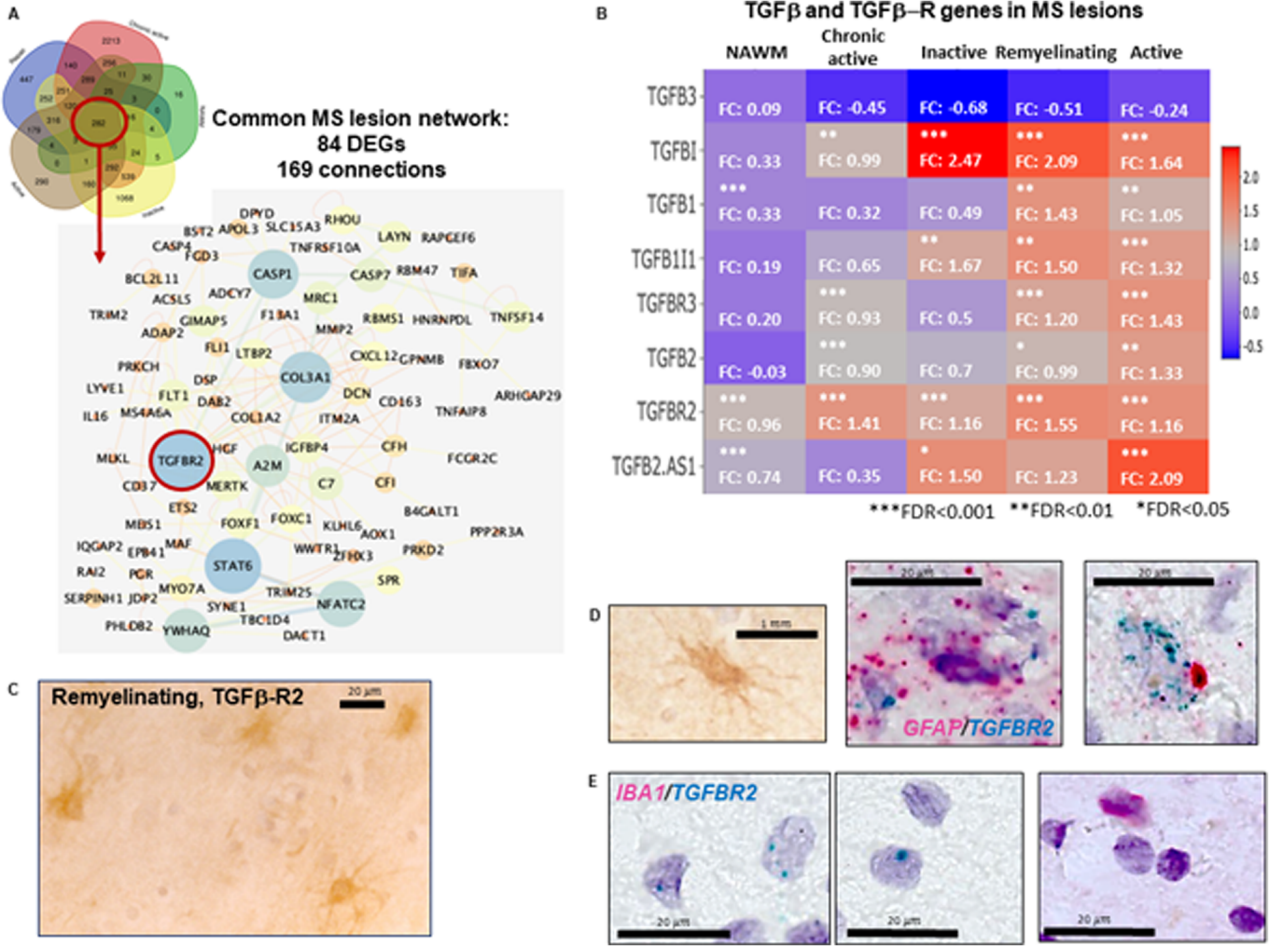
TGFβ-R2 as a major hub in the common MS lesion-specific de novo network. **a.** *De-novo* enriched protein-protein network of the 282 DEGs that were expressed in all MS WM tissue 84 nodes, 169 protein connections). **b.** Genes of TGFβ receptors and ligands in different lesion types. Asterisks indicate significant changes (*FDR < 0.05, **FDR < 0.01, ***FDR < 0.001). The number indicate the fold change (FC). **c.** TGFβ-R2 protein expression in remyelinating lesions by immunohistochemistry. **d.** *TGFBR2* (green) RNA co-localizes with *GFAP* (red) RNA in remyelinating lesions by RNAscope. **e.** *TGFBR2* (green) RNA do not co-localize with *IBA1* RNA by RNAscope in remyelinating lesions. FDR: false discovery rate; DEGs: differentially expressed genes (FDR < 0.05)

### The common MS lesion-specific de novo network and TGFβ-R2 as a major hub

We extracted the identified 282 common lesion DEGs (Fig. 2c) and examined their de novo enriched network based on protein-protein interactions (Fig. 3a). The biggest de novo network contained 84 proteins of DEGs with 169 connections. The biggest central hub based on betweenness centrality was *TGFΒR2* beside other major hubs of *STAT6, COL3A1, CASP1, NFATC2, YWHAQ, A2M* and *CASP7* (Fig. 3a). Beside TGFBR2, five out of six ligands and one additional receptor were also significant DEGs in at least one lesion type (Fig. 3b). To check cellular source, we stained for TGFβ-R2 in remyelinating lesions that had the highest expression level (Log_2_FC = 1.55, FDR = 0.0001), and the cell morphology of positive cells indicated astrocytes (Fig. 3c). By using RNAscope, we found *GFAP* and *TGFBR2* mRNA co-expressed in remyelinating lesions (Fig. 3d). Microglia did not express *TGFBR2* in this lesion type, as *IBA1* and *TGFBR2* were expressed in different cells far from each other (Fig. 3e).

Additional hubs in the de novo enriched network of shared DEGs were also linked to inflammation, such as *STAT6* (IL4 induced transcription factor), *CASP1* (part of the inflammasome) and *NFAC2* (nuclear factor of activated T cells). The major impact of inflammation as a common mechanism behind lesions was also supported by connected DEGs in the network, e.g. *IL16, CXCL12, MERTK, CASP4, C7, CD37*, or *CASP7* (Fig. 3a).

### Transcriptome changes among lesion types

**Fig. 4:**
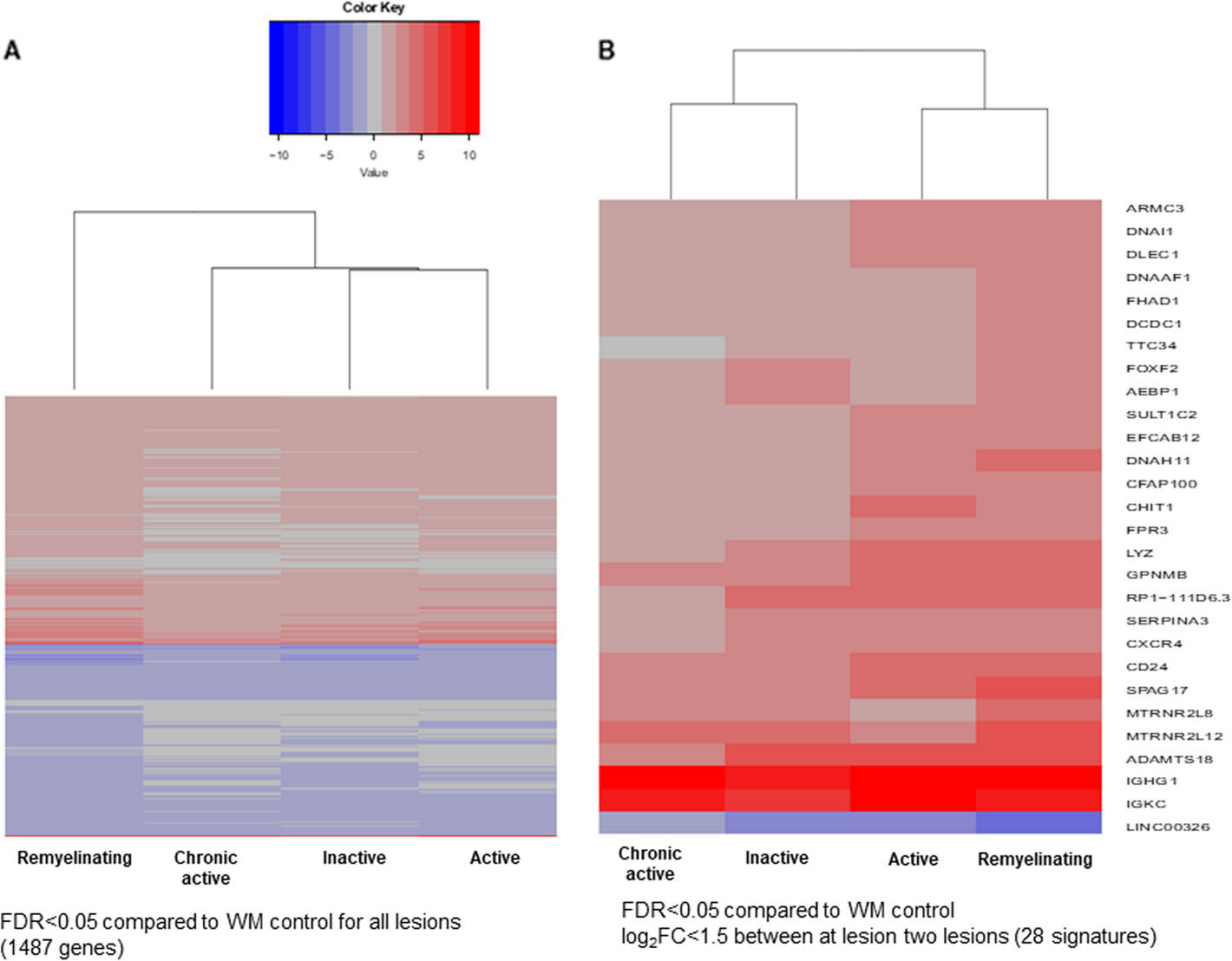
Transcriptome changes among lesions in progressive MS. **a.** Heatmap showing the 1487 DEGs in all WM lesion types compared to WM. **b.** Heatmap showing 28 DEGs in different WM lesion types compared to control WM with a selection of the DEGs with highest fold changes (log_2_FC > 1.5 for at least one pair of lesions types). FC: fold change; FDR: false discovery rate; WM: white matter; NAWM: normal appearing WM; DEGs: differentially expressed genes (FDR < 0.05)

To identify transcriptome changes and generate the molecular signatures of different WM lesion types in progressive MS, we first selected the DEGs for all lesion types compared to control (FDR < 0.05) (Fig. 4a). For each of these selected DEGs, we then calculated the fold-changes between the different lesions types and chose those that were > 2 times (1.5 < log_2_FC) differentially regulated between at least two lesion types for generation of heatmaps (Fig. 4b). By applying the fold change threshold between lesion types, the heatmap consisted of 28 DEGs, where the Long Intergenic Non-Protein Coding RNA 326 (*LINC00326*) was the only DEG that was downregulated (Fig. 4b). Remyelinating lesions had the most upregulated DEGs in both analyses. It differed the most from all the other lesion types, when comparing all the DEGs (Fig. 4a). A few genes were much higher expressed in remyelinating lesions compared to the rest; either involved in axonal assembly (*SPAG17, DNAH11, DCDC1 DNAAF1)* or unknown *(FHAD1, TTC34)*. The metabolic gene *(ADAMST18),* chemokine receptor *(CXCR4),* plasma protease inhibitor (*SERPNA4)* and lincRNA *(RP1-111D6.3)* were particularly less upregulated in chronic active lesions (Fig. 4b). The pattern of DEGs were more similar between active and remyelinating lesions, while chronic active DEGs pattern resembled more to inactive lesions (Fig. 4b).

### Cellular processes and cellular locations of lesion specific DEGs

To examine molecular processes of unique DEGs for each lesion type, we used GOrilla. We uploaded the unique DEGs (290 for active, 447 for remyelinating, 1068 for inactive, and 2213 for chronic active, Additional file 6: Table S3) to Gorilla, and extracted pathways (0 for remyelinating, 3 for active, 2 for chronic active, 11 for inactive) and molecular functions (0 for remyelinating and active, 24 for chronic active, 13 for inactive) (Additional file 7: Table S4).). In active lesions, the three identified pathways were “Immune System”, “Innate Immune System” and “Neutrophil degranulation”. For chronic active lesions, the two pathways identified were the “Histamine H1 receptor signaling” and the “Wnt signaling pathway”; the 11 identified pathways in inactive lesion were mainly related to cellular responses to stress and heat shock proteins, metabolism and the “Neutrophil degranulation” (Additional file 7: Table S4).

**Fig. 5:**
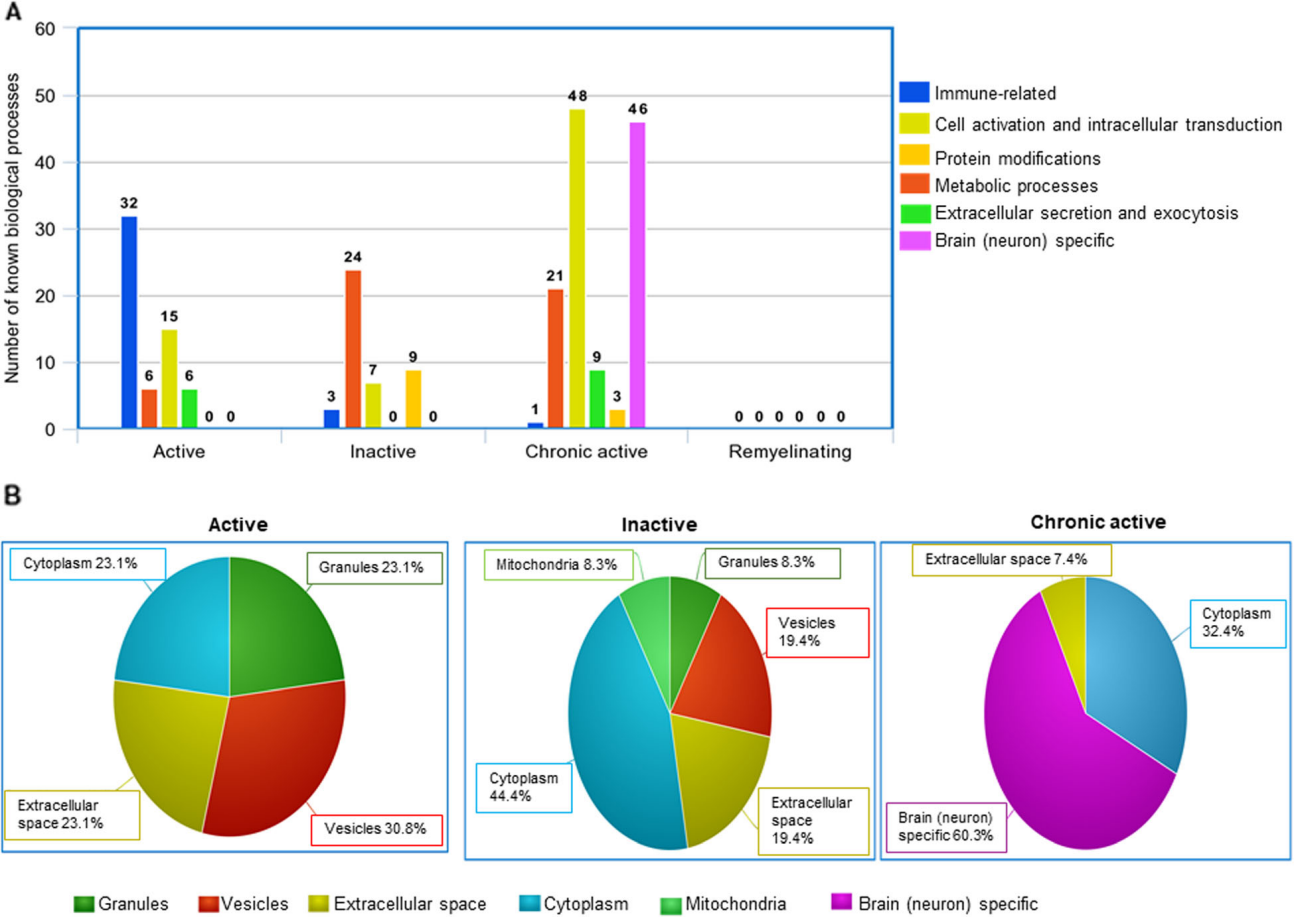
Lesion/specific biological processes and cellular locations. **a.** The bar chart shows the number of biological processes in the different lesion types (active, inactive, chronic active, remyelinating) based on the lesion-specific DEGs. We have classified them in six groups: “immune-related”, “cell activation and extracellular transduction”, “protein modifications”, “metabolic processes”, “extracellular secretion and exocytosis”, and “brain (neuron) specific”. **b.** The circle diagram for each lesion type (active, inactive, chronic active) represents the percentage of cellular components identified from the lesion-specific DEGs and grouped in “granules”, “vesicles”, “extracellular space”, “cytoplasm”, “mitochondria” and “brain (neuron) specific”. No predefined cellular component was predicted for remyelination. FDR: false discovery rate; DEGs: differentially expressed genes (FDR < 0.05)

We classified the biological processes in six groups: “immune-related”, “cell activation and extracellular transduction”, “protein modifications”, “metabolic processes”, “extracellular secretion and exocytosis”, and “brain (neuron) specific” (Fig. 5a). Active lesions were mostly enriched in immune-related biological processes (54%); inactive lesions were enriched in metabolic processes (56%); and chronic active lesions were highly enriched in both cell activation/intracellular transduction signaling and brain (neuron) specific biological processes (73%). No specific known biological process was identified in remyelinating lesion.

We grouped the cellular components identified from the lesion-specific DEGs in “granules”, “vesicles”, “extracellular space”, “cytoplasm”, “mitochondria” and “brain (neuron) specific” (Fig. 5b). In active lesions, the DEGs of cellular components were related to vesicles, extracellular space, cytoplasm and granules. In inactive lesions, cytoplasm was the dominant area of localization besides mitochondria, granules, vesicles, and extracellular space. In chronic active lesions, 60% of the cellular components were brain/neuron specific, and the rest of unique DEGs were related to cytoplasm and extracellular space. No specific known cellular components were identified in remyelinating lesions.

### Unique de novo protein-protein networks of different lesion types

**Fig. 6:**
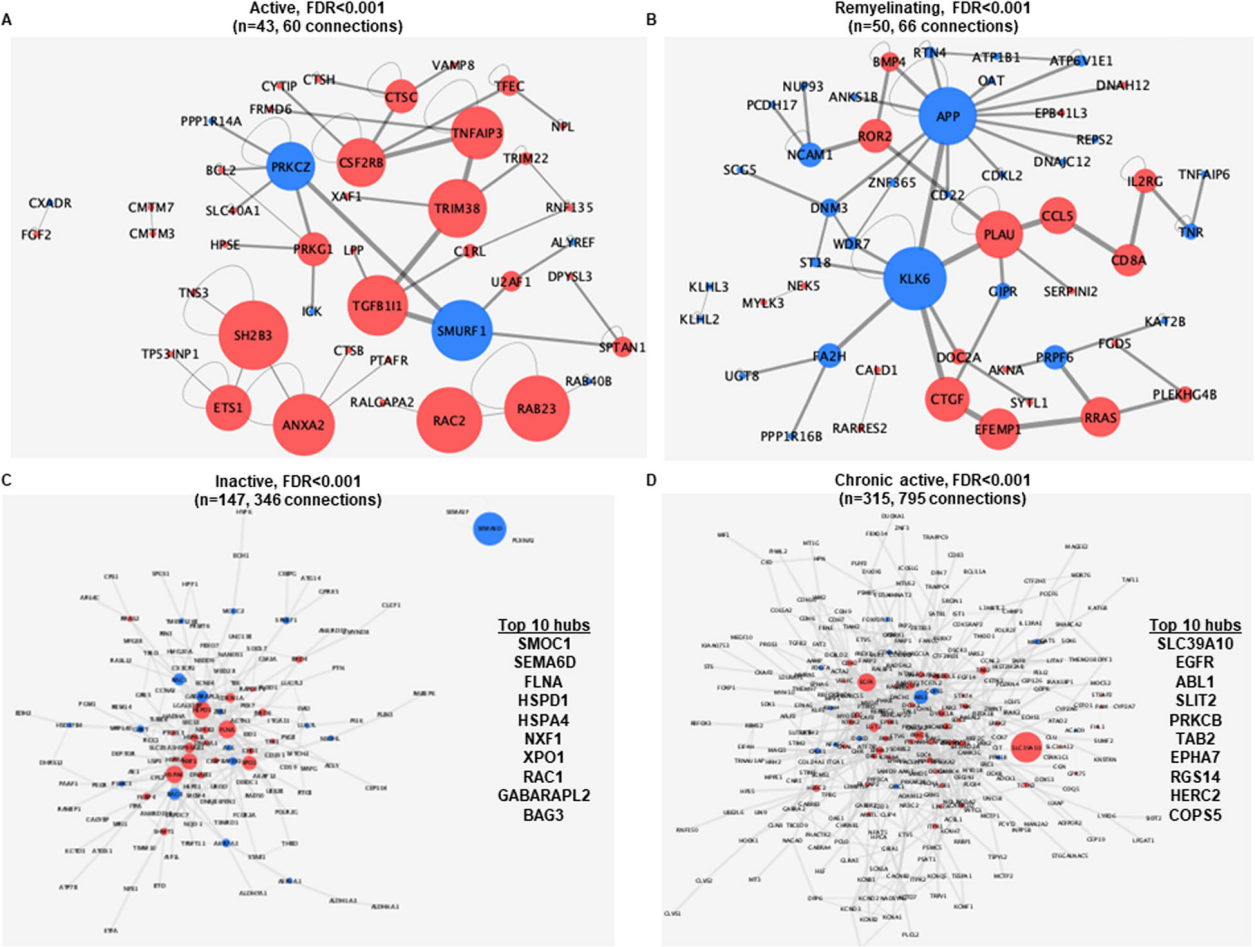
Lesion specific signatures and their biggest de novo network enrichment. By using KeyPathwayMiner, we retrieved the biggest de novo brain-specific protein-protein network from each of the lesion-specific gene list ((FDR < 0.001: active = 164, remyelinating = 235, inactive = 484, chronic active = 853) without any exception nodes. **a**. Forty-three nodes with 60 connections in active lesions. **b**. Fifty nodes with 66 connections in remyelinating lesions. **c.** One hundred forty-seven nodes with 346 connections in inactive lesions. **d**. One hundred fifteen nodes with 795 connections in chronic active lesions. Red color indicates upregulation, while blue color indicates downregulation of the nodes. The size of the node indicates higher betweenness centrality. FDR: false discovery rate

Beside the known predetermined pathways, we examined de novo pathways based on the lesion stage-specific gene expression. By using KeyPathwayMiner, we mapped each of the highly significant lesion-specific DEGs (FDR < 0.001: active = 164, remyelinating = 235, inactive = 484, chronic active = 853) (Fig. 6**)** to a brain-specific protein-protein network, and retrieved the biggest de novo subnetwork with hubs for each lesion type (Fig. 6). We selected the ten major hubs in each lesion type based on the magnitude of betweenness centrality. In the active lesion-specific biggest network (*n* = 43 with 60 connections), the ten major hubs were *SH2B3, RAC2, RAB23, ANXA2, SMURF1, TGFB1L1, TRIM38, TNFAIP3, CSF2RB* and *PRKCZ* reflecting processes involved in autoimmunity risk and immune responses (Fig. 6a). The remyelinating lesion-specific network contained 50 DEGs with 66 connections, and the ten major hubs were *KLK6, APP, PLAU, CTGF, EFEMP1, RRAS, CCL5 ROR2* and *CD8A* indicating ongoing adaptive immune responses and upregulated growth factor genes; additional well known immune related genes were also present in the network, such as *ILGR2, TNFs, NCAM1* (Fig. 6b). However, none of the genes of tissue-resident CD8^+^ T cells were significantly changed in this lesion (CXCR6, GPR56, CD49a, CD44, PD-1, CD103, CD69), and the genes of cytotoxic molecules granzyme B and GPR56 were not changed either (www.msatlas.dk). The inactive lesion-specific network consisted of 147 DEGs with 346 connections. The ten major hubs were *SMOC1, SEMA6D, FLNA, HSPD1, HSPA4, NXF1, XPO1, RAC1, GABARAPL2* and *BAG3* pointing to oxidative stress and protein modification (Fig. 6c). The chronic active lesion-specific network included 315 DEGs with 795 connections, and the ten major hubs were *SLC39A10, EGFR, ABL1, SLIT2, PRKCB, TAB2, EPHA7, RGS14, HERC2* and *COPS5*; in general, this lesion-specific network was focused on neuronal and axonal changes (Fig. 6d).

### Transcriptome signature of the NAWM and CD26 a lesion marker in diverse cell types

**Fig. 7:**
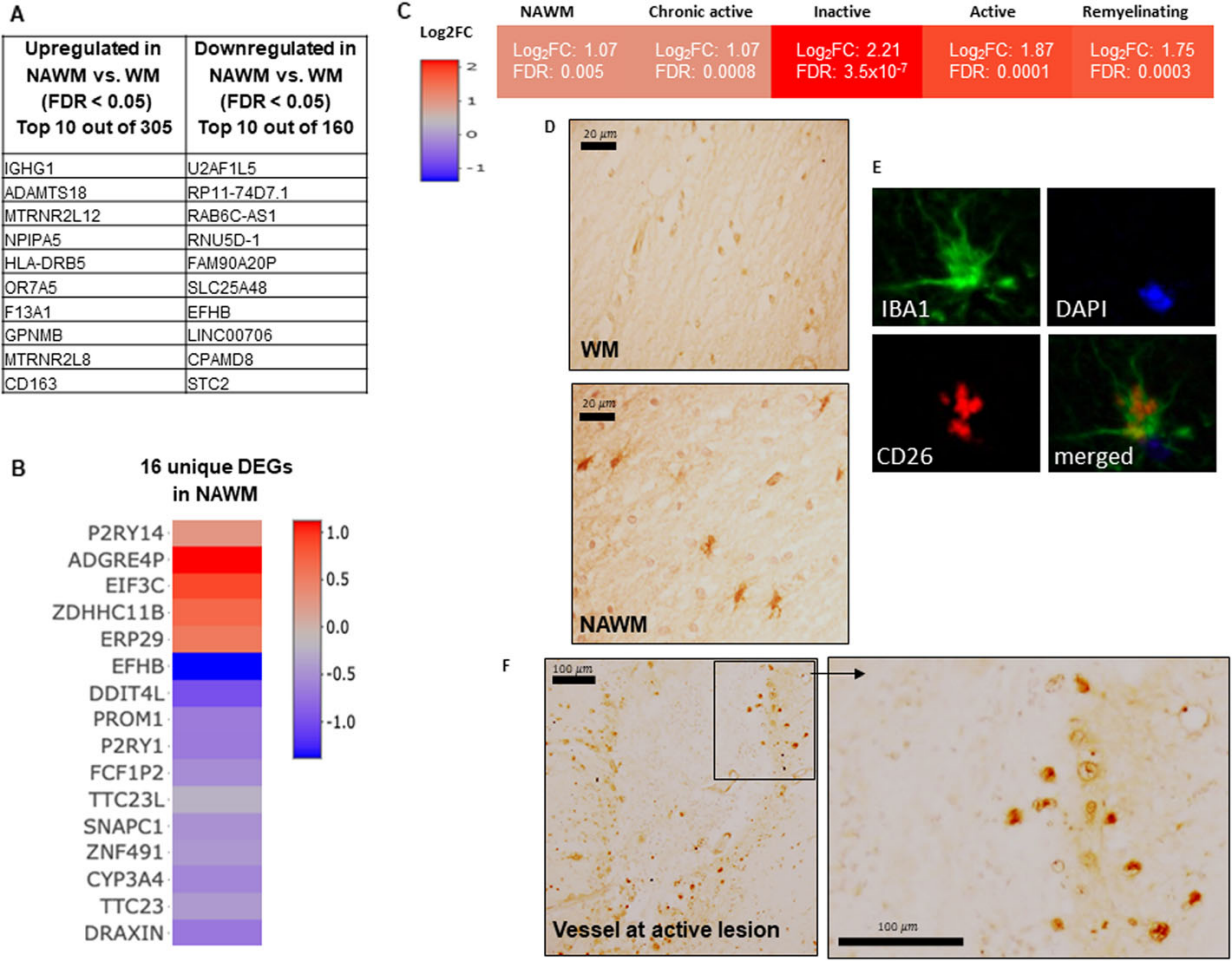
Molecular signature of NAWM and CD26/DPP4 expression. **a.** The top ten up- and downregulated DEGs in NAWM compared to control WM. **b.** The 16 uniquely expressed DEGs in NAWM. **c.** The RNA expression level of *CD26/DPP4* in the NAWM, chronic active remyelinating, active and inactive lesions. **d.** The protein expression of *CD26/DPP4* in the NAWM and absence in control WM. **e.** The co-localization of CD26/DPP4 with IBA1 in the NAWM. **f.** Expression of CD26/DPP4 at a vessel in an active lesion. FDR: false discovery rate; DEGs: differentially expressed genes (FDR < 0.05); WM: white matter; NAWM: normal appearing WM

To address changes before the evolution of lesions, we examined all DEGs (*n* = 465) in NAWM. We detected 305 upregulated and 160 downregulated DEGs (Fig. 2b). Of the ten upregulated genes, microglia/macrophages/immune related DEGs (*GPNMB, CD163, HLA-DRB5, F13A1, IGHG1*), mitochondrial humanins (*MTRNR2L8, MTRNR2L12*) and brain specific genes (*POR7A5, NPIPA5*) were present. The downregulated DEGs were not related to specific cell types or pathogenic mechanisms (*U2AF1L5, SLC25A48, CAMPD8)*, or belonged to the long-intronic-non-coding (linc) RNA class *(LNC0706, RNU5D-1, RAB6C-AS1, RP1-74D7.1*) (Fig. 7a). The 16 unique NAWM DEGs (Figs. 2c and 7b) also belonged to unknown/unspecific functional group (ERP29, TTC23) or non-protein-coding RNAs (SNAPc1, RNU5D-1, FCF1P2) besides regulation of proliferation (EIF3C, PROM1/CD133, DDIT4L), metabolic (CYP3A4, P2RY1), axonal specific (DRAXIN), and cellular interaction (ADGRE4P) DEGs.

Among the common DEGs, we found *CD26/DPP4* encoding for dipeptidylpeptidase 4 that was also identified in the NAWM in a previous study by RNA-seq and DNA methylation analysis [36] (Fig. 7c). We confirmed the protein expression of CD26 in the NAWM, and its absence in control WM by immunohistochemistry (Fig. 7d). The morphology of cells expressing CD26 in NAWM indicated microglia, and CD26 co-localized with IBA1 (Fig. 7e). In the active lesions, CD26 was expressed by mononuclear cells in the vicinity of vessels rather than by microglia (Fig. 7f).

### Immunoglobulin signatures in the different WM lesion types

**Fig. 8:**
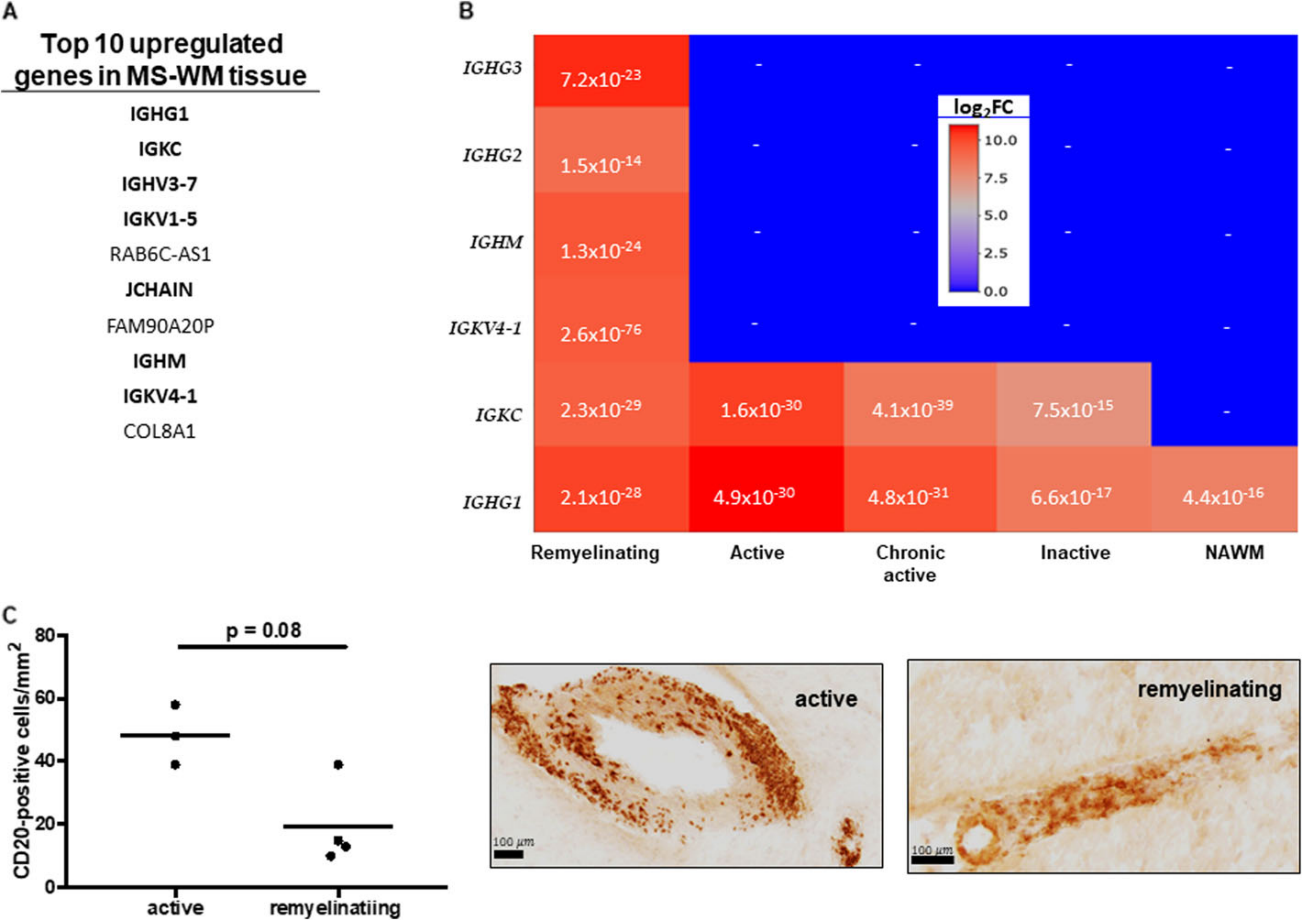
Immunoglobulin signatures and B cells in WM lesions of patients with progressive MS. **a.** Immunoglobulin transcripts among the top 10 upregulated DEGs in the global MS WM tissue compared to control WM tissue. **b.** Heatmap of immunoglobulin transcripts in the different lesion types and NAWM. **c.** Number of CD20^+^ cells in active (*n* = 3) and remyelinating (*n* = 4) lesions, each lesion from different brains. The same brains and lesions were used for generating transcriptome signatures. Around 10 pictures per lesion in each patient were taken at magnification of 20x and the average cell number per lesion were calculated. Statistical difference between the CD20^+^ cells was calculated using the Mann-Whitney test. Immunohistochemistry shows CD20^+^ cells at small vessels within an active and remyelinating MS lesion, respectively. FDR: false discovery rate; DEGs: differentially expressed genes (FDR < 0.05); WM: white matter; NAWM: normal appearing WM

We noticed that immunoglobulin genes were present among the top 10 upregulated DEGs in the global WM tissue of MS (Fig. 8a). To examine their distribution in the different lesion types, we produced a heatmap with all significant (FDR < 0.05) immunoglobulin transcripts (Fig. 8b). *IGHG1* encoding for heavy chain IgG1was highly expressed in all lesions and in the NAWM; *IGKC* encoding for the constant region of light kappa chain was highly expressed in all lesions; genes encoding for IgG2, IgG3 and IgM heavy chains (*IGHG2*, *IGHG3*, *IGHM*), and variable region of light kappa chain (*IGKV4–1*) were only present in remyelinating lesions. To examine, if such high expression of immunoglobulin genes was due to a higher number of B cells, we investigated the presence of B cells by quantifying CD20^+^ cells in active (*n* = 3) and remyelinating (*n* = 4) lesions each from different patients (*n* = 7). We found that CD20^+^ cells were mostly present in active lesions, but remyelinating lesions had the most heterogenous upregulated transcripts (Fig. 8c).

## Discussion

We introduce the first mechanistic investigation of transcriptome signatures of lesion evolution and fate in the WM of patients with progressive MS across all major WM lesion types: NAWM, active, inactive, chronic active and remyelinating lesions (compared to control WM). One study applied next generation RNA sequencing to examine gene expression in the NAWM [37], and a very recent work examined oligodendrocyte nuclei signatures in MS lesions [39]. We controlled for confounders using generalized mixed effect linear models considering age, sex and multiple samples of the same patient. We corrected all results for multiple testing with a target FDR value < 0.05 to use a conservative statistical estimation of gene expression changes. We detected a high number of differently expressed genes (DEGs) in different lesion types (compared to the control samples) using an FDR-corrected *p*-value threshold of 0.05. Most of these DEGs with high fold change were upregulated in MS tissue. We then, for the first time, interrogated the human interactome for sub-networks that putatively drive MS lesion evolution mechanistically.

### Common DEGs in different lesion types

The central hub in the *de novo* network based on shared DEGs was TGFβ-R2 that was most upregulated in remyelinating lesions (Fig 3). By immunohistochemistry and RNAscope, we found that TGFβ-R2 was expressed by astrocytes in remyelinating lesions. The gene expression of one of its major ligands, TGFβ1 was significantly expressed in active and remyelinating lesions only, in contrast to genes of TGFβ2 and TGFβ3. TGFβ1 has been associated with reparatory function in the CNS [15]. A recent study on microarray data from spinal cord periplaque *vs*. NAWM identified TGFβ1 in the context of astrocytosis and remodeling [64]. Astrocyte targeted overexpression of TGFβ1 resulted in earlier and more severe experimental autoimmune encephalomyelitis [57, 89], while systemic administration inhibited disease [47]. A previous study suggested that TGFβ activity leads to the formation of chronic MS lesions [13]. Indeed, we found that *TGFΒ2* was a DEG in chronic active lesions in addition to active and remyelinating ones. But it may have a dual role in by both regulating the immune responses and promoting neuronal survival [27], and is an important player of neural stem/precursor cells (NPCs) immunomodulation [12]. Since we observed unique neuron/axon specific activities in chronic active lesions (Fig. 5), the highly significant expression of *TGFΒ2* may be also related to this. Our data indicates that the effect of TGFβ ligands and their receptors in the MS-CNS may depend on the lesion types with different inflammatory and cellular environment [15].

### Transcriptome changes specific to different lesion types

In order to investigate unique transcriptional changes at different stages of lesion evolution and fate, we applied a comprehensive approach: (i) we identified DEGs that were differentially regulated at least among two lesion types visualized in a global transcriptome heatmap (Fig. 4); (ii) we investigated predefined pathways based on DEGs that were present only in one lesion type (Fig. 5); (iii) we extracted unique significant (FDR < 0.001) up- and downregulated genes in each lesion type, and created de novo enriched protein interaction networks with major hubs for these DEGs (Fig. 6).

#### Active lesions

Based on all the different analyses, the molecular signature of active lesions indicated the classic inflammatory MS lesion governed by immune responses (Figs. 5 and 6, Additional file 6: Table S3): over 50% of the biological processes were immune-related and were distributed to granules, vesicles, extracellular space and the cytoplasm, suggesting extracellular effects on the microenvironment. The ten major hubs in the de novo network enrichment analysis have all been related to MS or autoimmune/brain diseases (Fig. 6): MS susceptibility genes (*SH2B3* [3], *RAB23* [50]), potential biomarkers (*CSF2RB* [70], *ANXA2* [38]), or potential roles in inflammatory/brain diseases or MS pathogenesis (*RAC2* [78], *Smurf1* [62], *TGFB1I1* [53], *TRIM38* [35], *TNFAIP3* [33], *PRKCZ* [51]). Active lesions exhibited the least unique DEGs (*n* = 290, FDR < 0.05), which could suggest that active lesion is the primary step for lesion evolution, and the source of all other lesion types.

#### Remyelinating lesions

The signature of active lesions was more similar to remyelinating lesions than to inactive and chronic active lesions, and remyelinating lesions had the second least unique DEGs (*n* = 447, FDR < 0.05). Remyelinating lesion type differed the most from all the others in the context of DEGs present in all lesion types (Fig. 4a). However, the most different molecular signature was found between chronic active and remyelinating lesions when setting a threshold between the difference in expression level between at least two lesion types (Fig. 4b). We did not detect any known predefined pathways, biological processes, cellular component enrichments or molecular function with Gorilla, WeGestalt or FunEnrich in remyelinating lesions. That contrasted with the highest expression level of shared lesion DEGs (Fig. 4b). Some of these were related to dynein-dependent axonal transport during brain development (*Spag17, Dnah11, DNAAF1, DCDC1*), which could suggest neuronal response to stress [84].

In the de novo network enrichment analysis of unique DEGs, well known immune-related genes were present such as *TNF, CCL5, NCAM1*, *PLAU*, *CD8* and *ILGR2*. This may indicate an overlap between active and remyelinating lesions, and may be related to early remyelinating events also indicated by partial remyelination of these lesions. The genes for molecules characterizing tissue-resident CD8^+^ cells (Trm cells) were not significantly changed, and the genes for cytotoxic molecules granzyme B and GPR56 were also not upregulated.

Unique downregulation of two hubs supported protective events: *KLK6*, which has been indicated as a marker for disease worsening in EAE and MS [5], and *FA2H* that has been linked to WM neurodegeneration [45]. Two additional upregulated hubs supported regenerative processes: *CTGF*, a central mediator of tissue remodeling [52] and *EFEMP1* promoter of cell growth often implicated in cancer [91]. However, *CTGF*, has also been found to inhibit myelination [21]. Despite the observed remyelination, myelin genes (*MBP, MOG, MAG*) were not upregulated. This was characteristic of all lesion types (*data not shown*) similar to recent data by microarray [92]. A recent work that examined oligodendrocyte heterogeneity by single nuclei sequencing and found that there was a reduction of OPCs in MS and NAWM compared to control, and the subclusters of mature oligodendrocytes were skewed between MS and control tissue [40]. The number of OPCs and oligodendrocytes are reduced in MS lesions [7, 39, 55], which may also be responsible for the observed absence of changes in myelin gene expression.

#### Chronic active lesions

In contrast to remyelinating lesions, chronic active lesions were characterized by a high number of differentially regulated predefined biological processes; the highest number of DEGs (*n* = 5739) and the highest number of unique DEGs (*n* = 2213). This distinctive nature of chronic active lesions was also reflected by the biggest de novo network of unique DEGs. The heatmap also suggested that chronic active lesions were the most different from remyelinating lesions (Fig. 4b).

We observed a dominance of neuron/synapse specific biological processes in white matter lesions (Fig. 5**,** Additional file 7: Table S4) that was somewhat unexpected. Recent evidence has localized mRNA translation in subcellular regions of neurons (dendrites, axons, synapses, somas) [76]. It is also well known that OPCs do express many synaptic markers and are shown to form synapses in the WM as well [28]. It may be RNA in vesicles transported through the axons or maybe some genes that also play a role in glia cells. In contrast to active lesions, intracellular transduction events were more common, such as binding reactions with metal ions, anions, ATP, and cytoskeleton proteins (Additional file 7: Table S4). Synaptic and axonal events were also supported by the increased enrichment of lesion-specific DEGs belonging to the cadherin (CDH) family, potassium family (KCN and KCTD), the ephrin (ECH) receptors and GABA genes (Additional file 6: Table S3). Five of the ten biggest hubs in the de novo network enrichment analysis were all also neuron-related: *SLC39A10* [63], *SLIT2* [88], *EPHA7* [10], *RGS14* [79], *HERC2* [11], *COPS5* [87]. DEGs suggested lower inflammatory response in chronic active lesions compared to the other lesion types. The only identified immune related process was” response to wound healing”, which could suggest a more post-inflammatory reaction (Additional file 7: Table S4). One of the major hubs, SLIT2, mentioned above, is released from neurons and also inhibits leukocyte chemotaxis migration [88].

Altogether, such neuron/synaptic gene activity in chronic active lesions indicates intrinsic irreversible pathogenic events less coupled with inflammatory reactions, and could also explain why immune-related treatments work less in progressive MS, where this lesion type increases [56].

#### Inactive lesions

Inactive lesions had over twice as many unique DEGs as active lesions (*n* = 1068). These suggested protein modifications, cellular stress, heat shock protein responses, metabolic events, catabolic and “breaking down” of components (Fig. 5**,** Additional file 6: Table S3, Additional file 7: Table S4). This was further supported by major hubs in the de novo network enrichment analysis (*HSPD1, HSPA4*) (Fig. 6). Two nuclei exporter proteins (NXF1, XPO1) were also among the ten major hubs, and they are known to be upregulated after neuronal damage to prevent repeated neurotoxicity [30, 43, 82]. The export, folding/unfolding of proteins plus the catalytic and oxidoreductase reactions takes place in the cytoplasm, which was also predicted as the dominant cellular location (Fig. 5b) including different organelle compartments, vesicles and mitochondria (Additional file 7: Table S4).

### NAWM and Dipeptidylpeptidase IV (CD26/DPP4)

We found 465 DEGs in NAWM (Figs. 2 and 7). Most of the upregulated genes were microglia/macrophage/immune related, which may suggest an altered phenotype of diffusely activated microglia throughout the NAWM as indicated recently [85]. Mitochondrial and brain specific genes were also altered, suggesting other intrinsic events either before the development of active lesions or indicating changes as consequence of pathological events and lesion development at other sites. Mitochondrial humanins (*MTRNR2L12, MTRNR2L8*) that may protect cells from oxidative stress [90] were significantly upregulated in both NAWM and all lesions.

The lower number of DEGs also contrasted the thousands of DEGs in lesions: while NAWM differed from control WM only by 465 DEGs, it differed from lesions by 3894 DEGs. The unique-specific DEGs in NAWM were enriched by unspecified or non-protein-coding genes, which may suggest that more unconventional approaches may be needed to understand mechanistic changes before lesion evolution.

We also found *CD26* in the NAWM and all lesion types (Fig. 7). A recent study also detected significant expression of *CD26* in both DNA methylation and RNA seq data in the NAWM tissue [37]. CD26/DPP4 is a membrane-associated exopeptidase that by engaging inhibitory ligands may limit autoimmunity in mice by regulating Th1 responses [68, 80], and by hydrolyzing substrates CXCL12 and CCL5 [8]. By using immunohistochemistry and immunofluorescence, we found that CD26 was expressed by microglia in the NAWM. In contrast, in active lesions, the CD26^+^ cells had mononuclear morphology.. These data suggested that CD26 may be related to an altered microglia function/phenotype in the NAWM and continue to be significantly expressed in lesion types. The recent report of protection against cuprizone-induced demyelination by an inhibitory ligand of CD26 [18] also suggests regulation of microglia function, since the role of T cells in this model is probably minor [32, 71]. The different cellular source of CD26 in NAWM and active lesions also indicate that differential regulation of a gene should be addressed in the context of lesion type and cellular source to interpret potentially different functional outcomes.

### Immunoglobulins and B cells

We noticed that immunoglobulin genes were among the top 10 upregulated genes in WM MS tissue vs. control WM (Fig. 8). The highly significant expression of immunoglobulin genes among the total MS-WM can be explained by presence of plasma cells, or by increased transcription of rearranged B cell receptors secreted also as antibodies. There is an increasing recognition of B cells and plasma cells also in the WM lesions of PMS besides the B cell-rich aggregates [57, 61, 79, 80]. Here, we found B cells in WM lesions mostly located around the vessels. All lesions had *IGHG1* and *IGKC* highly upregulated. A proteomic study of CSF also found the protein of Ig gamma-1 chain (*IGHG1*) more abundant in fulminant MS samples compared to control [26]. Another study also confirmed the expression of *IGKC* and *IGHG1* in NAGM and GM lesions with both microarray and qPCR [83]. The transcribed immunoglobulin genes we detected, may be secreted because among the top 10 upregulated is *J-CHAIN*, which serve to link immunoglobulins in dimer (IgA) or pentamer (IgM) as secretory components [40]. The dominance of immunoglobulin genes among the top upregulated DEGs was disproportional to the number of B cells (Fig. 8), indicating either a restricted B cell clonality, or high secretion of immunoglobulins.

*IGKV4–1, IGHM, IGHG2, IGHG3* were uniquely upregulated in remyelinating lesions. The *IGHV4* transcript was also most frequently found in B cell receptor transcriptome of the CSF and paired brain-draining lymph node tissue [41, 66, 81], and maybe related to rare T cell exposed motifs [34]. The specific presence of the variable regions in the remyelinating lesions may indicate a more heterogeneous B cell phenotype with paratopes to a wider range of epitopes. The heterogenous upregulated transcripts for immunoglobulins of other groups (*IGHG2, IGHG3)* in remyelinating lesions could be due to another minor overlooked mature B cell subpopulation. The presence of B cells in remyelinating lesions were also emphasized by de novo pathway analysis where both T- and B cell markers were present (Fig. 6). A recent work also emphasized the presence of plasma cells in lesions of patients with progressive MS [58]. Altogether, these data argue for important role of B cells even in the WM of progressive MS. Whether the heterogeneous immunoglobulin genes in remyelinating lesions could reflect some special subset of B cells is not clear; we were not able to detect *IL10* transcripts in remyelinating lesions that may be related to regulatory B cells [77], but we did find the IL10 receptor (*IL10RA)* highly upregulated in the lesions, mostly in remyelinating ones (log_2_FC 2,3 and FDR 4.2 × 10^− 5^).

## Conclusion

In conclusion, by next-generation RNA sequencing and a comprehensive computational systems medicine approach, we identified the first mechanistic transcriptome signature of lesion evolution and fate in the progressive MS brain WM.

The major de novo network of shared DEGs among the different lesions emphasize inflammation as a common mechanism, and support the view also provided by GWAS that MS is an inflammatory disease, even at the later progressive stage.

We found that each lesion type had a huge complexity of molecular pathways, and although we tried to categorize them to simplify for better understanding, many pathways were unexpected and overlapping indicating a dynamic process of lesion evolution. TGFβR2 was major hub of shared DEGs by all lesions and NAWM, and it was expressed by astrocytes in remyelinating lesions. Our data also suggest that chronic active lesions that are more frequent during the late progressive phase have more complex molecular mechanisms and changes in multiple pathways. This lesion type was profoundly different from all other lesion types, and also from control WM. NAWM was more similar to control WM than to any other lesion types. This indicates that major gene expression changes occur both at early lesion genesis, and in lesions most characteristic as the late progressive phase develops. Besides unique sub-networks mechanistically evolving different lesions stages, some molecules were specifically regulated: CD26/DPP4 by microglia in the NAWM and by mononuclear cells in active lesions. The uniqueness of lesion types also indicates that omics approaches should consider lesion stages, when expression and regulation of different molecules are addressed. Although this study indicates the extreme diverse molecular events on transcriptome level at different lesion stages, our comprehensive unbiased search across subsets of multiple lesions provided a discovery of specific molecular mechanistic signatures validated by different approaches.

A limitation of our study is the absence of controls with other neurological disease, and the lack of separation of rim and core in the chronic active and inactive lesions. Nevertheless, the combination of different bioinformatics methods and validation by immunohistochemistry supported our conclusions, and overcome these limitation for the interpretation of changes in transcriptome signatures during lesion evolution and fate in the WM of progressive MS brain.

## Supplementary information

**Additional file 1.** Supplementary Methods

**Additional file 2.** The R script for analyzing differentially expressed genes

**Additional file 3:** **Figure S1.** RNAscope : Negative (red and green targeting two bacterial genes)and postive (PPIB /POLR2A POLR2A ) controls

**Additional file 4:** **Table S1.** Clinical and demographic data of the MS patients and non-neurological disease controls

**Additional file 5:** **Table S2.** Number of samples, detected genes and significant genes in the post-mortem human brain samples

**Additional file 6:** **Table S3.** Lesion specific genes

**Additional file 7. Table S4.** Pathways of cellular processes in different lesion types based on unique differentially expressed genes.

## Data Availability

The datasets generated and/or analysed during the current study are available as interactive online database linked to bioinformatics approaches at „msatlas.dk”.
