# Supplementary material for: Molecular signature of different lesion types in the brain white matter of patients with progressive multiple sclerosis
Source: Acta Neuropathol Commun. 2019 Dec 11;7:205. doi: 10.1186/s40478-019-0855-7 (PMC6907342; doi:10.1186/s40478-019-0855-7)
Supplement: Supplementary file 1 — Additional file 1. Supplementary Methods [file 40478_2019_855_MOESM1_ESM.docx]

**Additional File 1**

**Supplementary Methods**

**Antibodies**

| **Primary antibody** | **Origin** | **Dilution** | **Source** |
| --- | --- | --- | --- |
| CD3 | Rabbit (mAB) | 1:300 | Dako |
| CD20 | Mouse (mAB) | 1:50 | Dako |
| CD26 | Mouse (mAB) | 1:75 | Abcam |
| IBA1 | Rabbit (mAB) | 1:500 | Dako |
| MHCII | Mouse (pAB) | 1:200 | Dako |
| MOG | Mouse (pAB) | 1:20 | R. Reynolds, Imperial College, UK |
| TGFBR2 | Mouse (mAB) | 1:200 | Abcam |
| GFAP | Mouse (mAB) | 1:500 | Dako |
