# Supplementary material for: Molecular signature of different lesion types in the brain white matter of patients with progressive multiple sclerosis
Source: Acta Neuropathol Commun. 2019 Dec 11;7:205. doi: 10.1186/s40478-019-0855-7 (PMC6907342; doi:10.1186/s40478-019-0855-7)
Supplement: Supplementary file 2 — Additional file 2. The R script for analyzing differentially expressed genes [file 40478_2019_855_MOESM2_ESM.docx]

**Additional File 2**

**The R script for analyzing differentially expressed genes**

The script and the differentially expressed genes are also available at GitHub:

<https://github.com/frischt/supplementary_files>

(edgeR)

library(nlme)

library(caret)

library(arm)

library(ggfortify)

library(DESeq2)

library(rgl)

library(plyr)

library(biomaRt)

library(calibrate)

################################

# load pre-processed data

################################

load("edgeRScriptData.R")

################################################################

#' FILTERING to for groups

#'

################################################################

# modify for C against ALLE

labeling_C_ALLE = as.character(labeling_1)

labeling_C_ALLE[which(labeling_1 != "WM")] = "ALL"

labeling_C_ALLE = factor(labeling_C_ALLE, levels = c("WM", "ALL"))

patientID_C_ALLE = patient_ID

pca_C_ALLE = pcaVector

age_C_ALLE = age

sex_C_ALLE = sex

# modify for C against AL

labeling_C_AL = as.character(labeling_1)

remaining_rows = which(labeling_1 == "WM" | labeling_1 == "AL")

dataMatrix = dataMatrix[,remaining_rows]

labeling_C_AL = factor(labeling_C_AL[remaining_rows], levels = c("WM", "AL"))

patientID_C_AL = patient_ID[remaining_rows]

pca_C_AL = pcaVector[remaining_rows]

age_C_AL = age[remaining_rows]

sex_C_AL = sex[remaining_rows]

# modify for C gainst NAWM

labeling_C_NAWM = as.character(labeling_1)

remaining_rows = which(labeling_1 == "WM" | labeling_1 == "NAWM")

dataMatrix = dataMatrix[,remaining_rows]

labeling_C_NAWM = factor(labeling_C_NAWM[remaining_rows], levels = c("WM", "NAWM"))

patientID_C_NAWM = patient_ID[remaining_rows]

pca_C_NAWM = pcaVector[remaining_rows]

age_C_NAWM = age[remaining_rows]

sex_C_NAWM = sex[remaining_rows]

# modify for C against RL

labeling_C_RL = as.character(labeling_1)

remaining_rows = which(labeling_1 == "WM" | labeling_1 == "RL")

dataMatrix = dataMatrix[,remaining_rows]

labeling_C_RL = factor(labeling_C_RL[remaining_rows], levels = c("WM", "RL"))

patientID_C_RL = patient_ID[remaining_rows]

pca_C_RL = pcaVector[remaining_rows]

age_C_RL = age[remaining_rows]

sex_C_RL = sex[remaining_rows]

# modify for C against IL

labeling_C_IL = as.character(labeling_1)

remaining_rows = which(labeling_1 == "WM" | labeling_1 == "IL")

dataMatrix = dataMatrix[,remaining_rows]

labeling_C_IL = factor(labeling_C_IL[remaining_rows], levels = c("WM", "IL"))

patientID_C_IL = patient_ID[remaining_rows]

pca_C_IL = pcaVector[remaining_rows]

age_C_IL = age[remaining_rows]

sex_C_IL = sex[remaining_rows]

# modify for C against CA

labeling_C_CA = as.character(labeling_1)

remaining_rows = which(labeling_1 == "WM" | labeling_1 == "CA")

dataMatrix = dataMatrix[,remaining_rows]

labeling_C_CA = factor(labeling_C_CA[remaining_rows], levels = c("WM", "CA"))

patientID_C_CA = patient_ID[remaining_rows]

pca_C_CA = pcaVector[remaining_rows]

age_C_CA = age[remaining_rows]

sex_C_CA = sex[remaining_rows]

# modify for AL vs CA

labeling_AL_CA = as.character(labeling_1)

remaining_rows = which(labeling_1 == "AL" | labeling_1 == "CA")

dataMatrix = dataMatrix[,remaining_rows]

labeling_AL_CA = factor(labeling_AL_CA[remaining_rows], levels = c("AL", "CA"))

patientID_AL_CA = patient_ID[remaining_rows]

pca_AL_CA = pcaVector[remaining_rows]

age_AL_CA = age[remaining_rows]

sex_AL_CA = sex[remaining_rows]

################################################################

#' FILTERING to for groups

#'

################################################################

#---------------------------------------------------------------

# modify for CA against all other Lesions

labeling_NAWM_LESION = as.character(labeling_1)

removing_rows = which(labeling_1 == "WM")

dataMatrix = dataMatrix[,-removing_rows]

#building the labels for the comparison

labeling_NAWM_LESION = sapply(labeling_NAWM_LESION[-removing_rows], function(x){ifelse(x == "NAWM", "NAWM", "OTHER")}, simplify = T)

labeling_NAWM_LESION = factor(labeling_NAWM_LESION, levels = c("OTHER", "NAWM"))

#building the patient ID

patientID_NAWM_LESION = patient_ID[-removing_rows]

#pca age and sex selection

pca_NAWM_LESION = pcaVector[-removing_rows]

age_NAWM_LESION = age[-removing_rows]

sex_NAWM_LESION = sex[-removing_rows]

#---------------------------------------------------------------

labeling_AL_LESION = as.character(labeling_1)

removing_rows = which(labeling_1 == "WM" | labeling_1 == "NAWM")

dataMatrix = dataMatrix[,-removing_rows]

#building the labels for the comparison

labeling_AL_LESION = sapply(labeling_AL_LESION[-removing_rows], function(x){ifelse(x == "AL", "AL", "OTHER")}, simplify = T)

labeling_AL_LESION = factor(labeling_AL_LESION, levels = c("OTHER", "AL"))

#building the patient ID

patientID_AL_LESION = patient_ID[-removing_rows]

#pca age and sex selection

pca_AL_LESION = pcaVector[-removing_rows]

age_AL_LESION = age[-removing_rows]

sex_AL_LESION = sex[-removing_rows]

#---------------------------------------------------------------

labeling_RL_LESION = as.character(labeling_1)

removing_rows = which(labeling_1 == "WM" | labeling_1 == "NAWM")

dataMatrix = dataMatrix[,-removing_rows]

#building the labels for the comparison

labeling_RL_LESION = sapply(labeling_RL_LESION[-removing_rows], function(x){ifelse(x == "RL", "RL", "OTHER")}, simplify = T)

labeling_RL_LESION = factor(labeling_RL_LESION, levels = c("OTHER", "RL"))

#building the patient ID

patientID_RL_LESION = patient_ID[-removing_rows]

#pca age and sex selection

pca_RL_LESION = pcaVector[-removing_rows]

age_RL_LESION = age[-removing_rows]

sex_RL_LESION = sex[-removing_rows]

#---------------------------------------------------------------

labeling_CA_LESION = as.character(labeling_1)

removing_rows = which(labeling_1 == "WM" | labeling_1 == "NAWM")

dataMatrix = dataMatrix[,-removing_rows]

#building the labels for the comparison

labeling_CA_LESION = sapply(labeling_CA_LESION[-removing_rows], function(x){ifelse(x == "CA", "CA", "OTHER")}, simplify = T)

labeling_CA_LESION = factor(labeling_CA_LESION, levels = c("OTHER", "CA"))

#building the patient ID

patientID_CA_LESION = patient_ID[-removing_rows]

#pca age and sex selection

pca_CA_LESION = pcaVector[-removing_rows]

age_CA_LESION = age[-removing_rows]

sex_CA_LESION = sex[-removing_rows]

#---------------------------------------------------------------

labeling_IL_LESION = as.character(labeling_1)

removing_rows = which(labeling_1 == "WM" | labeling_1 == "NAWM")

dataMatrix = dataMatrix[,-removing_rows]

#building the labels for the comparison

labeling_IL_LESION = sapply(labeling_IL_LESION[-removing_rows], function(x){ifelse(x == "IL", "IL", "OTHER")}, simplify = T)

labeling_IL_LESION = factor(labeling_IL_LESION, levels = c("OTHER", "IL"))

#building the patient ID

patientID_IL_LESION = patient_ID[-removing_rows]

#pca age and sex selection

pca_IL_LESION = pcaVector[-removing_rows]

age_IL_LESION = age[-removing_rows]

sex_IL_LESION = sex[-removing_rows]

################################################################

#' re labeling genes and building the first data matrix

################################################################

working_labeling = droplevels(labeling_RL_LESION)

working_PatientID = droplevels(patientID_RL_LESION)

working_PCA = pca_RL_LESION

working_AGE = age_RL_LESION

working_SEX = sex_RL_LESION

#working_labeling = labeling_1

################################################################

#' FILTERING

#' At least one/two counts per million mapped reads in at least

#' 5 samples, because RL with 5 is the smallest group!

#'

#' I changed this significantly? look at marks script again?!

#'

################################################################

# produce listDGE object for further processing

listDGE.dataMatrix = DGEList(counts = dataMatrix, group = working_labeling)

# check for counts based on the smallest group!

table(working_labeling)

isexpr = rowSums(cpm(listDGE.dataMatrix)>2) >= 5

sum(isexpr)

stop("PLEASE CHECK IF THIS IS CORRECT BASED ON THE GROUP SIZE")

# reduce dataset and recalculate lib size!

listDGE.reduced = listDGE.dataMatrix[isexpr, , keep.lib.sizes=F]

listDGE.reduced$samples$lib.size <- colSums(listDGE.reduced$counts)

#naming_columns = combined_matrix[isexpr,]

################################################################

#' design

################################################################

# finalDesign!

myDesign = model.matrix(~working_PCA+working_AGE+working_SEX+working_labeling)

################################################################

#'normalize in order to account for different library size

################################################################

listDGE.reduced.factors = calcNormFactors(listDGE.reduced)

listDGE.reduced.norm = estimateDisp(listDGE.reduced.factors, design = myDesign,robust = T)

listDGE.reduced.norm$common.dispersion

################################################################

#' fit linear model

################################################################

myFit = glmFit(listDGE.reduced.norm, myDesign)

lrt = glmLRT(myFit)

################################################################

#' extract top and safe them in file

################################################################

finalRes = topTags(lrt, n = nrow(lrt$genes))

print(sprintf("%d genes where significantly different between groups according to FDR (FDR<0.01)", length(which(finalRes$table$FDR < 0.01))))

print(sprintf("%d genes where significantly different between groups according to FDR (FDR<0.05)", length(which(finalRes$table$FDR < 0.05))))

#printingRes = cbind(naming_columns, finalRes$table)

write.table(finalRes, file = "OTHER_IL.txt", quote = F)

#######################

#####

#######################

geneNames = gsub(".*;", "", rownames(finalRes$table))

localRes = cbind(finalRes$table, names = geneNames)

finResTable = finalRes$table

# reload dataset

finalRes = read.table("gene_expression/type_1/WM_ALLE.txt", header = T)

finalRes = read.table("gene_expression/type_1/WM_CA.txt", header = T)

finalRes = read.table("gene_expression/type_1/WM_IL.txt", header = T)

finalRes = read.table("gene_expression/type_1/WM_NAWM.txt", header = T)

finalRes = read.table("gene_expression/type_1/WM_RL.txt", header = T)

geneNames = gsub(".*;", "", finalRes$Name)

finResTable = finalRes[,-1]

localRes = cbind(finResTable, names = geneNames)

# standard volcano plot

with(finResTable, plot(logFC, -log10(PValue), ylim = range(0,12), pch = 20, main = "Volcano plot WM vs RL with FDR", xlim=c(-2.5,2), col="brown"))

# plot p-value smaller 0.01

with(subset(finResTable, FDR<0.05), points(logFC, -log10(PValue), pch=20, col="red"))

# log2FoldChange > 1

with(subset(finResTable, abs(logFC)>1), points(logFC, -log10(PValue), pch=20, col="grey"))

# P-Value < 0.01 and logFC > 1

with(subset(finResTable, FDR < 0.05 & abs(logFC)>1), points(logFC, -log10(PValue), pch=20, col="orange"))

# plot some labels

with(subset(localRes, -log10(PValue)>7 & logFC< -1.5), textxy(logFC, -log10(PValue), labs = names, cex=0.8))

with(subset(localRes, -log10(PValue)>7 & logFC> 1), textxy(logFC, -log10(PValue), labs = names, cex=0.8))

with(subset(localRes, -log10(PValue)>9), textxy(logFC, -log10(PValue), labs = names, cex = 0.8))

legend(x = "topright", legend = c("FDR < 0.05", "logFC > 1"), col = c("red", "orange"), pch=20)

#with(subset(localRes, -log10(PValue)>10 & logFC>1.5), textxy(logFC, -log10(PValue), labs = names, cex=0.8))

#with(subset(localRes, -log10(PValue)>8 & logFC<2), textxy(logFC, -log10(PValue), labs = names, cex=0.8))

################################################################

#' volcano plot

################################################################

# standard volcano plot

with(finalRes$table, plot(logFC, -log10(PValue), pch = 20, main = "Volcano plot WM vs ALLE with p-value", xlim=c(-2.5,2), col="brown"))

# plot p-value smaller 0.01

with(subset(finalRes$table, PValue<0.01 ), points(logFC, -log10(PValue), pch=20, col="red"))

# log2FoldChange > 1

with(subset(finalRes$table, abs(logFC)>1), points(logFC, -log10(PValue), pch=20, col="grey"))

# P-Value < 0.01 and logFC > 1

with(subset(finalRes$table, PValue < 0.01 & abs(logFC)>1), points(logFC, -log10(PValue), pch=20, col="orange"))
