# Supplementary material for: Molecular signature of different lesion types in the brain white matter of patients with progressive multiple sclerosis
Source: Acta Neuropathol Commun. 2019 Dec 11;7:205. doi: 10.1186/s40478-019-0855-7 (PMC6907342; doi:10.1186/s40478-019-0855-7)

Supplementary Fig. 1

RNAscope: Negative (red and green targeting two bacterial genes)and postive (PPIB/POLR2A) controls

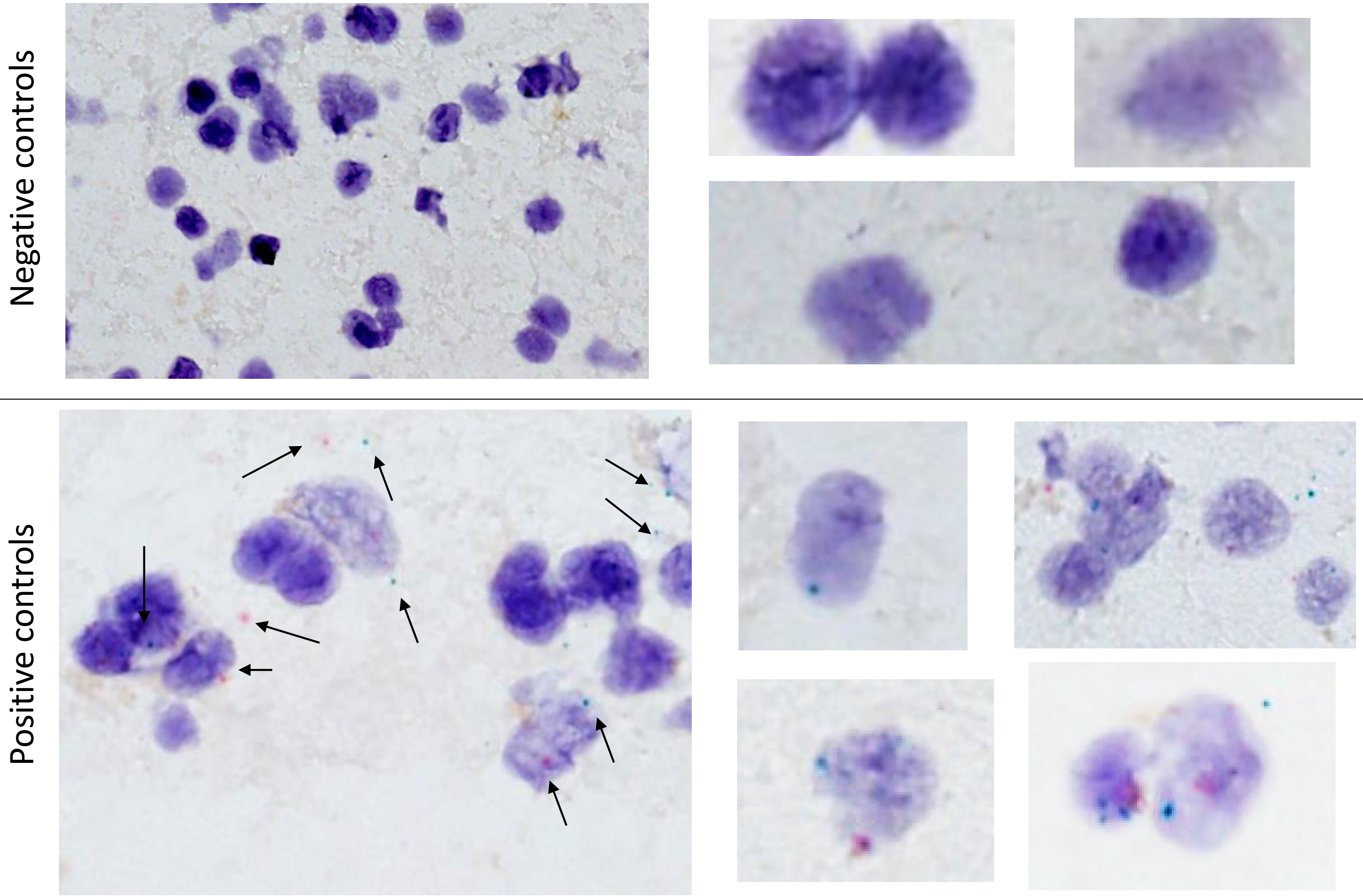

Supplement: Supplementary file 3 — Additional file 3: Figure S1. RNAscope : Negative (red and green targeting two bacterial genes)and postive (PPIB /POLR2A POLR2A ) controls [file 40478_2019_855_MOESM3_ESM.pdf]
