# Supplementary material for: Molecular signature of different lesion types in the brain white matter of patients with progressive multiple sclerosis
Source: Acta Neuropathol Commun. 2019 Dec 11;7:205. doi: 10.1186/s40478-019-0855-7 (PMC6907342; doi:10.1186/s40478-019-0855-7)
Supplement: Supplementary file 4 — Additional file 4: Table S1. Clinical and demographic data of the MS patients and non-neurological disease controls [file 40478_2019_855_MOESM4_ESM.docx]

**Supplementary Table 1**

**Clinical and demographic data of the MS patients and non-neurological disease controls**

| **Case** | **Sex**  **(f/m)** | **Age of death (years)** | **Cause of death** | **Death tissue preservation (hours)** | **Disease duration (years)** | **Time in progressive phase (years)** | **Quantified lesions** |
| --- | --- | --- | --- | --- | --- | --- | --- |
| **MS patients** | | | | | | | |
| MS408 | m | 39 | Pneumonia, sepsis | 21 | 10 | 2 | 6 (3 AL, 3 NAWM) |
| MS423 | f | 54 | Pneumonia | 10 | 30 | 21 | 6 (5 IL, 1 AL) |
| MS506 | f | 61/62 | Pneumonia | 21 | 50 | 19 | 9 (5 NAWM, 3 CA, 1 RL) |
| MS513 | m | 51 | MS, respiratory failure | 17 | 18 | 14 | 10 (2 NAWM, 2 AL, 5 CA, 1 IL) |
| MS528 | f | 45 | MS | 17 | 25 | 3 to 11 | 6 (4 NAWM, 2 AL) |
| MS530 | m | 42 | MS | 15 | 21 | 15 | 11 (4 AL, 2 CA, 2 RL, 1 IL, 2 NAWM) |
| MS549 | m | 50 | End stage of MS | 8 | 29 | 20 | 4 (2 AL, 1 RL, 1 CA) |
| MS576 | m | 75 | Pneumonia | 25 | 31 | Primary progressive | 9 ( 5 CA, 4 NAWM) |
| MS585 | f | 53 | Bronchopneumonia | 27 | 27 | - | 8 (7 IL, 1 NAWM) |
| MS403 | f | 54 | End stage of MS | 11 | 26 | 15 | 4 (2 AL, 1 RL, 1 CA) |
| **Controls** | | | | | | | |
| C25 | m | 35 | Carcinoma of the tongue | 22 | - | - | 5 |
| C36 | m | 68 | Cor pulmonale, heart failure | 30 | - | - | 5 |
| C48 | m | 68 | Metastatic colon cancer | 10 | - | - | 5 |
| PDC39 | f | 50 | Metastatic renal cancer | - | - | - | 5 |
| PDC40 | f | 61 | Ovarian cancer | - | - | - | 5 |
