# Supplementary material for: Molecular signature of different lesion types in the brain white matter of patients with progressive multiple sclerosis
Source: Acta Neuropathol Commun. 2019 Dec 11;7:205. doi: 10.1186/s40478-019-0855-7 (PMC6907342; doi:10.1186/s40478-019-0855-7)
Supplement: Supplementary file 5 — Additional file 5: Table S2. Number of samples, detected genes and significant genes in the post-mortem human brain samples [file 40478_2019_855_MOESM5_ESM.docx]

**Supplementary Table 2**

**Number of samples, detected genes and significant genes in the post-mortem human brain samples**

| **WM brain tissue** | **Number of lesions (n=98)** | **Comparison of gene count** | **Expressed genes** | **Significant**  **(FDR < 0.05)** |
| --- | --- | --- | --- | --- |
| WM | 25 | All MS tissue vs. WM | 18608 | 6712 |
| NAWM | 21 | NAWM vs. WM | 16713 | 465 |
| RL | 5 | RL vs. WM | 19472 | 3493 |
| AL | 16 | AL vs. WM | 17426 | 3415 |
| IL | 14 | IL vs. WM | 17538 | 4570 |
| CA | 17 | CA vs. WM | 17262 | 5739 |

NAWM = normal-appearing white matter; RL= remyelinating lesions; AL = active lesions; IL= inactive lesions; CA= chronic active lesions; FDR= false discovery rate
