# Supplementary material for: Molecular signature of different lesion types in the brain white matter of patients with progressive multiple sclerosis
Source: Acta Neuropathol Commun. 2019 Dec 11;7:205. doi: 10.1186/s40478-019-0855-7 (PMC6907342; doi:10.1186/s40478-019-0855-7)
Supplement: Supplementary file 6 — Additional file 6: Table S3. Lesion specific genes [file 40478_2019_855_MOESM6_ESM.pdf]

Lesion specific genes

Active Lesion	FDR	Remyelinating Lesion	FDR	Inactive Lesion	FDR	Chronic Active Lesion	FDR
AEN	0,0346681	BSN-AS2	0,002393824	RPS11	0,00658668	SLMAP	0,0311316
USP6NL	0,02117226	AC007036.6	0,011573019	DECR1	0,03133316	RALYL	0,001235
RAB33B	0,04786741	NOSTRIN	0,00993533	DDB1	0,02942181	PTPRR	0,0016301
CD53	0,03710795	SETP11	0,003138605	HIC2	0,01020412	MYO9B	0,0020758
PRPF3	0,04606266	RP11-373D23.2	0,011612889	RP11-77P6.2	0,02854829	XAB2	0,0153524
CAMSAP1	0,04160161	HMGB1P29	0,014870868	STK16	0,03689556	CENPQ	0,0347818
KCNK10	0,02884393	MARCOL	0,000916951	RP11-14I17.2	0,00308768	ATP5G2	0,0083566
RP11-513G19.1	0,01685352	CST7	2,17924E-07	MAX	0,02161023	CDK14	0,0074475
GPATCH8	0,04094743	CTD-2270F17.1	7,76298E-05	SPIN2B	0,03737081	RP11-981G7.1	0,0331641
HLA-DOA	0,00194793	ROR2	0,000410458	SCYL3	0,00204561	GRM2	0,0237033
MAGI1	0,00393283	GDF10	0,037876315	SUGCT	0,00804002	LONP1	0,016126
GALNT10	0,0481232	AC011243.1	0,037051022	GDPD5	0,03223516	SYN3	0,0235784
AXL	0,01270943	PWP2	0,02835798	DCAF4L1	0,03026691	TAOK3	0,030809
ATPIF1	0,00983227	RP6-127F18.2	0,020347279	CTDSP2	0,01126974	CMPK2	0,0069204
LRP1	0,01446273	LRRTM3	0,02685009	PABPC5	0,01259977	FAAHP1	0,0015727
NLK	0,00690775	SECTM1	0,012597837	HDAC7	0,01064747	CPNE4	0,030226
RP11-319G9.5	0,03937317	KIAA0368	0,034901106	XXbac-B562F10.11	0,001523	GTPBP1	0,0496899
SINHCAF	0,03073622	MEGF11	0,014593327	AC012494.1	0,02003063	CA11	0,0062989
P2RX1	0,00105767	RNF43	0,000175003	PLXNA1	0,00068598	ARHGEF9	0,0020758
ITGAX	0,00552976	PLAC8L1	0,009546169	PTP4A2	0,04899186	STK17A	0,0011331
GNG7	0,02284831	CTD-2017C7.3	0,04164315	RP11-77K12.3	0,01483404	SIAH3	0,0056268
ENO3	0,01510929	KIF5C	0,021486247	CXorf56	0,00111109	RMND5B	0,0209709
NOL7	0,00797694	RP11-513M1.1	0,038911595	TFR2	0,01586979	ZNF257	0,0021179
CTD-2127H9.1	0,02199505	RP11-831A10.1	0,005242946	LOC102724788	0,00312112	FOXM1	0,0364194
SLC41A1	0,01191022	SEPT7	0,044533341	DDHD2	0,00266026	MAOA	0,0031975
CTD-2525I3.6	0,03762911	ATL1	0,002044189	BTF3	0,02498499	RP11-210K20.2	0,0357824
MBNL1-AS1	0,02697454	INTS7	0,011737144	COX7A2	0,04117511	F8A1	0,0015379
CTD-2353F22.1	0,02275118	ZNF385C	0,013543285	RP9	0,02373654	PLEKHG4	0,0014849
AC004980.7	0,03058081	BATF2	0,005543618	UQCRHL	0,04847113	TPM3	0,0043775
LBH	0,00872089	HTR4	0,038961314	WASHC3	0,03475893	GCC2	0,0286573
MAP7D1	0,0492243	LPAR3	5,55031E-06	SCN11A	3,0461E-05	PMF1	0,0281394
ZNF682	0,045832	LINC01979	0,034953843	OSGEP	0,00678383	GAPDHP61	0,0127382
CYR61	0,04681291	LINC00595	0,014351712	NOP53-AS1	0,02637192	CD82	0,0131126
ARL5A	0,04831101	RP11-182I10.1	0,005393632	ATP7B	0,00066133	PIRT	0,022492

GGT5	0,00268093	GRK6P1	0,02769632	GRIK1-AS1	0,00045802	CPEB3	0,0357816
APOC1	0,01603545	ZSWIM9	0,044811327	EXOC1	0,01588841	CAMTA1	0,0492649
ZNF251	0,0444629	USP2-AS1	0,001945646	ETFA	8,0542E-08	HPCA	0,0001664
HCP5	0,04570502	PPP2R2A	0,033223689	RP11-230C9.2	0,00531048	MLF1	0,0161797
NFATC4	0,02740399	LINC02448	0,000415557	ADAMTS9-AS1	0,02271492	CHFR	0,037523
ARL11	0,02138815	VIP	0,000890772	CADPS2	0,01120746	AC006042.8	0,0053052
LOC374443	2,4878E-05	CDC45	2,89077E-07	TCP1	0,0227969	FAXC	0,0279601
ARHGAP45	0,04347857	RP11-326C3.11	8,84795E-05	EHD2	0,02162186	FUS	0,0418903
GSAP	0,01123341	FCRL5	2,27166E-29	PRPS1P2	0,04296046	EMC8	0,0013229
LSG1	0,02400352	COL4A6	0,003165525	ERMARD	0,00884815	RGS4	0,0100517
MAP4K1	0,04357252	LOC102724604	4,24509E-06	FPGT-TNNI3K	0,02619584	POLG	0,0181758
RP11-767L7.1	0,00849557	RNF139	0,0415735	NPIPP1	0,02912369	ADAM23	0,0043409
SPDYE3	0,03245916	HNRNPA1P59	0,009587352	SPDYE1	0,00012584	RP11-737O24.3	0,0111227
ARSB	0,00027913	SH3RF1	0,036642558	TDRD9	0,00477592	TMEM104	0,0176987
SEPT9	0,04873014	MROH6	0,015445787	RP1-30M3.5	0,03857495	PTPN5	0,0003315
MIGA1	0,02619124	GNG12-AS1	0,00079506	SHANK3	0,00804002	LAGE3	0,0327996
RP11-712P20.2	0,04312006	AREL1	0,026486518	TUFM	0,01161105	CHST6	5,254E-05
ATP5PDP4	0,02070863	IL9	0,000602185	ZNF839	0,01964948	OR9P1P	5,385E-05
ITGAM	0,04745476	COL11A1	0,021793986	CCNA2	7,2E-05	SLITRK4	0,0017755
ADGRD1	0,00952622	MYLK3	1,85497E-18	AUH	0,01020412	CACNB1	0,0436882
MOV10	0,04398626	NCOA7	0,019000762	PCYT1B	0,00553222	EML3	0,0153236
RP11-589N15.2	0,00657222	DENND6B	0,039636959	PIGT	0,03712668	KDM4B	0,0305817
LRRC55	0,00268543	FRMPD2B	0,000648158	CRLF3	0,0104876	PEX16	0,0019655
Z83001.1	0,00953076	ST6GALNAC1	0,035032008	PSMA5	0,01632687	CHRM1	0,0471971
NAV2-AS5	0,0182194	SNORA14B	0,001384317	FKTN	0,02729275	FUT4	0,0402173
CCL28	0,03820757	IL2RG	3,8819E-08	BANF1	0,00839409	CDH8	0,0031031
FAM241A	0,00471583	ZAP70	7,39406E-05	ZNF287	0,00631041	COMMD5	0,0391089
RLIMP1	0,0171205	LA16c-313D11.12	1,8181E-05	ZNF571-AS1	0,02217867	GSTP1	0,0157221
KRT8P12	0,01262704	LINC02226	0,005378749	MAGEF1	0,00339101	ANKLE2	0,0357413
RNF212	0,0216833	RP5-912I13.1	0,004370268	MCF2L	0,02394086	NAMA	0,0035475
VEGFD	0,00169602	ACACA	0,012764991	USP17L7	0,0201185	NADSYN1	0,0008055
ENPP7P10	0,00528658	GALNT13	0,044320302	PPP1R15A	0,04884435	FAM221B	0,0456617
RP11-116O18.3	0,03921184	RPS4XP16	0,034258257	NEIL2	0,04301961	RPS21	0,0037851
RP4-724E16.2	0,01043241	CTSW	0,020906893	FSD2	0,01582475	LMO4	0,0280404
GSDMD	0,01083933	ZIM2	0,018567887	LRRC32	0,00312545	RP1-111C20.4	0,0015599

Lesion specific genes

POPDC3	0,01863947	SYNE4	0,00578022	COMMD1	0,00483907	CHL1-AS1	0,0004283
IL7	0,04561897	OTX1	0,034155537	KRTCAP3	0,02255765	PPP1R14BP3	0,0156901
NBPF15	0,00943273	EMID1	0,024737024	LOC102724532	0,04436812	H3F3AP2	0,0179234
CYCS	0,01979853	TDH	0,006107709	GMDS-AS1	0,01910089	DCX	0,0387499
NWD1	0,00247489	PLD5	0,020376439	C19orf18	0,0002533	PPARGC1A	5,288E-06
RASGRF1	0,01139396	LINC01376	0,021014611	CYB5B	0,00452526	POLRMT	0,0319751
SLC6A15	0,03177472	FUT8-AS1	0,036162972	LOC105377348	0,0089456	RP11-1023L17.1	0,0027712
FRRS1	0,02190818	SLC6A20	6,26511E-06	RASGEF1B	0,04145577	CASC4	0,0171996
PCK2	0,04164672	FXYD4	0,033430758	NCAM2	0,02606287	CENPJ	0,018787
GMFG	0,00299278	HMGA1P7	0,009174788	FANCM	0,00555589	JUND	0,0390017
HTR1DP1	0,02411265	PLCE1-AS1	0,029333143	GLT8D1	0,00946479	AC007566.10	0,0042491
NDNF	0,03378279	DYNLRB2	5,37544E-06	RP11-631N16.2	0,00346512	CAMK2B	0,0494869
DHFR	0,02473348	PABPC4L	1,29E-06	SEPT2	0,02442679	TMEM241	0,0236933
GPR132	0,0486947	ATG9B	0,032055481	ZBTB21	0,01227891	NDUFB8	0,0325527
GNAO1	0,00320255	SNORA11	1,44451E-07	QRICH2	0,00476415	PSD2	0,008006
HARS	0,01812942	AP001172.3	0,001801954	SHISA4	0,04129461	PREX2	0,0152282
ATG7	0,02127658	CYP51A1P2	0,003012564	TMEM50B	0,01526665	ASTN2	0,0011315
POP7	0,03659297	EPPK1	2,94704E-07	NUDC	0,04743291	SCRN1	0,0460125
RP11-250B2.6	0,01270633	MYO3A	0,00215261	TCAP	0,00197848	DDI2	0,000226
DENND2A	0,0131519	GRID1-AS1	0,038025331	RBBP7	0,01444002	PFN1P2	0,0152243
ZC3H12C	0,01809431	COX18	0,045483208	TXNRD1	0,00028273	TANC2	0,0270624
GPR18	0,02977911	LINC02028	0,009946806	ARF5	0,04668077	TMEM189	0,005183
GYG2	0,00122119	PHYHIPL	0,040976897	CCT3	0,02770102	NTN4	0,0053363
LIX1L	0,02185276	AC091167.3	0,016238578	SLC3A2	0,00639918	PIGG	0,0012494
CYTH4	0,0244976	ANKRD20A21P	0,003165525	NTSR2	0,01636197	AC068282.3	0,0001158
FCGRT	0,00943273	RP11-795J1.1	0,02710283	KLHL31	0,02610102	PRRT1	0,026501
YWHAG	0,00909923	PROB1	0,009914012	TRIM52-AS1	0,02085285	KALRN	0,023432
NCBP2AS2	0,0377567	ARHGAP31-AS1	0,011903556	RP11-203B9.4	0,0243418	THOC7	0,0172178
FLJ16779	0,01796297	PEG10	0,006654577	HEY1	0,01922606	NEGR1	0,0450067
FSCN1	0,00816423	SIGLEC7	0,015445787	POLE	0,02218767	FAM49A	0,0038005
CLEC7A	0,0330756	SNED1	0,012795031	TRMT10A	0,01343304	GRPEL2	0,04051
ZNF423	0,02298038	RAET1E	0,026174021	ZNF559-ZNF177	0,04327469	CACNG2	0,0089464
C1orf54	0,03817747	ACOX2	0,005170033	TMEM64	0,04076783	NCK2	0,001017
IL4R	0,0396499	RP11-1055B8.2	0,020498522	ADAM8	0,00025522	CLDN10	0,0060667
BEX3	0,01591602	ANP32B	0,036770606	DTD2	0,02899671	HPCAL4	0,0045221

ELK3	0,0356742	PAPOLB	0,019553242	SLC2A2	0,03432468	CACNA2D3-AS1	0,0031872
LDOC1	0,0167868	GNL2	0,047107324	LMCD1	0,02519651	FBXO3	0,0170538
RNASEH1	0,00590524	RP11-22L13.1	0,001321807	WARS2	0,02095915	NOL4L	0,0160816
WLS	0,01233937	MUM1L1	0,025093584	MBOAT7	0,00201546	DICER1-AS1	0,0053363
CDYL	0,03958853	AK3P5	0,024214974	CTSO	4,6276E-08	AC000120.7	0,0348471
KLF11	0,01844309	PAIP1	0,023592936	KIF22	0,02041158	ARHGAP26	3,652E-05
TMEM86A	0,00373769	NDUFS1	0,021425652	WDR11-AS1	0,02068917	PRCD	0,0051547
RP11-666F17.1	0,00066389	SEC1P	0,005202621	PLA2G12A	0,00760683	GEMIN6	0,0066543
ZNF407	0,04617666	HIST1H4E	0,047877517	ELOB	0,04694821	JAGN1	0,0112269
MTTP	0,02186921	GOT1	0,029433559	TIMP4	0,02836884	BSDC1	0,0095812
PIM1	0,02984519	BBC3	0,023777454	LONRF1	0,03863182	ARSK	0,0092318
NPAP1	0,0079003	RP11-531F16.3	0,04102881	KCNMB4	0,04366488	LINC00641	0,0172178
TMEM246	0,04179094	C10orf67	3,57719E-05	FUT2	0,0255969	MAP6	0,003189
LOC101928909	0,03820757	KL	0,032055481	AATBC	0,02650466	OR2L13	0,0108975
RTL5	0,03218301	DNAH12	0,000215919	METTL17	0,00339336	PPP6R2	0,0081643
DDX60	0,00858486	RP11-486O12.2	0,049855107	IRF9	0,01519612	RNF175	0,0012626
LINC02018	0,02479388	ANKRD18CP	0,017918842	KB-1460A1.5	0,00058583	PCNX2	0,0390894
TBXAS1	0,00661697	CLEC3B	2,79582E-05	FKBP11	0,04436812	ARSA	0,0479658
SALL2	0,04791591	IGFBP7-AS1	4,70511E-05	SESTD1	0,02679653	AMER3	0,0212026
CYP2E1	0,00039589	NGDN	0,027733876	AP2A2	0,00238667	RP4-555D20.2	0,0350768
WDFY4	0,03447927	SPANXA2-OT1	0,006053099	HSD3BP5	0,00151736	LSMEM2	0,0022136
TBP	0,01649133	AC078883.3	0,016202926	EXOSC3P1	0,03832645	MBD2	0,0061359
GVQW1	0,0444739	TRGC1	1,11204E-06	EHHADH	0,00843056	CCDC28A	0,0074845
CMTM6	0,00846691	OR6W1P	0,044366253	LAPTM4B	4,6269E-05	PIAS1	6,227E-05
MRPL44	0,03950353	KCNG4	0,011041728	NR1D1	0,03413577	TSEN54	0,0225222
LONRF3	0,03995105	RP11-1391J7.1	0,001393534	MLYCD	0,04033813	RERGL	0,0060497
LINC00654	0,01191022	P2RY8	4,74323E-14	LOC653160	0,04610798	DPP6	5,465E-05
HADH	0,00600272	RP11-557H15.5	0,000498567	RIDA	0,00020442	CR1	0,0140533
ZNF367	0,0313914	TNFRSF10C	0,00616312	ZNF85	0,04726936	HMBS	0,0082678
CMBL	0,00249147	LINC01763	0,00280003	AC093495.4	0,04311227	PWAR1	0,0002074
RP11-2J18.1	0,04292855	TSPAN2	0,00734021	MUSK	0,00874333	PLPPR5	0,0236933
LPAR6	0,01844309	CALM2	0,016599737	SH3RF3	0,02435096	RP11-549L6.2	0,0010016
FAM122C	0,04716392	CTD-2373H9.5	0,044838942	STRADB	0,01400724	AL139147.1	0,015491
BTBD19	0,00886971	SNORA37	0,00361401	TIGD6	0,02421427	GRIN3A	0,0025392
CD300LF	0,01993007	FAM181A-AS1	1,27E-05	MRVI1	0,01307299	CHTOP	6,442E-05

Lesion specific genes

PDLIM3	0,00254887	RP11-367B6.2	0,014869517	ZNF581	0,02002752	SMOC2	0,0150988
UNC93B1	0,02564369	RP3-395M20.3	0,024252774	DDX27	0,01363747	ARHGDIB	0,0239777
ARPIN	0,00138615	INSM1	0,021271051	FAM192A	0,03517264	PTPN23	0,0287103
ADAMTS10	0,02392518	OR4F4	0,000827793	RP11-508N22.12	0,04855826	CACNA1C	0,042067
NBPF9	0,04119051	GYS2	0,002044189	LINC00969	0,02330564	ZNF676	0,0008819
ZNF30	0,03043727	PLAT	0,024470396	AZIN1-AS1	0,01199599	UCKL1	0,0209452
LAMA4	0,01521638	CASC15	0,033430758	TMC2	0,00168444	COX16	0,0251978
SEC14L1P1	0,03221405	SPRY4-AS1	0,020520473	ZNF8	0,03330491	OCEL1	0,0212826
ZBTB49	0,00902931	RNU6ATAC	0,002808068	PSMB6	0,01048502	CFAP36	0,0272948
STX4	0,01588828	RPL34	0,034443806	LRRC75A	0,00297929	IMPG2	0,0006009
VDAC2	0,02878593	HSPA12B	0,036716704	NOVA2	0,00652942	CBWD1	0,0026888
ZNF622	0,03209564	RP11-815J21.1	0,037762066	SPRTN	0,0072792	CKMT1B	0,0039257
LINC01004	0,0333973	NDUFA1	0,031201564	RAD17P2	0,0003748	PCDHB14	0,0135346
NCF4	0,04405117	RP11-276E15.4	0,013326927	ARL4C	0,00032504	CD276	0,006001
SIRPB2	0,01601434	RNU4-1	0,045483208	LPXN	0,01297801	SRP14	0,005661
B3GNT7	0,04334776	IGHG3	7,16083E-23	CCDC13-AS1	0,04006884	TCEAL2	0,0014598
FAM110C	0,04698675	USP27X-AS1	7,9558E-05	CRHR2	0,04763288	FAM153B	0,0110565
GBGT1	0,0025873	MAPRE2	0,000170352	PRKAA1	0,00315583	MKLN1-AS	0,0052332
EDRF1-DT	0,01330282	TCTE1	1,42353E-05	FADS2	0,02701803	KCNS2	0,0327231
FERMT3	0,00450356	CCL5	1,6358E-17	CYP4F3	0,04956138	CLIC4	0,0066675
JPX	0,02765999	SPAG8	0,00839336	TMEM230	0,00042694	RP11-36N20.1	0,0058085
SLA	0,0203042	AKR1C1	0,003702653	EGR1	0,00792433	EPC2	0,0285376
SLC29A3	0,04314326	RP13-270P17.2	0,000176314	ISPD	0,00723508	FMO3	0,014774
MYC	0,024056	RHPN2	0,024640527	RP1-179N16.6	0,00322754	MDM2	0,0394409
HLA-DQA1	0,00935734	RP4-669H2.1	0,000143688	NFYC-AS1	0,0072837	LOC100507156	0,0113019
UCP2	0,03108859	SP8	0,006999782	CNTF	0,0272234	SNHG15	0,0002951
SLC25A30	0,00202868	CYCSP23	0,014077263	RP11-196G11.4	0,0230488	NETO1	0,0090794
ASPN	0,00422792	PAGR1	0,039255687	STC1	0,00099504	ABCA6	0,0082988
SLC39A4	0,00909514	RP11-230B22.1	0,00158401	CPSF3	0,04282916	NBEA	0,0067778
ZNF114	0,00970687	FAM167A-AS1	1,34316E-09	RP5-921G16.1	0,03696877	TESPA1	5,385E-06
RP11-245J9.5	0,00435574	MCM8	0,034108605	CWF19L1	0,02808693	OR2H2	0,000487
SH3BP2	0,02686324	C2orf40	0,007607751	GPKOW	0,00470824	DZIP1	0,0013131
LPIN2	0,01614446	SLC16A1	0,044433387	LOC101927314	0,01313782	SEC22C	0,0144781
SCIMP	0,00360295	RP11-725G5.3	0,00077635	LNP1	0,01226375	RNF146	0,0204619
PTPN6	0,03511891	RBP5	3,5216E-05	EDNRB	0,00838181	SNX32	0,0347536

GRK4	0,02340911	C17orf97	0,018854767	ITM2C	0,00846602	RP11-64B16.4	0,0293798
HERC5	0,02849035	NDUFB1	0,023189638	ASRGL1	0,01309342	TMEM196	0,0319516
TRAF3IP3	0,02411265	TAF5L	0,035966681	ELP3	0,01154612	CREB1	0,0410204
RP11-211G3.2	0,03544715	TOMM20	0,032585745	UBE2B	1,2106E-05	RYKP1	0,0349218
NRSN2-AS1	0,00222497	ALPK3	0,009881359	S100A8	0,04382976	MXRA7	0,000399
CRB1	0,00039859	RP11-121A14.2	0,000138642	C1orf140	0,01026217	ZHX2	0,0065674
MBNL2	0,02624787	SLC22A18	0,019756656	PLEKHF2	0,04946033	LRRC49	0,0340651
RAB42	0,00625655	LOC101928489	0,018990832	LIX1	0,00088723	NFE2L3	0,0465448
RP11-274B21.9	0,01191022	CBX1P2	0,034005027	TNFRSF12A	0,03673183	G6PD	0,039411
OSMR	0,00872804	YWHAE	0,011966219	SERPIND1	0,00143844	DPH7	8,195E-06
SOD3	0,02945696	PCDH9-AS3	0,048188278	IFRD1	0,03509751	LPCAT4	0,0081531
NCF2	0,00909923	LRRC17	0,044930381	RP5-826L7.1	0,00843071	TMEM52B	0,0342591
KBTBD6	0,00224636	TUB-AS1	0,001892743	LINC01277	0,01269102	CMTR1	0,0169221
PRRG4	0,0444739	ADORA2A	0,000877427	MIR4697HG	0,01088945	UBE2H	0,0392441
DSC2	0,03488796	LINC00649	1,41271E-10	RP11-49K24.8	0,00082058	SRGAP1	0,0137211
TAPBP	0,01873205	MROH7	0,013459481	MGAM	0,00296708	RP11-98J23.2	0,0038844
NR2E1	0,00217246	CAVIN1	0,032707038	GLIS1	0,03310459	C8orf31	0,0363513
ABTB2	0,01237608	CFAP99	1,15015E-11	PTMA	0,00478709	FUT11	0,0012154
SDCBP2-AS1	0,03455922	IRF6	2,28928E-07	LINC02427	0,00090907	FAM184A	0,0005952
P2RY2	0,02101042	RP1-130L23.1	0,002127989	ETNPPL	0,00011545	LINC01018	8,772E-05
RP11-553K8.5	0,00510742	LINC02344	1,47162E-06	DNAJC14	0,01386583	LRMP	0,0044915
TMEM169	8,5578E-05	CTF1	0,041880982	MAPT-IT1	0,00064024	ATP2B4	0,0471523
ATP1B2	0,041098	FAF1	0,027470324	UBXN8	0,03671022	KANSL3	0,0188442
GNL1	0,00718729	IGHM	1,26779E-24	ZDHHC11	0,0174134	NME5	0,0021305
PSG1	0,00360399	SS18L2P2	1,20127E-06	RFWD3	0,04669596	ADAR	0,0382906
CAMK2N1	0,03060543	STX11	0,04934261	LOC101927164	0,0081633	PSKH1	0,0156021
B3GLCT	0,00336549	TTLL11-IT1	0,009296258	PTPN1	0,01895687	TOX2	0,0100699
CPEB1-AS1	0,01492257	CTA-963H5.5	0,041341818	LEAP2	0,03732864	LIN9	0,000201
PTPN7	0,02432995	FAM166B	1,14399E-09	SERPINE2	0,03326368	CROCCP2	0,0224753
MRPL43	0,02729831	ERLEC1	0,043174936	RP11-453N18.1	0,03173172	ZFYVE16	0,0050972
TBL1X	0,00200838	RP4-647C14.2	0,035702388	HIBCH	0,00103305	LINC01480	0,0456812
COQ6	0,02382428	GOLGA7	0,039045649	TIMM10	0,00090486	LOC100505635	0,0055475
LSP1	0,00109109	RP11-13K12.1	0,007010401	MED17	0,03113628	TBC1D22A	0,0061154
SYK	0,03224205	RP11-706O15.5	0,000501132	TMPRSS5	1,2408E-05	FRG1JP	0,0001024
PAMR1	0,02395171	TLE1	0,001817328	ATP6V1E2	0,01359047	CTB-35F21.1	0,0001711

Lesion specific genes

TMEM109	0,02343938	TBCA	0,042200509	ATAD3C	0,00069523	AC069213.1	0,0083615
NALCN-AS1	0,04677779	RASGRF2-AS1	0,020581091	GDPD3	0,01326882	PRRG3	0,0328083
ST14	0,03608934	ARHGAP19-SLIT1	0,02854827	FIGNL1	0,04967924	LY6G5B	0,001337
CARD8-AS1	0,0481232	RIBC2	1,22972E-11	SNORA74B	0,02899769	PRDM5	0,0112269
DDX51	0,02446922	AF064858.8	0,022229656	CEP76	0,02599721	KCNV1	0,0191657
HLA-DMA	0,012064	HK2	0,037300979	LINC01315	0,01193797	RP1-228H13.5	0,001474
HLA-B	0,01184322	LOC101929227	0,02808226	LOH12CR2	0,00215381	PTGFRN	0,0200268
PTP4A1	0,00497121	RP11-160H12.2	0,012441109	ABCA17P	0,00059255	SLC9A7P1	0,0003099
PYGM	0,03273387	LILRB5	4,20472E-10	CBWD3	0,0061583	NEUROD6	0,0141977
CSF1R	0,03439734	CFAP161	4,69926E-07	CORIN	0,01810447	CEACAM19	0,0333296
AOAH	0,0377567	AC022154.7	0,049560149	SNHG17	0,04160418	FAM214B	0,0021998
SLC25A53	0,04066255	SERPINE1	0,010313313	SMU1	0,03473954	C20orf194	0,009485
NADK2	0,04052753	FAM124B	4,0233E-08	RASD1	0,02497606	TNRC6C-AS1	0,0018153
A2ML1	0,0377567	USHBP1	0,019143109	MRPL40	0,00012237	NDUFA12	0,0081188
RP11-876N24.4	0,00207694	RP11-16L9.2	0,000478769	LY6H	0,03599562	TCF20	0,0242394
BAMBI	0,02379351	SDR42E2	0,000344583	NOS3	0,02650466	TMEM272	0,0229294
LTBP1	0,01426926	SPAAR	0,028026286	ETAA1	0,04604596	ELMOD3	0,00503
TRIL	0,01961847	SAMMSON	0,032729861	TMEM198	0,02446563	OIP5-AS1	0,0143313
BRSK1	0,01629031	MLPH	8,44727E-08	C1orf226	0,01927274	GPR63	0,0001102
SMCO4	0,00066389	C8orf34-AS1	3,2306E-06	PI4KAP2	0,008622	TMEM232	0,0203248
PLEKHO2	0,02023595	HSPG2	0,016220146	C9orf170	0,00125841	PPP1R21	0,0010016
HCG27	0,01629031	SUCLA2	0,034155537	RBMX	0,00931982	CHRM3	0,0187731
SF3B2	0,03224205	RAD51AP1P1	0,018648045	CTNNAL1	0,00994056	NEK9	0,009416
RP11-429G19.3	0,0301369	RP11-262H14.3	0,000458732	HERC4	0,03343116	RIMKLA	0,0028878
LPAL2	0,04570502	RP11-662G23.1	0,028408636	PEMT	0,02724413	PDCL3P5	0,0137912
NUFIP1	0,02355352	RP3-342P20.2	0,025485665	PARP3	0,0278114	IGSF6	0,0130129
PTTG1IP	0,03070386	RPL19	0,049175766	CATSPERE	0,02619584	HAUS7	0,034441
ARHGAP26-IT1	0,00019564	AQP9	0,000434136	NDUFA3	0,0417428	ATP2B3	0,0167965
EBI3	0,01879923	ST8SIA6-AS1	0,000226895	KYAT3	0,03753015	KDELR2	0,0126896
VSTM2B	1,5808E-05	FAM98A	0,028673912	SNX24	0,02471678	RPN2	0,0448788
ADA2	0,01360221	ISM1	0,022580435	RASL11A	0,00502233	MARS2	0,0027151
RP11-39M21.2	0,00211012	CTC-369A16.2	0,038718264	AC009120.4	0,01824358	IGFBP3	0,0158554
ISLR	0,01541577	RP11-147L13.2	0,000876201	LRRC37A	0,0035374	UPF2	0,0074247
AGAP6	0,00673578	RP11-82O19.2	0,049744988	PCDHGA6	0,01954826	ARHGAP24	0,0062172
TMEM38B	0,03718744	TNFAIP6	8,37795E-07	PAM16	0,00521707	JPH1	0,0004173

PATL2	0,00165875	LINC01597	0,003821292	CCDC112	0,02452143	EFR3A	0,0148725
ST5	0,02549521	RP11-1319K7.1	0,020196064	MTMR11	0,00783028	LOC283922	0,0199892
ZNF143	0,02926233	CD8A	4,69207E-07	ALDH9A1	0,01599468	SLIT2	3,236E-05
ICA1	0,00928084	AC019048.1	0,014637655	RP1-68D18.2	0,00429451	LINC02473	0,0101297
MX1	0,002603	CYP4F12	0,037762066	SLC18B1	0,01335838	HMGXB3	0,0160724
ZNF564	0,01804338	HERC3	0,010121614	SLC27A5	0,04574963	LINC00663	0,002149
LAMB2	0,01058034	CD72	0,000382123	C11orf54	0,04418923	ZNF804A	0,0407332
CFLAR	0,04990528	LYPLA1P3	0,010618885	PRKAG1	0,01659769	GDF7	0,0199533
RP11-469A15.2	0,04025389	VEGFA	0,018159378	ZNRF1	0,00027904	KCNJ16	0,0134274
TMEM107	0,0287028	RP11-433A10.2	0,024153044	ERVH48-1	0,00239539	PTDSS2	0,022797
TEX29	0,0464215	APOBEC3F	5,82611E-05	DCAF8	0,03307092	RAPGEF4-AS1	0,0025726
RP4-657E11.10	0,04242835	ADAMTS6	0,042569658	HPSE2	0,00059737	SLC25A23	0,0001955
CYBA	0,03267142	ALG1L6P	0,034208901	RP11-355B11.2	0,02810256	SETD4	0,0125428
CTNND2	0,02986886	LINC00900	0,000823578	RP11-927P21.1	0,0351101	RHOBTB1	0,0262755
TRIM22	0,00051964	MSC-AS1	0,042375642	HSD17B1	0,00353886	CHPF	0,0319516
HLA-E	0,01192155	RP11-986E7.7	0,000417005	ARAP1-AS2	0,00592642	KRR1P1	0,0218752
PODXL2	0,01496036	ATP5A1	0,03109209	IMP4	0,01292624	RP11-495K9.5	0,0069162
SFSWAP	0,01025382	RP11-483L5.1	0,008305943	CHRM5	0,03330491	NRG3-AS1	0,0012457
RP3-476K8.3	0,02848875	PTPRQ	0,000115222	FGFR1	0,00272402	ZNF214	0,0002336
AGAP9	0,02827061	AC002366.3	0,042069579	SCAMP1-AS1	0,00010102	MRPS18B	0,0044626
RCOR2	0,02175404	HEATR9	0,047751268	ACSBG2	0,0255969	RPL14	0,0185449
C4orf19	0,00708929	PKHD1L1	0,00049145	GPC5	0,04333485	TMEM208	0,0002844
MARVELD1	0,01925316	CEBPA-AS1	0,001975154	AC005003.1	0,02155049	ILF3-AS1	0,0014764
RP11-319G9.3	0,01565085	PEG3	0,029128105	DCP1A	0,00121543	DDX20	0,0079917
CCSAP	0,02346138	STK32B	0,039204166	RP11-286N22.8	0,04591011	TBC1D8B	0,0159738
AC144835.1	0,00440599	CFAP65	7,13126E-05	SPIRE2	0,03837233	FAM92A	0,0077615
RP11-152L20.3	0,00688868	PIGU	0,044811327	RBPJ	0,01765998	SYT4	0,0115594
CCL2	0,01037828	PRNCR1	0,02946465	GTF2IP4	0,01850351	GPR75	0,0003776
ZNF192P1	0,00035775	C20orf96	0,014847739	ALDH2	0,00549177	PCDHGA3	0,0017118
KIAA0040	0,0371658	AC009110.1	0,006740666	RP11-1148L6.5	0,03802302	SLC38A11	0,0004214
NSUN2	0,03954666	UBR4	0,03175098	OTUD4	0,02936789	NSUN4	0,0256416
ATP5O	0,04507683	RBM48	0,004075885	LOC101928725	0,00013211	ANKZF1	0,0439955
S100A4	0,01368231	CCNA1	0,045436321	RACGAP1	0,04738957	TRMT2A	0,0412475
DNMT1	0,03100266	ID3	0,023947787	C14orf132	0,00339516	MEF2C	0,0002251
FMNL3	0,0075031	HSD17B3-AS1	0,011880217	DHFR2	1,1563E-05	AFF3	0,0089704

CTC-525D6.1	0,03212252	PLAC9P1	0,001811934	DAO	5,7648E-05	ERBB4	0,0368227
PLA2G15	0,04593789	NRM	0,025310188	BDH2	0,00052031	RP11-689P11.2	0,0180804
ZNF69	0,04677779	CCNL2P1	0,044643681	AC024560.3	0,03917322	IL12RB1	0,0388208
SASH3	0,03290614	PRR29	0,003885929	BACE2	0,00253813	RP11-351I21.6	0,0262771
NAP1L4P1	0,00363092	SHISA3	0,004937386	LOC103611081	0,01632687	MPPED2	0,0118126
HIST1H4F	0,00197494	CD36	4,17543E-09	CLSTN2	0,04275022	CHRDL1	0,0030738
ST6GAL2	0,04634891	XRCC6	0,002165324	STK40	9,4532E-05	TAF4B	0,0122167
RUSC1-AS1	0,01252419	ADGRF5P1	0,001257758	CBARP	0,04323594	GAL3ST3	0,0495318
PTGS1	0,01787491	LINC01695	0,003953483	CTAGE3P	0,0006496	STARD4-AS1	0,0163225
RP11-250B2.5	0,03544715	TCF12	0,007358049	PHF1	0,01495944	FBXO16	7,015E-05
NDUFS7	0,02272735	EIF4EP1	0,030869924	FBXO36	0,01169284	TUBGCP3	0,0213803
		RP11-174O3.3	0,001543112	MTR	0,01853292	CLGN	0,0397779
		RP1-117O3.2	0,008519368	RSPO2	0,03318799	SMARCC2	0,0017183
		ILK	0,028233102	PGM2L1	0,03587278	RAD51B	0,0498147
		AC005162.5	0,016220146	ANKRD52	0,04240639	DGCR9	0,0022057
		RASA4	0,018207167	PPL	0,02830498	ARHGAP17	0,0359746
		LOC101926933	0,006646877	TIPIN	0,00035972	LINC02495	0,0001968
		AC007405.4	0,036222554	BAALC-AS1	0,02980848	KCNT1	0,0085116
		NDUFA4	0,03612735	SLC25A18	0,00021144	TMEM127	0,0096159
		SOX17	0,00932432	PDSS1	0,02531382	THAP11	0,0487792
		AXDND1	0,039397206	PGAM1	0,03389198	NEK4	0,0232359
		NEUROD1	0,045593865	AC098617.1	0,00711883	CACNA1E	0,0223023
		SENCR	0,001492466	PMS2P4	2,8526E-05	PAFAH1B1	0,0129263
		NDFIP2	0,044930381	RN7SL752P	0,00217667	RP11-203J24.8	0,0013381
		VDAC3	0,046381399	PARK7	0,033719	CNTN4	0,002772
		RP11-607P23.1	0,009090245	PSMD8	0,04365612	CTD-2303H24.2	0,0035693
		ANP32C	0,001482499	TRAPPC2	0,02616314	SLC25A1	0,0019657
		PODNL1	0,000138673	MSANTD3	0,01003162	PELO	0,0480881
		FUT1	0,025157733	PTGES3P1	0,04421132	SYT5	0,0301895
		NOP56	0,031129959	ANKHD1-EIF4EBP3	0,03770908	RP11-650J17.1	0,0107465
		NLN	0,020581091	AC003080.4	0,00794179	TIPARP	0,010512
		GK3P	0,048039004	TRAPPC5	0,00117648	PGAP2	2,024E-05
		MIR142	0,002250841	PRDM6	0,03287601	IPO8	0,0257972
		NR5A2	0,000634382	RP11-327P2.5	0,01449876	ITPKB-IT1	0,0330598
		ODAPH	0,011254441	SLCO1B1	0,03525562	CCSER1	0,0073241

Lesion specific genes

NLGN1	0,009311296	KCNN3	0,00431628	NEFM	0,0121989
HELLS	0,042967161	RP11-274H2.5	0,02926919	SYN1	0,0460267
COL18A1-AS1	0,018272515	IGFN1	0,00316483	VSNL1	0,023408
CNTNAP2	0,01530864	RP1-146A15.1	0,02861851	CCDC186	0,0115953
BSCL2	0,029322235	C10orf143	0,03702735	LRRC4	0,0023163
SOCS2	0,048407092	ABHD18	0,01698083	ZNF318	0,0167259
NKAIN2	0,009146387	DOCK4	0,02445657	STRIP2	0,0039845
RP11-554D20.1	0,035032008	WWOX	0,00430867	GRIN2C	0,0468028
TTC6	4,63305E-07	CTSF	0,01448253	DCUN1D3	0,0199811
CTD-2058B24.3	0,005955886	BNIPL	0,00061089	DPYD-AS2	0,0200945
MAPK15	5,94811E-07	DLL4	0,04027791	CROT	0,0449388
ARSE	0,002476198	RN7SKP154	0,00190838	MTND4P12	0,0034413
ACOT4	0,034155537	SLA2	0,00907576	ESRRG	0,0027393
MTCO1P15	0,008716895	GPR79	0,00331559	CDR2	0,0129219
CCDC89	0,002313149	ZC4H2	0,01269935	GPR12	0,0097222
ATP5E	0,020846635	GGACT	0,01583616	FAR2	0,010043
PPP1R26-AS1	0,040293824	SIRT7	0,04327469	PDRG1	0,0051642
IGKV4-1	2,64929E-76	PPIL3	0,01495944	STRADA	0,0108238
KLHL3	7,76298E-05	IGF2BP2	0,04128916	RNF2	0,0232384
CES1	0,004492323	BPNT1	0,02379215	ABCD2	0,0305698
PTGIS	0,001328809	CCNJ	0,01328229	TM2D3	0,0224067
LOC100506384	0,011395328	IQCE	0,0266369	PEAK1	0,0183823
SLC52A1	0,000106396	NIPSNAP2	0,00152279	EYA1	1,622E-05
NPM1P18	0,004153331	PGAP1	0,02745984	WDR17	0,0401297
CLCC1	0,042179931	SRPX2	0,00241755	CLCN2	0,0482449
CLTA	0,009764721	AKAP12	0,00045755	ANXA6	0,005392
BISPR	1,55413E-06	TNKS1BP1	0,02208658	RP1-257A7.4	0,0266335
RPS7P7	0,001960063	LIPG	0,00012476	PPHLN1	0,0078773
IGHG2	1,45681E-14	DENND6A	0,03819407	AC144521.1	0,0012303
RP11-455F5.3	0,026767867	TUBA1C	0,00245684	DLG4	0,0379697
DNMT3B	0,035703508	ZFYVE28	0,00735099	RP1-86D1.5	0,0147373
TRIP12	0,049710288	SEMA6D	0,00092892	CTD-2516F10.2	0,0254699
RP11-575L7.4	0,002532807	MAPK13	0,00746029	ELN	0,0189198
BRD8	0,031103542	PCDHGB9P	0,0021481	RNF19B	0,0322923
TIAM1	0,037962164	RP11-792A8.3	0,04499469	MPI	0,0095459

Lesion specific genes

SYTL1	1,59485E-05	CALM2P2	0,02019411	TLR8	0,0361948
DDX24	0,009688966	LINC00910	0,00038439	PCDHA9	0,0170832
TMEM170A	0,021273171	NSMCE1	0,00586154	RRAGA	0,0183343
RP11-685N10.1	0,000498567	SETD1B	0,02198035	MAML3	0,0327231
RASAL3	0,04259663	ZNF606	0,02202634	FAM229B	0,0178579
PRKACB	0,008614104	SNRNP35	0,01025636	PHF19	0,0285815
BICRA-AS1	0,005978186	SH3GL1P1	0,00452947	CTD-3185P2.1	0,0073957
RP11-72L22.1	0,000885306	SMTN	0,00632803	PPP2R5C	0,0060149
HERC1	0,015999255	DDX59	0,03219063	FAM155A-IT1	0,0062398
COPS6	0,020630372	RP11-259K15.2	0,0208338	SIPA1L1	0,0034104
RP11-405O10.2	0,008335683	RP3-467L1.6	7,0267E-05	OXR1	0,0492649
TAS2R31	0,029157284	TMEM262	0,03484957	DGKB	0,0003435
ANKUB1	0,002051482	C18orf32	0,01064046	RP11-397O4.1	0,0102438
RP11-573N10.1	0,046671722	PLPP3	0,03467134	HK1	0,0213476
RP11-380G5.3	0,047751268	APH1B	0,00074822	VPS33B	0,0094253
NARF	0,048793026	F5	0,04584406	SLC35G1	0,0293339
JAML	3,38809E-10	AC002310.12	0,01143345	CDK5R2	0,0237771
MTMR4	0,012465071	HINFP	0,00121539	WAS	0,0097982
PRICKLE4	0,000143688	RP11-669E14.4	0,00674501	ARHGEF18	0,0160851
PALM3	1,91565E-23	PPP1R1A	0,00416002	RUNDC3B	0,0019822
LINC02097	0,026418079	RP11-234A1.1	0,04971546	RP5-926E3.1	5,403E-10
XIST	2,01526E-05	SMG1P7	0,01395373	CDH6	2,643E-06
PLEKHG4B	2,95959E-10	AFTPH	0,00778472	POLR3E	0,0010963
FBP1	0,031787376	COLGALT1	0,01991208	AC004019.10	0,0063335
SELENOI	0,04815844	FAM228A	0,03078652	GABRA2	0,0004525
PITPNM2-AS1	0,03218875	ZFAND2A	0,01025373	SEMA5A-AS1	0,0107072
PSMD4	0,032934978	PWWP2B	0,01244136	DENND5B	0,0044459
MICU2	0,046478649	HSDL2	0,00562888	RPL7L1P2	0,0008197
CTD-3138B18.5	0,00441953	SEMA6B	0,04335037	LCA5	0,0233738
CA3	0,01722657	AKR1A1	6,2272E-05	RDH16	0,0020668
SNORD99	0,001366647	LINC01135	0,0203803	TTC7A	0,017591
EME1	0,026477349	ENGASE	0,01095312	CTD-2002H8.2	0,0420435
BRCC3P1	0,049744988	INO80	0,00560141	RPL6	0,0295374
KIRREL3	0,005170033	PGS1	0,04614779	BRINP1	0,0295498
RPL37A	0,018784254	HAP1	0,00839447	ABLIM2	0,0088659

Lesion specific genes

RABL2B	0,024470396	AF129075.5	0,00599805	JAZF1	0,0137973
HAPLN3	0,032112533	MEP1B	0,00538449	RP11-116N8.4	0,0428186
NSUN7	0,01589474	BCAS3	0,0208338	ARFIP2	0,0447144
SLCO5A1	0,042017975	HSD17B6	0,00492431	POLQ	2,13E-05
RAMP2-AS1	0,027330011	FAM45A	0,01964948	MRPS30	0,0181395
ATP8B3	0,01901518	EFCAB11	0,00801069	FFAR1	0,0001529
GPR68	0,047303978	TMEM54	0,00867639	SLITRK6	0,0326988
PMP22	0,014035427	PITPNM1	0,0136181	ORAOV1	0,0208657
RP11-70J12.1	0,009753971	LINC01184	0,00369099	KCTD9	0,0052682
PSPHP1	5,30447E-05	RP11-1055B8.4	0,0056016	ERG28	0,0178064
SNORA35B	0,01088025	MATR3	0,032714	TLR3	0,0469174
PZP	0,001926688	SPTLC1	0,03404479	FGF14	0,0009895
MTND5P14	0,036359818	ODF3	0,01645271	CSRNP3	0,0068191
RP3-406A7.7	0,004336864	NUP210L	0,03149477	GLRA3	0,000796
C11orf70	9,36269E-10	METTL8	0,01062673	POLR2D	0,0035281
EPHB4	0,024870336	CTD-2380F24.1	0,02619584	KANSL1L	0,008655
HSPA12A	0,022169585	RNF141	1,67E-05	C4orf50	0,0096436
SLFN12L	1,9485E-10	DLGAP4-AS1	0,03031188	RP11-97C16.1	0,0406942
RPL39L	0,001474071	RP11-535M15.2	0,04630021	CACNA2D3	0,0184929
TEX45	0,03583583	RP11-400F19.18	0,0432237	GFM2	0,0034139
PLAU	4,49298E-06	DAP3	0,03326368	LANCL3	0,0153133
SAMD3	0,005820955	LINC02232	0,03030025	RBM12	0,0116369
NXPH3	0,027347668	EIF2B3	0,02299921	MYH14	0,0053722
POU3F4	0,017710194	RPA2	0,04247022	IFITM3	0,0292803
TTLL6	0,001909221	MXD4	0,00181157	RP11-58H15.1	0,0011474
TMEM159	0,021640147	NABP2	0,01470879	HRH3	0,0255259
CDKN2B-AS1	0,000762832	ULK1	0,02466118	PSAP	0,0345291
LINC01719	0,049828922	SLC2A3	0,00544534	CHM	0,0059484
GJB3	0,018355585	ACOX1	0,03538957	FAM105A	0,0240126
IL13RA2	0,007287165	GS1-358P8.4	0,04062356	ZNF619	0,0163293
VWF	0,04409998	TTYH3	0,01349018	RP11-785H5.2	0,0082364
BMP4	9,40823E-05	CAMK2D	0,01001374	LOC100128239	0,0020319
RP11-303E16.5	0,049257464	HIST1H2BC	0,04899864	RP11-580I1.2	0,0468161
RPH3AL	0,011804425	RP11-526I2.5	0,01583435	DNASE2	0,0338172
SH3TC1	0,013825472	E2F1	0,02940786	LGMN	0,0229338

Lesion specific genes

GRIA3	0,040140712	LINC00987	0,04477991	MTA3	0,0104236
CTA-221G9.11	0,000556306	MRPL54	0,00434156	CDH22	0,0017847
C5orf58	0,001428924	SIPA1L2	0,00707386	INA	0,0052084
COL13A1	0,00081441	GNB1L	0,00372944	DGKI	0,0252663
FAM86B2	0,036246784	ECM1	0,04107816	FAM136A	0,0414287
SNAPIN	0,033793717	FOXJ2	0,01044369	ENPP5	0,0054379
CCDC114	6,98356E-09	RNF219-AS1	0,01247926	RP1-269M15.3	0,0025308
AP004372.1	0,019597247	EGFL7	0,03690342	RBM42	0,0328157
HLA-DRB6	3,72523E-05	LRRC66	0,01271819	MBNL1	0,0347536
TPD52	0,014597767	DNAJC9-AS1	0,01181404	NUP160	0,0433491
AC007362.3	0,003549511	CCDC120	3,4218E-05	FGF14-IT1	0,0002
EFCAB10	7,09964E-05	CTC-487M23.5	0,00087175	NAXD	0,021653
MESTIT1	0,001915435	RHNO1	0,00654114	CYP2D7	0,015062
MAGI2	0,007872497	MAPT-AS1	0,02069248	SRP14-AS1	0,0382906
STX12	0,036615124	CNRIP1	0,04694821	PAFAH2	0,0199497
RP11-113K21.1	0,017241439	UNC13D	0,0230488	KCNQ2	0,0177755
PSD2-AS1	0,020134397	PPP1R3B	0,02318484	HTATIP2	0,0485076
MS4A4E	4,5719E-06	FHL2	0,03009082	LINC01137	0,0028263
KIAA1468	0,035407462	LPCAT2	0,02425387	EPHA6	0,0002432
LINC02301	0,040299184	LOC389831	0,04246244	COA6-AS1	0,0034993
LINC01992	0,000219245	CYC1	0,0431857	DPH2	0,0050733
LINC00426	3,47302E-07	RPL22	0,02268312	SLC20A1	0,0393895
PPP1R12BP1	0,027222487	SPATA6	0,00833123	LDB2	7,236E-06
ADH6	2,99415E-05	ALDH1B1	0,0481726	SPTB	0,0284385
RP11-298D21.2	0,010105238	SPEN	0,03755718	EFNB2	0,042167
PSMB1	0,042161516	TTC32	0,02178525	ITPK1-AS1	0,000608
RN7SL344P	0,01641357	MIDN	0,04991756	PATZ1	0,0036483
HKDC1	7,65086E-06	FRAT2	0,03552329	SHISA9	0,0001293
		RP11-799M12.2	0,01020412	VWA7	0,013318
		PGM5P2	0,01746507	ANKRD29	0,0016826
		RP11-678G15.2	0,03057329	LINC00926	0,0228291
		TJP1	0,03071263	PC	0,0350768
		PRMT6	0,00053819	PAK5	0,0003985
		MSANTD1	0,03296692	PDZRN4	0,0002557
		SLC17A7	0,04088327	COMMD3	0,0294777

Lesion specific genes

SERPINF2	0,00092567	MAP2	0,0056527
IQCB1	0,02329596	PANX2	0,0002243
CCDC62	0,04692824	ZNF280B	0,0146763
RAPGEF4	0,03095689	FAM210A	0,000266
MAZ	0,04150318	TMEM201	0,0205015
DCTN6	0,01930639	SLC12A5	0,0493144
RP11-304F15.3	0,00044054	WDR76	0,0001549
RPL17P50	0,00116429	OTOGL	0,0147281
KCNJ14	0,0258398	DSCR3	0,0001628
HSP90AA1	9,964E-05	ADAMTS13	0,0306316
THSD7B	0,03291138	TTYH1	0,0233873
BX322557.10	0,03552329	IGFL3	0,0077293
SLCO4A1	6,9359E-08	WDR5	0,0406456
GLUD1	0,00254682	ACVR1B	0,0113599
LINC01852	0,03531599	FAAP100	0,0423372
PML	0,02645289	ILVBL	0,0296473
UROD	7,23E-05	MATN2	0,0153125
LRP6	0,02821286	MPP3	0,0019152
LINC00609	0,04452339	ARHGEF6	0,0404823
CLCA4	0,04634405	CIB1	0,0278642
MLLT1	0,00799279	CIT	9,705E-05
CSTF2	0,04240639	RPL41	0,0401897
AC006538.1	0,04006884	MCM3AP	0,0067213
METAP1D	0,00433481	RP11-66N24.4	0,0335366
SGCA	0,02320849	ACSL6	0,0364194
JMY	0,04851195	CHAF1B	0,0099876
ZNF844	0,00830159	KCNB1	1,365E-05
TCAF2	0,03090126	TMEM261P1	0,0040182
LOC401127	1,5413E-05	UGP2	0,0004086
RP11-509J21.2	0,0063567	PRDX3P1	0,0025717
LINC00461	0,03176441	GRM4	0,0039431
DNAJC2	0,03583864	AL592494.5	0,0161907
LINC00271	2,1913E-05	ZMAT4	3,048E-05
TIMMDC1	0,02591422	CCDC22	0,0028776
LINC00205	0,002398	GTF2IRD1	3,236E-05

Lesion specific genes

HSP90B1	0,03145563	RP11-1020A11.2	0,0421278
C9orf131	0,03313904	RP11-731J8.2	0,0075953
GPR146	0,00215727	CPSF1P1	0,0056039
ATP6V1G1	0,03806921	SLC25A15P5	0,0033498
BANP	0,02155801	GPRC5B	0,0069511
AF127936.7	0,0003948	RP11-1415C14.3	4,789E-05
FTSJ1	0,01777937	SUMO4	0,0158783
ZCCHC8	0,03223516	IFT57	0,0262563
VN1R1	9,3588E-05	RP11-276H1.3	0,0001477
CREBRF	0,04473686	LMNA	0,0242216
INTS4	0,04056234	TMEM119	0,0144061
C8orf76	0,02288733	RP11-742D12.2	0,0372959
SPAG5-AS1	0,03347062	RP11-395I6.3	0,0201759
HSPA1B	4,6298E-06	MAGED2	0,0144936
CDK19	0,01672737	GTF2IP12	0,0039084
VWA5B2	0,02105284	GTF2H3	0,0007811
PDZK1	0,00146876	LHX2	0,0095812
CYB561D1	0,00273483	B3GALT1	0,0001754
SART3	0,03995321	TRNP1	0,0021989
RP5-890E16.2	0,04146403	GAD1	0,0286958
SNRPD3	0,01493024	ZNF133	0,0461122
FRG1B	0,02296756	WAC	0,0075376
SPCS2	0,03332152	WHRN	0,0392806
ITPKC	0,02481394	PTPN13	0,0083249
CEP152	0,01945503	PPP4R4	0,0001668
ATP5C1	0,00552048	GOLT1B	0,0288219
NTN5	0,00737953	SUSD1	0,0010729
RP11-770G2.2	0,04657856	ADD2	0,0178579
RNF13	0,00936299	PKD2L2	0,0326988
PAXBP1-AS1	0,00083933	ATP2B1	0,0027923
UBR7	0,04634405	ELMOD1	3,396E-05
GGTA1P	0,01086623	ATF7IP2	0,0008053
ARL17A	0,0272234	PRR3	2,521E-05
TMSB4X	0,04225308	KANSL1-AS1	0,0015287
RP4-535B20.1	0,02185883	ARNT2	0,0098915

Lesion specific genes

DHRS4-AS1	0,04666335	ANKRD20A7P	0,0494071
EEF1A1P19	0,00405843	CIPC	0,0406942
NPAS3	0,04412041	TFIP11	0,0030738
COL6A6	0,03255169	TACC2	0,0029371
RN7SL767P	0,01576754	NAALAD2	0,0059926
LOC101930085	0,0084572	TMEM139	0,0041299
GLIPR1L1	0,00373806	GNAL	0,0005366
CTC1	0,00876129	SH3BP4	0,0010307
TUBB1	0,00799279	ANKRD13B	0,0404913
RUBCN	0,03877792	MTCO1P11	0,035852
NUSAP1	0,01771537	RP11-214K3.20	0,0103803
BET1	0,0417428	PPP1R13B	0,0018791
IL11RA	0,03832645	RP11-530N7.2	0,0146645
HOMEZ	0,01788193	CD2BP2	0,0187151
NOS2	0,03802302	FXYD6	0,005154
RGN	0,00636077	RCL1	0,0041421
RP11-174G6.5	0,03638206	B3GAT1	0,0217559
COX15	0,03467134	BET1L	0,0243421
HEPH	0,01053503	SNX2P2	0,0014723
RP11-1060G2.2	0,01523489	FRY	1,343E-06
GEN1	0,00655439	RPL17P19	0,0056944
ZNF623	0,03829485	SAAL1	0,0076189
C1orf61	0,00125762	DNAJC27-AS1	0,0489854
LOC729970	0,04109953	FAM96B	0,0094926
HSPD1	0,0005228	BSN	0,0408683
KLHL36	0,01270142	ASPRV1	0,0001852
DESI1	0,01422965	TBL3	0,0488192
FBXL19-AS1	0,00804002	CPNE5	0,0085503
TONSL	0,04802815	RP13-93L13.1	0,0302405
AC003104.1	0,03326368	PRR16	0,0002163
RP11-536C5.2	0,03680812	CES2	0,0436317
LIMD1	0,04174877	MRPL52	0,0194261
CRHBP	0,02290793	AP000473.5	0,0010277
RBFADN	0,00810386	HS3ST1	0,0293637
GSTM4	0,00114085	METTL2B	0,0160635

Lesion specific genes

SPTBN1	0,03587278	INE1	0,0116864
ZNF552	0,00049104	RP11-259K5.2	0,0065766
NUDT12	0,00975249	C3	0,0304845
AGT	0,03745657	POLE3	0,0029847
GLIS2	0,00601995	PKIA-AS1	0,0160851
FGF1	0,01844001	NELFE	0,0422159
NFIL3	0,02079305	DLX6-AS1	0,0027923
MED14	0,01081159	RP11-588K22.2	0,0142465
TSN	0,03708255	MFSD11	0,0071765
DUSP18	0,0278425	STARD13	0,0156021
TRMT6	0,03454656	CDH10	0,0005631
C17orf53	0,00996452	RP11-968O1.5	0,0288712
TCEA1P4	0,00889444	PPP2R5A	0,0233138
TAS2R3	0,0021175	GPR19	0,0161344
TJP3	0,00830159	EBPL	0,0022793
CYCSP34	0,02463862	APOBEC2	0,0034915
RP11-268J15.5	0,01542399	OXTR	0,0026556
BRD4	0,00045211	USP19	0,0469416
NDUFA13	0,01526873	LINC01358	0,0349574
CRYBA1	0,01555444	NTAN1	0,0063416
PRR36	0,03859825	WDR6	0,0221838
AC074289.1	0,03642457	LEMD2	0,0104837
ZNF137P	0,00931982	MIS18BP1	0,0232676
ERMAP	0,04773963	CBFA2T2	0,046475
DEFB109D	0,00095869	RP11-429A20.4	0,0265268
KLHL21	0,01480049	RABEP2	0,0485316
PI15	0,02069271	LINC00938	0,0075781
RPN1	0,03470531	DKFZP434K028	0,0036973
TFE3	0,00075597	PPP1R12A	0,0121927
SNRPC	0,01340772	ZNF7	0,0264343
GTF2IP1	0,03355948	MLIP	0,0261335
MIR17HG	0,02905761	ZNF583	0,01884
GTF2H2B	0,01247926	PLXNB3	0,0176992
GABRE	0,00223805	EML5	0,0074445
PRSS37	0,00199342	NR1I3	0,0081149

Lesion specific genes

LOC653513	0,00716243	RP11-52J3.2	0,0431238
GPX2	0,00060071	RPS6KA6	0,0067551
SPDYA	0,03063394	TMEM140	0,016529
DTWD2	0,00505115	LRTM1	0,0023246
TRAPPC6A	0,0140185	SLC16A1-AS1	0,031496
ATG3	0,00759518	RASSF4	0,0391181
TESK1	0,01236271	PLA2G6	0,0349822
TRIM24	0,04286163	MRPS28	0,0367515
ANKRD18B	0,02460725	CMYA5	0,0027712
KRT18P4	0,01325894	RILPL1	0,0004584
ZFAND1	0,02050084	SIRT2	0,0041164
DUSP3	0,00247783	RP11-1280N14.3	0,0165907
CHMP2A	0,03812328	GANAB	0,0440555
C9orf153	0,00016798	NGFR	0,001093
TLR10	0,00413524	RP11-798G7.8	0,0058906
PSMB7	0,01082516	DKFZP667F0711	0,0463162
RP11-466A19.5	0,02726919	CACNA1H	0,0320571
TRIM4	0,01325894	CTC-429P9.3	0,0395195
LHX4	0,00434156	PRMT8	0,0233138
HSP90AA2P	0,00982962	TCEAL6	0,0011384
PACS1	0,01259977	ZNF827	0,0377594
RASA2	0,02816997	AURKAIP1	0,0142465
DBI	0,01013877	RP11-195C7.1	0,0193858
RNF152	0,0278114	RHCE	0,0249139
BBS4	0,0048292	C7orf49	0,000639
ZNF544	0,01638887	PANK1	0,0230841
CDR1	0,00438122	NSG2	0,0070348
C7orf61	0,00117648	PTGS2	0,0260288
TRMO	0,01495944	UBE2D3	0,0310186
KLHL18	0,022774	CTXND1	0,0082972
RP11-736K20.5	0,03925639	NEFL	0,0191032
CRIP2	0,04276847	HPGDS	0,0250937
GPER1	0,03609883	ANKRD17	0,0053701
MPLKIP	0,01037637	ATXN7	0,0050119
CRELD1	0,00106664	EPHX4	4,918E-05

Lesion specific genes

MCM2	0,01993514	ATP2B1-AS1	0,0253068
PWP1	0,00145325	LINC00174	0,003189
BAHD1	0,01852649	CTSK	0,0190896
RP11-277A4.4	0,00348542	PSIP1	0,0150903
FSIP2	0,0171942	BMP2	0,0138968
RP11-14I17.1	0,04249888	SYT7	0,0174261
MSMO1	0,02836884	CDK4	0,0061207
CAMTA2	0,04953928	BMP2KL	0,0133003
RP11-354P17.15	0,00036565	KCNAB1	0,0023454
MAFG	1,061E-05	HTR5A	0,0115432
MAMDC4	0,01678176	GRM1	8,153E-05
DCXR	0,00218273	IBA57	0,0010678
RAB11FIP5	0,01326882	PDE4D	0,0220308
AC062029.1	0,00107242	CNTN5	0,0015008
GATA2	0,00699478	GAP43	0,0492649
TMEM50A	0,00190737	GTF2IP13	0,0034676
CCNF	0,01010939	TRANK1	0,0012806
RP5-997D16.2	0,00586423	ABRACL	0,0419745
SH3BGR	0,00836034	ACAN	0,0401033
MRPL51	0,02406645	AC078842.4	0,0382794
ARHGAP35	0,02192551	ARPC1A	0,0002534
AIG1	0,03518636	FRMD4A	0,0408477
RP11-456P18.2	0,00250948	BCL11B	0,0029562
RP5-1041C10.3	0,04682902	AC011899.9	0,0212026
SUCLG2	0,00039911	PLS3	0,0012457
RPS12P26	0,01312541	PCSK2	0,0061501
RP11-512M8.3	4,0403E-05	AC010127.3	4,318E-05
MAGED1	0,00203756	RP11-314B1.2	0,0165036
GEMIN5	0,04006876	KNOP1	0,0147738
KCNE4	0,00039136	RP11-848G14.5	0,0232861
ZBTB40	0,01288309	MSTO1	0,0006496
RP11-521I2.3	6,8753E-07	CNKSR2	4,193E-05
PNP	0,02030624	DNM3-IT1	0,0031083
PIGM	0,01856031	GFRA2	0,0013439
RP11-323I15.5	1,6954E-06	TMEM62	0,0265177

Lesion specific genes

HMGB1	0,00575828	LINC00478	0,0357816
PSPH	0,04262573	SMARCA1	0,0016969
DNASE1	0,00103305	GCDH	0,0063726
QARS	0,0037099	ZNF862	0,0370315
RP11-338I21.1	0,01494115	PDE7B	3,967E-06
TRIO	0,00095869	AJAP1	0,0373776
GINS3	0,02904076	RP11-422N16.3	0,0020607
CHSY1	0,04637855	RP11-981G7.6	0,0322371
RXRA	0,00702311	HACD1	0,0110582
PDLIM7	0,04499469	GPR155	0,0293798
LINC01772	0,02078457	GSG1L	0,0157698
COG6	0,02288733	GREM2	0,0004344
ABHD4	0,02122449	RP11-161I2.1	0,0010127
PGRMC2	0,01021286	MTUS2	0,0002994
HMGB1P5	0,0439678	SHC2	0,0003457
PRTFDC1	0,02535176	CASQ1	0,0297666
RPL12	0,00416781	IRAK1BP1	2,716E-05
RP11-477H21.2	0,03020542	BUB3	0,0170795
PCDHGA7	0,00162262	GGH	0,0182491
KIF11	0,03494641	GGT1	0,0405423
ANAPC13	0,00830159	HAS3	0,0374539
HMGN3	0,02218107	ME2	0,0137146
OR7E128P	0,00767624	KPNA4	0,0211184
MPPED1	0,03994805	CD302	0,0083553
PDXK	0,0364257	EFNA5	0,0003426
PTGES3	0,00012024	NOL4	0,0027993
BCL2L2	0,04768843	AAR2	0,0176987
NEAT1	0,00046626	CNTLN	0,0197485
ATP13A4	0,00018939	SASH1	0,0095054
BCL9L	0,01434041	SYNE3	0,0059484
CSTF2T	0,00226166	CACNA1C-IT3	0,000689
RP11-443O13.3	0,02217614	NGEF	0,0107465
NAA16	0,00563285	CTNNBIP1	0,0184908
FRG1HP	0,00711132	HMGN2	0,0093103
BATF3	0,0048292	MAPK1	0,0241315

Lesion specific genes

CYP4V2	0,01274339	FNIP2	0,0331558
ARNTL2	0,01340772	SBF1	0,0019104
CPA2	0,00022325	LINC01197	0,0085868
LSMEM1	3,7917E-07	LINC02352	0,0075795
PRR14L	0,0293601	DCAF6	0,0149172
GJA4	0,00192299	SPI1	0,004656
SNORC	0,01394783	STIM2	0,0002803
LINC00092	0,03188636	UBE3B	0,0021631
SRBD1	0,01079362	BOK	0,040192
NAA50	0,00132243	KIF17	0,0077386
TMEM209	0,00728117	TMEM203	0,0346744
AL450992.2	0,00494325	RP11-781A6.1	0,0136543
UBA3	0,01157957	ZNF132	0,0142465
AGK	0,01236271	USF3	0,0297611
GPRC5A	0,00052031	ATP1A1-AS1	0,0287288
GRK2	0,01240632	RP11-388C12.8	0,0030458
NAA10	0,01600427	KYAT1	0,0123295
DPH6	0,00037539	POU6F2	0,0038991
SETD5	0,01242756	OPCML	0,0475194
CLEC18B	0,01509192	LINGO2	0,0080214
TSNAX	0,00993203	RN7SL648P	0,0146645
PCID2	0,03423825	KIRREL2	0,0479664
ZCCHC3	0,01027331	DIAPH2-AS1	0,0002006
SRGN	0,01607859	SLAIN2	0,0036483
RN7SL449P	0,02741789	WDR77	0,0410208
EEF1B2	0,01499545	ATF2	0,0039001
PER3	0,04369629	QTRT1	0,0332239
DHX34	0,0114562	CTC-563A5.2	0,0471365
ERVK13-1	0,00080659	U62631.5	0,0061359
PLK4	0,00230445	NRGN	0,0014854
PLOD3	0,01120746	NSUN6	0,0038074
EXOSC8	0,03647428	EEF1A1P3	0,0278642
ATAD3A	0,03454656	UXS1	0,0097222
MCCC2	4,8626E-06	SHB	0,0063923
SPRED3	0,01537279	HYLS1	0,001429

Lesion specific genes

ISY1	0,02009586	RP11-697N18.4	0,0057651
LY6G5C	0,01526725	TMEM97	9,209E-05
BTBD6	0,00301058	SUSD4	4,928E-05
FOXP4	0,04673555	EGLN2	0,0018012
NDUFB10	0,00463016	TRIM28	0,005723
CCDC154	0,02642798	FKBP15	0,0466763
R3HCC1L	0,01262523	NTNG1	0,0001149
ZNF337-AS1	0,03701245	RP1-68D18.3	2,032E-05
LATS2	0,00993203	TMEM87A	0,0047243
GON7	0,00896067	SCG3	0,004363
WDR11	0,0494381	RP11-96H19.1	0,0295999
ZNF32-AS2	0,02509841	GABRB3	0,0008931
NUDT9	0,00155948	COL6A4P2	0,0062613
RP11-274H2.2	0,03111782	GIPC3	0,0488094
MRPL3	0,01554962	TAF3	0,0170943
POC1B-AS1	0,00181762	FLRT3	9,414E-05
SLC5A6	0,02303177	RP11-343K8.3	0,0004468
SUMO2	0,01729412	ADAMTS3	0,0090156
HIST1H2BG	0,03153723	PCDHB12	0,0496491
IL12RB2	0,01255374	CLSPN	0,0031304
SEPSECS	0,02810861	SMG7	0,0056061
BCORL1	0,02575993	RP11-494K3.2	0,0298783
FHOD1	0,00579095	LSAMP-AS1	0,0109734
TFDP1	0,01167229	SYPL1	0,0344783
FAM173B	0,00178025	UNC80	0,0170061
SAMD14	0,03414547	MCUR1	0,0276841
NPIPB2	0,00544873	PPA2	0,016671
WIPI1	0,03254469	MIPOL1	0,0074475
PDZD8	0,01772414	FAM53B	0,0134869
ATG4C	0,03454656	USP36	0,0322437
ZDHHC5	0,02006359	GABRG2	0,0116329
AJM1	0,02610109	FAM78B	0,0005862
CAV1	0,0375722	IGBP1	0,0489478
PLAGL2	0,00766057	STXBP1	0,0024815
DBN1	0,02892006	ADM	0,0012724

Lesion specific genes

ARMCX6	0,04132774	TBRG4	0,0089656
SLFNL1	0,02117481	STX1B	0,0435338
LINC02210	0,00168255	ZNF620	0,0145627
ZNF185	0,04388593	RP11-466A19.1	0,0056981
ZNF587	0,02700233	MFAP1	0,028195
HIST4H4	0,01219312	GABBR2	0,0006052
FBXO4	0,03296692	KCND2	0,0006396
RP5-1021I20.5	0,00039899	RP11-395G23.3	0,0349061
WSB2	0,02244789	PIP5K1B	3,003E-05
RP1-104O17.3	0,03654276	SLC24A3	0,0414361
ZMIZ1	0,03670632	LINC00599	0,0423372
RNF125	0,02034454	AC073342.1	0,0003461
RP11-119F7.5	0,02344108	UBE3A	0,0249584
EIF4A2	0,00601992	C11orf96	0,0270602
MALAT1	0,02290442	RNF114	0,0025585
MARS	0,02855203	RAD54L2	0,0008631
PPIE	0,02177282	KCNIP2	0,0014279
LOC100130950	0,01482367	KLF13	0,0313996
ARRDC1	0,04005095	RNU6-142P	0,0141544
OAZ1	0,04376748	PFKFB4	9,939E-05
WNT4	0,032714	KRT8P42	0,0029532
TCF7	0,00598255	HIST2H2AB	0,0004399
GKAP1	0,01540299	WDYHV1	0,0220308
RN7SL128P	0,01491007	C13orf46	0,0079499
FICD	0,02799422	OLFM3	0,0123455
RN7SL262P	0,02096539	SEC61B	0,0475937
LINC00284	0,04470767	ANKMY2	0,0171996
PDIA4	0,03507747	PLEKHA5	0,00097
AK3P3	0,00277849	IGSF22	0,0278583
DMAC1	0,00037467	ETV1	0,0002131
LOC101928445	0,01058548	SPTSSB	0,0023096
SELENBP1	0,03810216	RP11-540B6.6	0,0007538
KCTD9P1	0,03409197	PPARGC1B	0,0023851
ZNF259P1	0,00519795	NME1	0,0391305
A2MP1	0,04286163	MUM1	0,0492424

Lesion specific genes

RNF44	0,03553961	RP11-384P7.7	0,0150795
NPTXR	0,0210298	PCSK7	0,0241212
PINK1-AS	0,0424859	HGS	0,0156224
AC104135.4	0,00748941	LNX1	0,0194499
RANGAP1	0,01400724	LOC101929095	0,016671
CNOT6L	0,04467104	ERC1	0,0002896
UTP20	0,02851163	NHLRC1	0,0146207
MPL	0,03864109	OR7E7P	0,0140303
C9orf78	0,04513756	COMT	0,025228
TAS2R43	0,0204799	C1orf43	0,0015684
IGF2BP3	0,03459797	USP27X	0,0102453
MTMR1	0,02069271	B3GNT2	0,008572
TSC22D1	0,00435861	PPP3CB-AS1	0,0230238
AC008746.12	0,00178025	SCN3B	0,0037719
SHC1	0,03541094	DRD1	6,593E-05
TANGO2	0,00398742	DONSON	0,0038343
GAREM2	0,02911544	ZFP92	0,0105969
OGDH	0,03975477	OTUD1	0,0490291
LINC00323	0,0099152	CCDC183	0,0452942
PLIN2	0,03348568	TMEM130	0,0251422
DENND1A	0,00499807	RP4-671G15.2	0,025903
QPRT	0,03040443	ZSCAN18	0,0007337
RP11-755F10.3	0,00053537	APOBEC4	0,0068268
LDHB	0,02642798	PNKP	0,0201186
PLD6	0,00106266	RBFOX3	0,0005544
IGFL4	0,03035445	KCNQ5-IT1	1,204E-10
ANKRD10-IT1	0,02150664	MID1IP1	0,0278295
NDUFC1	0,00047834	SMDT1	0,0065647
NBR1	0,04066064	RP11-12J10.3	0,0001855
IMMT	0,01337537	AFAP1-AS1	0,0229239
NAALADL2	0,0432237	CHID1	0,0301763
AURKA	0,04247075	LINC02282	0,0002564
TRAF2	0,02064115	SMPD4	0,044183
MIR29B1	0,00473797	RP11-223P11.3	0,0132162
USP17L2	0,04647942	GRM8	0,003066

Lesion specific genes

TMEM79	0,0228914	RAB30-AS1	0,0379814
ABCC2	0,00207196	SEPT5	0,0028418
TTC12	0,02191206	DACH1	0,0006392
SPATA12	0,01436499	MPP7	0,0363682
OLMALINC	0,01243494	SLC2A13	0,0186561
TMEM185B	0,01599468	LINC01792	0,0075043
KB-1507C5.4	0,01431866	RASL10B	0,0323044
TRPM2	0,02582118	DICER1	0,0353155
MVD	0,04420093	MAPKAP1	0,0112513
IRS2	0,04697425	CYFIP1	0,0346741
PITPNM2	0,00419855	UBQLN4	0,0137884
MRPL11	0,0153946	BNIP3P25	0,0121452
MPDU1	0,00701025	LMLN	0,028195
TMEM5	0,00560649	FRRS1L	0,0163262
NLGN3	0,04076783	RP11-216L13.19	0,0419133
ASAP1-IT2	0,02370822	MARK1	0,0025916
RP11-874J12.4	0,04491265	MRGBP	8,639E-05
PIP5K1C	0,00273696	PAK6	0,0233564
ERGIC3	0,0271842	RBM4	0,0009674
C8orf59	0,04652931	MAGI2-AS1	0,0048475
TNFRSF25	0,04835088	SCIN	0,0049488
ZBTB24	0,03080552	RP11-452L6.1	0,0425219
DOCK6	0,04594012	GNG5	0,0115247
RP1-223B1.1	0,00309248	SNHG11	0,0229338
RWDD2B	0,02922317	LOC155060	0,0066202
SLC35G6	0,00384295	UHRF2	0,0147101
COL9A1	0,03191349	ZFP69B	0,0136141
BPGM	0,00469543	CCDC122	0,0427934
HMOX1	0,00273288	GCM2	0,024521
SERPINB8	0,02454769	RASGRP2	0,0036973
NXN	0,00233365	LOC101928053	0,0075982
PPIH	0,01153832	RCOR3	0,0087532
FLCN	0,00203953	RP11-61J19.4	0,004642
ROMO1	0,04214339	CDH2	0,0001197
CA4	0,0248403	KRAS	0,0159433

Lesion specific genes

LOC100507291	0,00219309	TENM2	0,0001863
RBM14	1,0354E-05	LOC440040	0,0019545
SPTY2D1	0,01797824	CABCOCO1	0,0387174
ENTPD1-AS1	0,0076638	POMZP3	0,0128167
MEX3A	0,03599562	MGAT5B	0,0373108
KRT8P1	0,03575818	TOM1L2	0,04235
RN7SL381P	0,0020618	MNDA	0,0261797
RP11-701P16.2	0,00874787	RPS19BP1	0,0115432
ABHD13	0,00097386	RP11-14N4.1	0,0242234
STX18	0,0030196	CPNE2	0,0068475
MPRIP-AS1	0,02567394	GUCY1B3	0,0012875
NIF3L1	0,00682809	FSTL4	6,878E-05
RFC1	0,02385831	TMEM171	0,0189405
ANKRD44	0,02591422	PAXIP1	0,0120291
LMBR1L	0,02337226	RP11-2E11.9	0,0238911
SLC25A20	0,00659502	PDXDC2P	0,0245386
RSBN1	0,04513898	RORB	9,267E-05
EIF4EBP1	0,00981468	SLC9A7	0,0008236
ARSD	0,03494307	VAPB	0,0334051
TIMELESS	0,01363387	SNAP25	0,0062656
ZNF396	0,0061583	RP11-1140I5.1	0,006838
TDRD6	0,00729912	IFT22	0,0040883
RP11-158H5.7	0,03459823	RP11-43D2.2	0,0219639
P4HA1	0,00244775	FN3KRP	0,0157101
GPR17	0,0097941	ITPKA	0,01501
COL4A1	0,00761638	GPATCH4	0,0102192
OSBPL2	0,00349774	B4GALT6	0,0038225
MNT	0,00106266	PCDHB4	0,0221886
PURB	0,00509031	ZNF792	0,0212443
RP11-714G18.1	0,00090631	TTC39C	0,040192
GTF2I	0,04065539	CLVS2	2,745E-07
IGSF9B	0,0385436	KCNC3	0,0285949
KCNJ2-AS1	0,01290298	USP25	0,003152
ABCB6	0,00173916	LOC101927751	0,0079499
AC069282.6	0,00255992	AC004593.3	0,0066812

Lesion specific genes

SPATA13-AS1	0,03351158	PARP6	0,0050278
AZI2	0,00351406	GAMT	0,0071378
NFXL1	0,04591011	PFKFB3	0,0246412
KPTN	0,01483435	CLCN7	0,0321104
PSMB3	0,00639275	JPH4	0,0113562
DEDD2	0,00782788	CABLES2	0,0241201
TOLLIP	0,02328627	MAN2A1	0,0403635
PLVAP	0,01493603	RP11-509E10.1	0,0001058
RP11-138A9.2	0,00358025	CSNK1D	0,0183823
RP11-1277A3.1	0,03691765	PTPRT	0,0450061
AP000892.4	0,00148084	RALGDS	0,0085475
PIPOX	0,0080918	SCARNA6	0,0007934
CACNA1G	0,03401718	ARFGAP2	0,0098312
VGLL4	0,00771007	PLEKHH2	0,0201863
TMEM81	0,0050386	RP11-849F2.5	0,007379
UBOX5-AS1	0,04649377	CXXC5	0,0317172
PVR	1,5258E-06	LRRTM1	6,082E-08
PLOD1	1,9623E-05	VPS13A	0,0027398
RYR3	0,00489563	LHPP	0,0258802
VAMP2	0,02116065	OSBPL6	4,673E-06
MEF2D	0,03391706	RXFP1	0,0327231
PANX1	0,01895687	NIPAL2	4,756E-06
AP000692.9	0,00499024	CUX2	0,025903
TICAM1	0,01341255	MIR421	0,0375487
ZBTB34	0,0425902	ZBED8	0,0041242
PTRHD1	0,01800666	LCNL1	0,0172975
FLVCR1	0,00821109	SKIV2L	0,0213701
FAM201A	0,02587897	BMPER	0,0492304
LYRM2	0,04327469	C16orf96	0,0144607
RP11-493E12.2	0,03752112	ADA	0,0278642
PKD2	0,02926919	EPHX2	0,0264395
LINC00653	0,0258398	ZMAT3	0,0140811
RP11-324I22.4	0,02997776	ATF7IP	0,0007596
PTOV1	0,01000447	GPR26	0,0287924
PTPN11	0,03018657	LINC01561	0,0441352

Lesion specific genes

PSMC2	0,0067899	RP11-214K3.22	0,0118177
LINC00173	0,00500704	DUOX1	0,0004045
RP11-378A13.1	0,00449258	AC004945.1	0,036964
PM20D2	0,00219485	SAP30L-AS1	0,0312639
C11orf98P1	0,04634405	SSPO	0,0004399
USP49	0,02195851	ACYP1	0,013379
RP6-109B7.2	0,00678644	ARPC2	0,0018389
RN7SL396P	0,00631408	P2RY13	8,415E-05
NOP2	0,0078953	OGT	0,0247632
SDHAF3	0,00830071	RPL3P6	0,020101
LINC02593	0,00019403	CBFB	0,0048111
PSPN	0,00135954	EBAG9	0,0315977
MTMR8	0,00438122	COMMD4	0,0233635
PPIAP22	0,00094292	ZNF276	0,0125941
APLNR	0,04057964	RP4-561L24.3	0,0166914
MPV17L	0,01601282	ACTL6B	0,0369818
NUDT18	0,03632991	STXBP5L	0,0010842
PRSS35	0,00351406	NSF	0,0184772
RP11-1129I3.1	2,2049E-07	EBF4	0,0066522
ZNF410	0,00551331	ANKRD39	0,0428317
LINC00963	0,0468163	HTR1F	0,0098475
C4orf36	0,00182733	CDCA3	0,0477951
HILS1	0,00073326	XXbac-B476C20.14	0,0222597
LEPROT	0,02351092	PLCB1	4,871E-06
MRRF	0,02718977	MTG2	0,0464924
ZYX	0,04929127	LOXL2	0,0001037
NCAPG2	0,026915	RASGRP1	0,0035909
KCNMB1	2,1913E-05	NDUFAF2	0,0239002
ITGAV	0,00256012	GUCY1A2	0,0011293
MMACHC	0,0204378	CHST9	0,0079631
CTD-2201I18.1	0,00568712	ZBTB33	0,0001113
C11orf95	0,03808907	GOLGA8H	0,0231275
SRGAP2	0,04356124	MTURN	0,0232844
SMN2	0,00488221	MYO19	0,0171214
RP11-110G21.1	0,00054352	HTR5A-AS1	0,0011952

Lesion specific genes

NSL1	0,00805229	FGF9	0,0049196
AC016831.7	0,01179488	LINC02060	0,0013367
P2RY12	1,2661E-05	CACNA1A	0,023217
KANSL1	0,02498783	ABI1	0,0116239
GCNT7	0,02317015	SLC1A4	0,0413247
KIAA2026	0,03865489	RP11-691H4.4	0,0062912
TNFAIP8L1	0,02041158	GNAQ	0,0351378
GBA	0,04332551	CKAP2	8,326E-05
JOSD1	0,03352705	CACNG3	0,0025592
UCKL1-AS1	0,00029393	TMEM173	0,0214272
CEP89	0,00747906	HNRNPM	0,0115432
ZNF761	0,04638481	ZNF14	0,0094987
CHAC1	2,1913E-05	CALCRL	0,0400168
INTS3	0,02743809	MIR548V	0,0012991
ZNF558	0,0243418	RP5-850O15.3	0,0116377
FAM227B	0,00050422	MICAL3	0,009055
RIN1	0,04626585	PCDH8	0,0281654
ZDHHC18	0,01002868	PAICSP4	0,0003422
ST3GAL2	0,02383081	NOL6	0,0110408
RMI1	0,0256513	STEAP2	7,211E-07
CLDN15	0,00242308	TRIM44	0,0039235
SOCS7	0,0006294	RYR2	0,0035743
RP1-168P16.3	0,02349519	SERHL2	0,025264
MYH15	0,00223206	LINC01422	0,0025717
LINC01141	0,00088996	PCSK6-AS1	0,0402594
ATP1A2	0,02446563	FAT3	0,0001151
GRASP	0,00473797	ZNF273	0,000508
AC005519.4	0,00146876	RP11-529F4.1	0,006714
EPN3	0,00757313	WSCD2	0,0012103
JMJD6	1,2734E-11	GABRA4	0,00012
ARRDC3	0,01804637	MACROD2-AS1	0,0125173
DDIT3	0,03651087	OPRM1	0,0197143
HPCAL1	0,00168255	AC159540.1	0,0247496
RP5-894A10.2	0,00108043	GNB5	0,010208
IFIT1	0,01740832	RSAD2	0,0129385

Lesion specific genes

HSPA5	0,0140185	CRTC3	0,0208341
SYS1	0,04166604	CDH23	0,0070307
ZNF449	0,04186976	EXOG	0,0197485
MGME1	0,00282379	RGS6	0,0199898
PFDN6	0,01329944	RNF144A-AS1	0,0015688
ANAPC16	0,02182246	RNF121	0,0296473
RN7SL566P	0,01641771	MEG3	0,0001515
LOC105375666	0,03949352	DYRK1A	0,0002973
RP11-754B17.1	0,01447259	HTRA2	0,0401677
RP11-16P6.1	2,0913E-05	TMEM30A	0,0312884
PRR34-AS1	0,01772196	ETV5	0,0001654
EID3	0,02444528	PIP	0,0043149
RP11-301L8.2	0,01511509	GSTO1	0,0001674
FGFR1OP	0,00298318	CORO7	0,0274522
SNHG10	0,04051835	MAD2L1BP	0,0106006
RP11-367H1.1	0,00660471	PCP4	0,000962
HSF1	0,00460658	CDC42BPB	0,0136496
ADSL	0,01483404	RIMS2	0,0065748
POLR3H	0,03003876	RP11-556G22.1	0,0019661
ABHD11	0,03916725	NPTN	0,0001402
CTD-3131K8.2	0,03207214	UPP1	0,0090334
LINC01003	2,3282E-05	SCN8A	0,0011066
NMI	0,02619584	RNF149	0,0194168
RNASEH2A	0,00908016	FDXR	0,0168253
REL	0,03745657	PRKAB1	0,0256878
PEX10	0,0168502	LINC01748	0,0420266
CTC-360G5.9	0,01734135	KSR2	0,0157852
ALS2CL	0,01086871	L3MBTL3	0,0488315
SCAND2P	0,01561799	SNRPB	0,0355517
C6orf106	0,00174179	DLGAP2	0,0005876
HSP90AB3P	3,3914E-06	TAPT1	0,0187228
PEX11A	0,02490853	SS18L1	0,0379697
LOC102724017	0,04065539	COL10A1	0,0250509
TRAM2-AS1	0,00437579	NAA25	0,0043015
LRP4	0,02454999	ELL2	0,0440985

Lesion specific genes

ABLIM3	0,0116566
Z69666.2	0,03470531
PKMP3	0,0011146
GTF2IP14	0,0094216
KLF10	0,02344108
HGSNAT	0,00540098
ATIC	0,01954108
STRN	0,0293601
SULT1B1	0,00115171
NCOA4	0,04772128
POC1B	0,02605876
SERTAD4-AS1	0,0322673
RPL9P29	0,01095178
SLC25A5-AS1	0,01073076
ATP5G1	0,02801721
PPWD1	0,04022588
PTCH2	0,04814379
GOLGA8B	0,00226738
RP11-799D4.4	0,00064763

LOC105370941	0,0279965
RANP4	0,000639
GPR87	0,0063283
RIPOR2	0,0301725
ADPRHL2	0,011486
RPF1	0,0403359
CEP44	0,0380649
PRR26	0,0063335
MGEA5	0,0081592
LRCH3	0,0405366
LIG3	0,0415452
ECI1	0,0219344
SLC4A11	0,0064647
MYH7B	0,0067206
UNC45A	0,0180222
GIT2	0,0216292
SDCCAG3	0,0152282
LRRC3B	0,0255775
FANCB	0,0328568
LMBR1	0,0338172
RPL5P14	0,0181529
TMCC1-AS1	0,0039339
PPP1R12A-AS1	0,0136631
FLRT1	0,0106015
SNX17	0,0069624
SPOCD1	0,0094816
CDHR2	0,0442045
ELOA-AS1	0,0030934
HBA1	0,023408
MPST	0,0173514
LOC554206	0,0268247
STOX1	0,0008292
NAP1L2	0,0251273
EPB41L4B	0,0008206
XK	0,032714

Lesion specific genes

IGLV5-52	0,0437158
PHYHIP	0,021307
SLC12A8	0,0159821
LRRN1	7,335E-06
IGSF3	0,0018543
DUS2	0,017197
PTCD3	0,0220641
TEAD1	0,0268459
MAPKAPK2	0,0406814
DIP2A	0,0015271
PRKCB	3,236E-05
RELT	0,0202013
ROCK2	0,0121805
PPP1R15B	0,0399573
SMG1P6	0,000342
HES1	0,0192525
LINC01138	0,0173514
RBM17	0,0178357
SUPT4H1	0,0167259
ARHGEF26-AS1	0,0143035
ANO5	0,0300775
RP11-378J18.8	0,0314343
DNAJC15	0,0195215
EXOC3	0,0087208
SMUG1	0,0250357
AP3M2	0,0136794
ZNF385B	5,475E-07
ETNK2	0,0097551
KLF3-AS1	3,48E-05
ING5	0,0029675
KIAA1211L	0,0005927
AP006222.2	3,106E-06
OPHN1	0,0292817
MARF1	0,0285477
MYO15B	0,0171373

Lesion specific genes

KIAA1324	0,0216948
PLCL2	6,831E-05
WDFY2	0,003366
RP5-940J5.6	0,0381636
TIMM22	0,0172936
TOMM34	2,98E-05
CHST11	0,0259889
POLR2J4	0,0085784
HMGB1P21	0,0295374
B3GALT5	0,0164342
TSPAN7	0,0077999
MAPK10	0,0014232
TM6SF1	0,0213701
COX10	0,0015688
DNAJA3	0,0306941
FAM234A	0,0419128
KCNQ5	3,487E-05
KRT222	0,0187003
TPBG	5,241E-06
CDHR5	0,0205829
TATDN2P2	0,0286929
LONRF2	0,0156021
NRG4	0,0267338
PIGF	0,0357867
ATP6V1C1	0,0079053
KCNA4	0,0028163
COQ10B	0,0293637
NMNAT2	0,0003327
SH3PXD2A	0,0002191
MILR1	0,0099642
MICALL1	0,0003029
LOC101927972	0,0005787
MTCO2P12	0,0360118
KCNJ6	0,0030266
STRIP1	0,0163782

Lesion specific genes

UPP2	0,0311778
DOK4	0,0492534
GPR158	0,0011053
KCNIP4-IT1	0,0001412
SLC4A5	0,0009177
CELF5	0,0328148
GRIP1	0,0142465
CYP2T1P	0,0003739
JPH2	0,0241212
ATP2B2	0,0012146
RGS20	0,0158192
RP11-550F7.1	0,0087478
SLC51B	0,0489878
RP11-171G2.1	0,0195555
RER1	0,0034386
ELAVL2	0,0480129
CSMD3	0,0061967
DLGAP1	0,0031472
DLGAP1-AS4	0,0001313
LAMTOR3	0,0487609
GTF2H5	0,0319639
ISCU	0,0492373
FAM131C	0,0004682
ANKRD20A5P	5,289E-05
VAX1	0,0095779
DOPEY2	0,0205749
RP11-798M19.6	0,021587
RP11-211A18.2	0,0021793
RP11-35G9.5	0,0175443
SAT2	0,0255259
RMDN3	0,0457551
ACTBP7	0,0002447
KIAA0753	0,0002726
CLIP2	0,0298093
PPCDC	0,0260288

Lesion specific genes

NT5E	0,0072352
RP11-438B23.2	0,0289139
IL18	0,0474957
VPS16	0,0027082
FOXRED1	0,0370315
CEP164	0,0338113
TUSC7	0,0073677
CCDC144A	0,0023242
WIF1	9,366E-06
TMOD2	0,0030098
SLC44A5	0,0420366
FAM153C	0,0126896
FBXL5	0,0282032
METTL16	0,0276382
KAT5	0,0285949
CSRNP1	0,045094
NRSN1	4,317E-05
GRIN2A	0,0089687
MAN2C1	0,0034491
CEP72	0,0219639
SNRNP48	0,0058166
NRXN1	0,0106352
AGBL1	0,0069155
NELL2	0,0150977
ARHGAP10	0,0309688
DPY19L2P2	0,0004767
VCP	0,0005055
THEMIS2	0,0018787
GSK3B	0,0496491
GPD1L	0,0016781
KLHDC2	0,0410057
RP11-293M10.6	1,431E-06
PKP2	8,822E-07
TMEM205	0,0132989
USP51	0,0385905

UPF1	0,0315027
CTDP1	0,0242104
ACOX3	0,0229239
INHCAP	0,0003037
UBE2QL1	0,0067551
RP5-1039K5.16	0,0001908
TRPV1	1,377E-07
CC2D1B	0,0425075
PLXNA4	0,003197
SYN2	0,0060205
URB2	0,0479182
AC006160.5	0,0014217
EIF2AK2	0,0038075
LMO7	0,0011919
CITED2	0,0075901
VSTM2A-OT1	0,0413839
GRAMD1B	0,0026917
FOXP2	7,216E-05
ASH1L-AS1	0,0014835
ATP5J2	0,0193999
BRCA2	0,037727
TUNAR	0,0004468
SCAMP4	0,0154846
MIER2	0,0328992
FAM171B	0,0213803
TFB1M	0,0150166
MOV10L1	0,0470797
NAGLU	0,0095705
SERTAD4	0,0096765
CDON	0,0312309
SH3YL1	0,0148888
NDST3	0,0016421
FAM19A2	0,0189198
CAPN2	0,0247319
TRIM17	0,0219437

Lesion specific genes

ZSCAN21	0,0101273
PRC1	0,0285205
RP11-449P15.2	0,0439064
MYT1L	0,0050899
OSBPL7	0,0367409
UFD1	0,0188177
ARMC9	0,0023152
SLITRK3	0,0232448
ITPKB	0,0078801
ZBED9	0,0017847
TTC39B	0,0090167
RP11-342M3.5	0,0139147
IPCEF1	0,0164493
C2orf72	0,0032841
BIN2	0,0035252
D2HGDH	0,0287864
C2orf80	0,0036881
TOM1L1	0,0224585
LINC00869	0,0083318
RP11-290D2.3	0,0014326
CKMT1A	0,0273767
EIF4ENIF1	0,0184747
RPL7L1P12	0,0113342
TAZ	0,0009946
WASH5P	0,016019
DIO2	0,0057857
CRYZP1	0,038001
TAF11	0,0002306
UNC13A	0,0035278
TARBP2	0,0236757
TUBGCP4	0,008263
PLS3-AS1	0,0261195
NCOA1	0,0046752
GCN1	0,0054289
HIPK4	0,0464426

Lesion specific genes

SYF2	0,0338374
CEP192	0,0036493
HHLA3	0,0124362
FAM66C	0,0229197
ZNF688	0,0384428
CACNB2	1,785E-06
PDE10A	0,0028209
UBE2V1P1	0,0329378
METTL24	0,0076929
RHOJ	0,00305
VSTM2A	0,0008062
INTS2	0,0290508
CCZ1	0,0017879
SYBU	0,0299666
PDE4DIP	0,0158609
MCF2	0,0356108
ZNF263	0,0113599
MED12L	9,987E-06
FBXL16	0,0275149
RP11-263K4.1	0,0094538
SHPK	0,0027552
RNH1	0,0017263
CDH7	8,924E-05
FGF12-AS1	0,0004525
SHANK2	0,0038225
FGF13	0,0022375
CASKIN2	0,0410952
TAB3	0,0346991
ZNF252P-AS1	0,0489898
ZNF496	0,038963
MAGEE2	6,153E-06
RNF10	0,0013992
ZNF567	0,0041421
NCBP3	0,0297402
RP1-97G4.1	0,0002593

Lesion specific genes

NREP	0,0001396
SUMO2P8	0,0001915
RP11-327O17.2	0,0076218
GABRA5	0,0069812
CELSR2	0,0475612
TLK2P1	0,0153116
CIAPIN1	0,0472419
NANOGP4	0,0172058
HILPDA	0,0237517
HOOK1	0,0008639
NEXMIF	0,001428
KCNQ3	0,0150367
SOCS5	0,02042
DLG5	0,0162204
TFEB	0,0072484
AF131215.9	0,0058906
PROX2	0,0022192
RPAP2	0,0471365
MIA3	0,0111227
RP11-1023L17.2	3,918E-05
HARS2	0,0011257
UBE2V1	0,0049201
RP11-999E24.3	0,0044402
CNNM3	0,0150988
TSTD2	0,0181679
ANO3	0,000667
SFT2D2	0,0384321
JMJD7-PLA2G4B	0,0238453
IFRD2	0,0095812
PYCR2	0,0432484
ZNF391	0,0003222
FAM20C	0,009959
WRB	0,0423372
NDUFS3	0,0218591
SAMD4B	0,0064355

Lesion specific genes

MAP2K3	0,0122262
SMAP2	0,0135203
RFC5	0,001565
BRAF	0,0204944
BABAM2	0,0176987
HHATL-AS1	0,038788
PASK	0,0005792
ZNF776	0,0104275
ARFGAP1	0,0106967
CTD-2248H3.1	0,0340075
COL12A1	0,0107921
CHST3	0,014234
TRIM62	0,000272
RP11-823P9.3	0,0167067
MAP3K9	0,0222597
NYAP2	0,0026021
NCR3LG1	0,0017329
C15orf61	7,885E-05
HECW1	0,0033227
HECW1-IT1	1,127E-05
ZNF519	0,0029446
ZNF304	0,0446428
DBNL	0,0312884
KANK1	0,0410057
ZNRF2P2	0,0134305
RNF122	0,0296413
CXCL14	6,763E-05
C17orf76-AS1	0,0380269
AC013394.2	0,018141
TPD52L2	0,0327231
PTK2B	0,0118933
NUDT10	0,0049504
FBXW8	0,0235035
DUSP22	0,0011439
CALB1	0,0042899

Lesion specific genes

L3MBTL2	0,0002593
MCRS1	0,0251942
ST6GALNAC5	5,887E-05
DIRC2	0,021587
GPR83	0,0153497
TCIM	0,0226981
DIAPH2	5,689E-06
LAMP1	0,0036483
ARPP21	7,277E-05
RGS3	0,0239549
UAP1L1	0,0011831
SCOC	0,0042957
HAGHL	0,0153133
ATP11A	0,0153646
SLC15A4	0,000206
ALDH4A1	0,0225186
SPRYD4	0,0388597
TRNAU1AP	5,005E-05
RASD2	1,622E-05
JAKMIP2	0,0477535
C6orf203	0,0468161
PRKG2	0,0185449
CTD-2555O16.2	0,0321191
CTC-471F3.5	0,0093396
ZNF774	0,0086831
PCP4L1	9,093E-05
PLXNB1	0,0022761
RP11-90P13.1	0,0399573
KNSTRN	0,0002924
RP11-266L9.6	0,0409066
SNAP25-AS1	0,0170832
PI4K2A	0,0155269
LY86	0,0384806
RP1-122P22.2	0,0036766
NACC2	0,0171214

Lesion specific genes

EPHA5	0,0003871
EMG1	0,0291614
PCDHB11	0,0183389
DCLRE1B	0,006113
TP53INP2	0,0364194
AC092338.5	0,000877
RP11-567G24.1	0,0134595
OLFM1	0,0047605
RBM19	0,0116315
PITRM1-AS1	0,002099
DBF4	0,0076123
EFNB3	0,0328148
LOC105374516	0,0109775
FAM171A1	0,0120468
RP4-742J24.2	0,036074
PCLO	0,0004925
LINC01725	0,0249225
ASPH	0,0472774
PGBD1	0,0094926
SYNGR2	0,0369818
CCNL2	7,015E-05
FBXW12	0,0031299
RP11-627G23.1	0,0064012
TSPYL2	0,0001615
RP11-441F2.5	0,0409634
CHL1	0,0030888
PMS2CL	0,0017063
NAA20	0,0351309
MED11	0,0320879
EFNA1	0,0049303
SLC1A2	0,0029397
SMIM7	0,0205829
TMEM35A	0,0025009
GABRB2	0,0114344
SLIT3	0,0229625

Lesion specific genes

EGLN1	0,0024469
LOC148413	0,0082466
GRIN2B	0,0049984
PHC2	0,0087748
SLC25A29	0,0185449
SPHK1	0,00994
RP11-146D12.2	0,0158932
FAM222A-AS1	0,0011952
RP11-214N15.5	0,0281674
AC073342.12	8,154E-08
PAK3	0,0153497
CHCHD1	0,0329676
MRC2	0,0113342
CDC42	0,0238093
SNX2	0,0242726
CHRM3-AS2	0,0032334
RP11-967K21.1	0,0005753
MAP7D2	0,0114192
ZNF134	0,0108901
ANKRD36BP2	0,0026216
CD9	0,0405401
USP53	0,0021323
CAMK1G	0,0004214
DOK6	0,0270497
TMEM129	0,0188367
QPCT	0,010079
SUN2	0,011076
CELF3	0,0088454
MLLT3	0,0279301
IGSF5	0,0009843
SHC3	0,0225874
SMARCE1P1	0,0345235
RP11-272L13.4	0,0079499
RP11-206L10.2	0,0382937
AC009961.3	0,0098374

Lesion specific genes

SEH1L	0,0413247
AMIGO1	0,042948
OSGIN2	0,0042091
RP11-385D13.3	0,0002598
NEB	0,0079917
CXorf38	0,0037607
FBXL2	0,0439891
IVD	0,0050387
LOC286083	0,0167067
MVP	0,0005232
AC006262.6	0,0004205
L1CAM	0,0331498
GNL3L	0,0343135
CACNA1D	0,0115432
PRKAA2	0,0218752
RGS5	0,0245579
PRKAR2B	3,586E-05
SLC8A1-AS1	0,0043409
PCDHB7	0,0134563
SEMA5B	0,0115207
TYW3	0,0043409
LURAP1	0,004823
DDX28	0,0381094
LRRTM4	0,0001853
CACNB4	0,0026046
LINC00710	0,0079883
LINC00674	0,0323044
PTGDS	0,0113599
INPP4B	0,0102555
IFI6	0,0218497
RP11-452L6.5	0,0003336
HIVEP2	0,013379
RP11-713C5.1	0,0242707
SNRPA1	0,0167701
CERS5	0,0484657

Lesion specific genes

ANOS1	0,0069107
HMGB1P24	0,0403941
TBC1D30	0,0002234
STIL	0,0487798
DHPS	0,001587
AC131971.1	0,0471007
SLITRK5	3,517E-06
RP11-797A18.6	0,0249674
MUL1	0,0124166
ST20	0,0095098
FUT9	0,0106614
SLC4A9	0,0242831
OGFOD3	0,0316849
CYP27A1	0,0006773
CAMK2A	0,0249584
GRM5	0,0033171
RP11-490E15.2	0,0047142
RPS12	0,0323757
TMEM198B	0,0138718
ABCB10	0,0151341
OPRD1	0,0268644
LGI1	0,0025717
CAPRIN2	0,0085116
PURG	0,0130485
STRBP	0,0006764
PSMA7	2,267E-05
CNGA4	0,0384739
ACAA1	0,003399
TM2D2	0,0046512
PPM1L	0,0412701
SPATA20	0,0414357
PSMB5	0,000245
ABCB4	0,0135203
RP11-544A12.4	0,0117815
CTDSP1	0,0349574

Lesion specific genes

CDK5R1	0,0440587
CHD4	0,0051507
ACAD10	0,004906
ABHD12B	0,003197
TSEN34	0,0126896
SMIM14	0,0499867
RP11-586K2.1	0,0085025
SLC41A2	0,0016199
KHSRP	0,0063501
KCNIP4	0,0159847
CAMK1D	9,994E-05
KMT2A	0,0339497
PKN1	0,0447144
SPON2	0,0279307
ARL3	0,0399573
BPHL	0,0003786
ERCC6	0,0073664
THOC6	0,0214259
LRRN3	0,0075981
PTPRO	0,0001744
RP11-573D15.9	0,0401668
NSMCE3	0,0330497
LMO3	0,0042957
XKR6	0,0003225
RP11-490G2.2	0,0027485
MLH3	0,0015116
SRSF9	0,0232894
TMOD1	0,0002938
INSR	0,0167153
PHF13	3,918E-05
RP11-856M7.2	0,0225577
AC007787.2	0,0175597
RASAL2	0,0002173
SYNJ1	0,0129967
NIT2	0,0369384

Lesion specific genes

ME3	0,0033794
JAK1	0,0150977
GPD2	0,0270855
RP11-346C16.1	0,0025592
MYCBP	0,0151023
QSER1	0,0011847
NRCAM	0,005392
AF131215.2	0,0258067
LYRM4	0,0021214
TIMM13	0,0284385
HSD17B12	0,0201288
EFNA3	0,042948
TBC1D2	0,0031483
HS6ST3	0,0194732
EIF3K	0,0305948
LINC01551	0,0009003
PLB1	0,0020373
RP11-680F8.3	0,0001323
ENC1	0,0408838
HPS5	0,0004933
SLFN5	0,0282365
ANKRD34A	0,0012816
MIOS	0,0488652
SV2C	0,0085503
GMDS	7,906E-05
SPARC	0,0073957
RP11-90L20.2	0,0125427
ZNF662	0,0484226
F3	0,0455842
SEPHS2	0,0043896
PRICKLE1	0,0014217
FOXO3	0,0202013
ZBTB5	0,0089377
BRD3OS	0,0238768
KRT18P57	0,0040418

Lesion specific genes

CARMIL2	0,0438546
DLX1	0,0014723
RFX7	0,0477769
TESC	0,0089704
AC138035.2	0,0107208
EOGT	0,0117148
STK38	0,0228459
AC073343.1	0,0308205
TOMM7	0,0157698
EVA1C	0,0156241
SNHG14	0,0398343
PLXDC1	0,0012344
RUNDC3A	0,0201288
TRHDE-AS1	0,0005316
AC007386.4	0,0023161
SNAP91	0,0056061
RP11-115H15.1	0,010079
DAPK1	5,289E-05
EEF1A1P5	0,0126557
INPP5A	0,0104334
B3GAT3	0,0258177
RN7SL2	0,0137371
MBOAT2	0,0030387
KB-318B8.7	0,0100642
UBE2Q2P1	0,0054117
TOB1	0,0389923
RP11-701H24.3	0,0043828
SLC16A10	0,0016996
RP1-22N22.1	0,0312884
LINC01252	0,0364194
KCTD1	9,083E-05
AC012066.1	0,0018346
PMPCA	0,0402407
RNF38	0,0001187
GLTP	0,0320172

Lesion specific genes

TMEM151A	0,0197608
ARF1P2	0,003152
PAK1	0,0177294
AHSA2	0,0212274
CCDC177	0,0264476
FRAS1	0,0138891
DCAF5	0,0150743
ATP9B	0,009548
DAZAP1	0,0086752
CTA-204B4.2	0,0435624
SRP72P2	0,0030733
GYS1	0,0117148
RP11-631M6.2	0,0248097
TRIM33	0,0400049
HCN1	0,0025463
ABALON	0,0077615
RPS5	0,042286
HMGN1	0,0042327
ELOVL5	0,0301453
LRFN5	0,0011411
ENTPD3	0,0496326
RP11-364L4.1	0,0055659
KCNA1	0,0001673
PPP3CA	0,0002173
OXLD1	0,0081201
DDB2	0,0053363
SLC27A3	0,0447273
PLEKHO1	0,0287288
GAPDHP55	0,001562
TRUB2	0,0210082
RP11-89N17.4	0,0391722
MSH6	0,0259114
LINC01410	0,0014723
RBFOX1	2,648E-05
PCDH7	0,0050946

Lesion specific genes

ABHD2	0,0247496
CDS1	4,103E-05
RHEB	0,0312239
WDR19	0,0469416
GUCA1B	0,0229338
RAD18	0,0096765
AC079117.1	0,0256303
CCDC181	0,0007553
KIAA0355	0,0228614
ABI2	0,0225011
PCDHGA1	0,0391305
POLDIP3	0,0086831
AF131216.5	0,0403635
TP73-AS1	0,0267183
HPS4	0,0015271
RP11-274B21.1	0,0083194
GDNF-AS1	0,0296473
FGF14-AS1	0,0022058
RP5-1119A7.17	0,0002266
HLF	0,0001332
TLE2	0,0041298
GPRC5C	0,0216069
AHCYL1	0,0241895
AC010976.2	0,0005261
PBX1	0,0096463
PLEKHM1P	0,0367646
RP1-168L15.5	0,0177321
IQSEC2	0,0391305
NT5C3B	0,0304668
DUBR	0,0311339
SCN1A	0,0002771
ASAP2	0,0225021
SCN3A	0,0051134
DEF8	0,0373131
ZFPM2	0,0093287

Lesion specific genes

SLC35F1	0,0034915
RP11-473C19.1	9,366E-06
C21orf49	0,0272948
COG4	0,0031026
ASAP1	0,016019
HNRNPA0	0,0073644
ZNF354C	0,0224056
IKBKG	0,0018072
FRMPD4	0,0223312
GALNT17	0,0093296
AMMECR1	0,0133352
WDR91	0,0466718
HAGH	0,0071292
FBLL1	0,0293434
AGO2	0,0338608
RIMBP2	0,0156241
CSPP1	0,0253068
THY1	0,0063026
KIT	0,0016035
TAS2R12	0,0079499
EPHA7	0,0008757
SCN4B	0,001235
SPAG9	0,0312309
PEX5L-AS2	0,0478029
DGCR5	0,0283665
GRIA1	0,0004472
WNT5A	0,0342803
TMEM176A	0,0224938
RNF150	3,419E-06
RP11-10N23.2	0,0439605
CD200	0,0366062
GAD2	0,0156737
NPY1R	0,0163293
GCNT2	0,0102251
GABRG3	0,0089704

Lesion specific genes

RP11-69E11.4	0,0260991
SURF4	0,0315977
CTC-524C5.2	0,0346991
SGCZ	0,0258111
APTX	0,0199533
ZNF514	0,0264669
ADCK2	0,0479502
LRIG1	0,0379704
POFUT2	0,006908
PCDH11X	0,020405
TMC6	0,0209455
DRP2	0,0011384
ZXDC	0,0186035
SLC31A2	0,0008681
GPR182	0,0072173
CTD-2228K2.7	0,0312576
PCDHB13	0,0312884
ASB1	0,0222399
PCDHA7	0,0388293
UCK1	0,0341309
RPS10P14	0,0280022
PPP1R3F	0,0457144
C1orf220	0,0221767
RP11-57B24.2	0,0314131
ASAH2B	0,0172975
RPL23AP64	0,0009528
ZDBF2	0,0045388
POU2F2	0,0195215
AC122129.1	0,0177033
HIST2H2AC	0,0135203
BEND5	0,0372266
EPHB1	0,0005479
AC006262.4	0,0165907
RECQL5	0,0005845
HSD17B7P2	0,0018791

Lesion specific genes

NR2F2	0,0131507
SRRM1	0,0493714
MOCS1	0,0022055
CACNA2D2	0,0193858
ECT2	0,0290535
LINC00402	0,0098141
GLCCI1	0,0009871
B3GALT2	0,0317306
CCDC167	0,0255259
CGREF1	0,0100911
BLNK	0,0243421
TMEM178B	0,0148844
SOGA1	0,0370051
KDM5B	0,0290496
FAM53B-AS1	0,0489478
SLC22A23	0,0044416
MAP3K21	0,0007711
CTNS	0,0008881
GDNF	0,0136779
STK36	0,0161344
MZT2B	0,0251726
GSTM1	0,0335378
AMFR	0,0063814
LINC02389	0,0023875
DKK3	0,0392441
MAPK8IP1P2	0,0002307
PPP2R5B	0,012289
ATG2A	0,0353163
RPGRIP1L	0,0181679
OSGIN1	8,398E-05
PTPRF	0,0104741
FXR2	0,0111247
RP11-728K20.2	0,0346741
SNX9	0,0205015
SLC25A17	0,0359873

Lesion specific genes

TTC39A	0,0125411
MCM7	0,0037278
HIST1H4D	0,0023971
SAMD11	0,0025203
MRPL55	0,0278666
EML6	0,0128167
TTC8	0,0212731
TENM3	0,0094374
ZNF408	0,0192822
TUSC3	0,0141619
GABRD	0,0075782
LOC101927950	0,04953
EVI2B	0,004412
PHF2	0,0139049
PID1	0,0297408
CRIPAK	0,0010715
AFDN	0,0170943
RALGPS2	0,0011596
C2CD2L	0,0131359
SNRK	0,0051642
DLGAP3	0,0187003
IL17RA	0,0274118
MPP5	0,0348366
CAMK4	0,0006009
RP11-739P1.2	0,003366
RBFOX2	0,0002204
ZNF26	0,0036873
DNAJB2	0,0354528
KB-1517D11.4	0,0105674
RP11-195B17.1	0,0004968
PKIA	0,0389122
RP11-53B2.4	0,0027077
UBE2Z	0,0216831
AC103563.8	0,0032617
GRM7	0,0301725

Lesion specific genes

STS	0,0006808
RP11-29H23.5	7,09E-05
SMIM5	0,0135281
RP11-1017G21.5	0,0017757
HNRNPCP6	0,0019582
RELB	0,0124219
PTPRM	0,0486378
MARCH4	0,0009955
ALG5	0,0278588
NCOA2	7,919E-06
FAM118B	0,0077293
ASPHD2	0,0006075
FSBP	0,0313315
ZMAT2	0,0480217
EIF4H	0,0008659
KCNH7	5,385E-05
NUAK2	0,0019967
MANSC1	0,0002195
MIRLET7BHG	0,0453398
PMS2P3	0,0051547
CDKL5	0,0046606
COQ4	0,0042491
LY86-AS1	0,0270602
ZNF418	0,0012774
DDX25	0,0433167
CYP51A1P3	0,001173
PABPC1P11	0,0011283
LOC102546294	0,0024328
SCMH1	0,0065707
PLPPR4	0,0041242
HTR2A	0,0072746
R3HDM1	0,0003993
APC	0,0029989
PEX12	0,0304845
MACROD2	0,0062901

MOCS2	0,0008689
RGS7BP	4,31E-05
CTB-176F20.3	0,0264476
RP11-480A16.1	0,034175
CYFIP2	0,0014723
TMEM56	0,0282656
RCC1L	0,0210929
MAP3K11	0,0287924
RPS9	0,0042037
LRTM2	0,0002559
AC009505.2	0,0003005
TP53BP2	0,0103956
BMP8B	0,019096
RP11-421L21.3	0,000627
AP000857.2	0,003073
PPIP5K1	0,0025882
BEAN1	0,0410292
L3MBTL1	3,396E-05
LOC440084	0,0165436
DCHS2	0,0014723
SNORD64	0,0058906
ALOX12-AS1	0,0016243
SENP1	0,0140581
UTP4	0,0210635
NDUFAF3	0,0125569
CDK5RAP2	2,044E-06
PABPC1P7	0,0011066
TSC22D1-AS1	1,579E-05
FASN	0,0171791
EPN2-AS1	0,0032879
RP11-243J16.7	0,0112867
MPPE1	0,0003734
SQSTM1	0,0099146
ZNF274	0,0032528
C7orf26	0,0189457

Lesion specific genes

ZNF397	0,0187767
ANXA13	0,0404527
KLHL23	0,0099318
CNOT4	0,0008382
SLC26A8	0,0337263
CTD-3088G3.8	0,0001932
SNTG1	1,041E-05
KCNA2	0,0011119
CDKN1B	0,0016237
SEMA3A	0,0390894
BTRC	0,0441228
TRIM3	0,0232558
SLC8A3	0,0006892
RP11-63G10.3	0,0229757
RP11-416I2.1	0,0055932
RNF180	0,0451871
NTNG2	0,0035217
SRPK3	0,033793
REC8	0,0040028
NRIP1	0,0402594
GPR52	0,0096891
MAU2	0,004931
RP5-890O3.9	0,0079883
CATSPERG	0,0022345
AC144530.1	0,0093113
PPP2R1B	0,0165954
TANC1	0,0234135
RP11-175B9.2	0,0221098
NCOA7-AS1	0,0103551
RP11-192H23.4	0,0053578
ANKS6	0,0029989
ASB3	0,0199898
OGG1	0,0046285
LITAF	1,395E-06
TUBA1B	0,0193812

Lesion specific genes

RP11-253I19.3	0,0131542
PTP4A2P2	0,0213261
BRF1	0,0027552
CHCHD7	0,00078
RP11-112J3.16	0,0373283
KCNB2	0,0002598
CUEDC2	0,010043
SS18L2	0,0102251
GABRB1	0,0388678
TAS2R19	0,0467353
INF2	0,0025717
CRB2	0,0055227
LYST	0,0322437
ARL10	0,016529
RAD51-AS1	0,0148589
DLGAP1-AS5	5,183E-05
JAKMIP1	0,0143726
PRORSD1P	0,0110306
RIMS3	0,0174395
CALR	0,026106
CCDC158	0,0178372
RP11-216N14.7	0,011609
ZRANB1	0,0423345
RGS7	0,0025353
SGTB	0,0224785
C1orf122	0,0054918
LINC02024	0,0427266
CD2AP	0,0346741
MYH10	0,0003018
KIF5A	0,0045045
ASCC2	0,0213447
CYB561D2	0,0070092
RSAD1	0,005392
LRRC34	0,0424368
CDH26	0,0003066

Lesion specific genes

RP11-71N10.1	0,0057108
SCAI	0,0121391
NRG2	0,049258
RP11-66B24.1	0,0169221
SLC12A9	0,0317312
TPRN	0,0231494
CD46	0,0031925
RP11-44D5.1	0,0031925
SNORA47	0,0372158
ERICH3	0,0004297
TRERF1	5,945E-06
RP11-798L4.1	0,0459208
TINF2	0,0019047
BHLHB9	0,0153133
NTRK3	0,0010336
MIAT	0,0199918
POLR3C	0,025258
SLC35F3	0,0027543
AC006019.4	0,009518
RP11-29G8.3	0,0153187
CTD-2555K7.2	0,0165224
RPS6KB2	0,0052929
TXLNA	0,0200694
MCEE	0,0053933
INTS4P1	0,0001088
FREM2	0,0138838
DROSHA	0,0278295
COASY	0,0246412
DOT1L	0,016529
AC112229.7	3,883E-05
RPRD2	0,0031358
MED30	0,0155377
CCDC93	0,0153646
LMTK2	0,0076278
YEATS2-AS1	1,983E-05

Lesion specific genes

LAMP5	4,78E-05
TMSB4Y	0,0472068
KATNBL1	0,0112657
PHF5A	0,0086205
TAGLN	0,0413247
C10orf88	0,0415
DNAH10OS	0,0031533
KCNK2	0,0024694
TSPOAP1	0,0137369
HNRNPA1P68	0,0495418
CNNM1	0,0027553
AC012513.4	0,0345291
DOCK9	0,042948
RRP8	0,0256894
RORA	0,0012754
CYP4X1	0,0022014
TBC1D13	0,0202071
EIF1B	0,0422958
RP4-534N18.2	0,0098904
SPATA25	0,0016924
METTL9	0,0121994
ANKRD19P	0,0002147
WIPF2	0,0160851
PLXDC2	0,0103032
ELAVL4	0,0083534
CDH9	0,0008953
ZNF23	0,0143313
NAT9	0,0023007
CACNG8	0,0122618
EIF4A1	0,004328
RP1-78O14.1	0,0028583
ANKRD30B	0,0024116
PCDH19	0,0014344
CLINT1	0,0092763
LEPROTL1	0,0158285

Lesion specific genes

AC007740.1	0,0003993
NIN	0,0199898
HOMER1	0,0492304
C11orf87	0,0035696
PTGR1	0,0344344
FNDC5	0,0115776
SERPINB6	0,0033237
SCN2A	0,0088028
RAB40A	0,0229625
CAD	0,0113615
AC007246.3	0,0402873
RPAIN	0,0391896
ARL14EP	0,0350008
ST8SIA3	0,0025499
NDUFS5P1	0,0414482
C19orf48	0,033074
PLPP6	0,0013712
ALOX12P2	0,0038005
CSAD	0,0020923
ANKRD50	0,0443001
ABAT	0,0010264
LHX6	0,0178357
RP11-760H22.2	0,0461515
IGLON5	0,0391365
SRPRB	0,0297282
MARC2	0,0402712
UBAP2L	0,0499254
PRPF18	0,015491
SLF2	0,0125173
RP11-286E11.2	0,0037389
KIF15	0,003016
RP11-355F16.1	0,0176539
ERP44	0,032218
PCDHA3	0,0482859
C6orf165	0,0331262

Lesion specific genes

NSA2	0,0008247
KCNH5	0,0025952
LOC100128494	0,0017518
GPC2	0,0342588
MIR124-2HG	0,0023642
RP1-67A8.3	0,0016472
RGS9	0,0181529
RPL23	0,0403941
FAM84A	0,0001313
CLVS1	7,265E-05
STAT2	0,0014621
SPART-AS1	0,0294777
ROBO2	0,0114649
GNA14	0,0105272
ALMS1P1	0,0153497
OSCP1	0,0001341
LLPH	0,0281394
CYTH3	0,0016024
PLEKHA8	0,0033857
C19orf57	0,0384535
RP5-821D11.7	0,0066936
KIAA2013	0,0314306
KIAA0319	0,0014588
HDHD5	0,0016237
ARHGDIA	0,0249588
GCAT	0,0082972
TRAPPC4	0,0003749
UNC13C	0,020914
DNM1P47	0,0001371
CAB39L	0,0056895
PTPN3	0,0028439
ART3	0,0021889
FOXP1	0,000114
EPHB6	0,0022216
CXXC4	0,0123194

Lesion specific genes

RGMA	0,0162176
SSX2IP	0,0197688
POMT2	0,0097101
CABLES1	0,0065751
TUBGCP6	0,0333296
MTND4P14	0,0015677
BCL11A	0,0007806
RP11-1105O14.1	3,704E-05
TMEM254	0,0008639
VIPR1	0,0042605
PTK6	0,0001773
DDR1	0,002827
ADPGK-AS1	0,0053507
C6orf47	0,0258633
PSME3	0,0326859
NME3	0,0029898
RP11-202D18.2	0,0040989
CYP46A1	0,0013823
CHN1	0,0003834
RRNAD1	0,0325219
PMM2	0,0115953
METTL3	0,0186179
ANXA4	0,0134643
DCP1B	0,0012344
RPS14	0,0409222
ONECUT2	0,0001036
LGR4	0,0256317
UBE2Q1	0,0179183
JPH3	0,0325219
RP11-73C9.1	0,0071407
C20orf204	0,0076715
SMARCAL1	3,48E-05
SCARNA5	4,803E-05
MAT2A	0,0150682
SEMA4C	0,0023358

Lesion specific genes

NDRG3	0,040884
RNF6	0,0102251
CACNA1I	0,0404237
PHF21A	0,0486287
EAF2	0,0296413
TRMU	0,0035068
OSBPL5	0,0012831
EXOC6	0,0150079
PIK3IP1	0,0284385
